# Safety and immunologic impact of neoadjuvant/adjuvant GVAX, cyclophosphamide, pembrolizumab, and anti-CSF1R agent IMC-CS4 in pancreatic adenocarcinoma

**DOI:** 10.3389/fimmu.2026.1715761

**Published:** 2026-03-09

**Authors:** Arielle Urman, Yingjun Ding, Jennifer Durham, Hao Wang, Hanfei Qi, Amol Narang, Richard Burkhart, Jin He, Dung Le, Daniel Laheru, Elizabeth Thompson, Elizabeth Jaffee, Katrina Purtell, Charmaine Waisome-Stephens, Meizheng Liu, Lei Zheng, Ana De Jesus-Acosta

**Affiliations:** 1 The Sidney Kimmel Comprehensive Cancer Center at Johns Hopkins, Department of Oncology, Johns Hopkins University School of Medicine, Baltimore, MD, United States; 2 The Bloomberg-Kimmel Institute for Cancer Immunotherapy at Johns Hopkins University School of Medicine, Baltimore, MD, United States; 3 The Cancer Convergence Institute at Johns Hopkins University School of Medicine, Baltimore, MD, United States; 4 The Pancreatic Cancer Precision Medicine Center of Excellence Program at Johns Hopkins University School of Medicine, Baltimore, MD, United States; 5 Division of Biostatistics and Bioinformatics, Department of Oncology, Johns Hopkins University School of Medicine, Baltimore, MD, United States; 6 Department of Radiation Oncology, Johns Hopkins University School of Medicine, Baltimore, MD, United States; 7 Department of Surgery, Johns Hopkins University School of Medicine, Baltimore, MD, United States; 8 The Skip Viragh Pancreatic Cancer Center, Johns Hopkins University School of Medicine, Baltimore, MD, United States; 9 Department of Pathology, Johns Hopkins University School of Medicine, Baltimore, MD, United States; 10 Mays Cancer Center at the University of Texas Health San Antonio MD Anderson Cancer Center, San Antonio, TX, United States

**Keywords:** borderline resectable pancreatic cancer, CSF1R (Colony stimulating factor 1 receptor), immunotherapy, neoadjuvant therapy (NAC), pancreas adenocarcinoma

## Abstract

**Background:**

We previously reported that an increased M1/M2 ratio and decreased PDL1+ M2- like tumor-associated macrophages (TAM) are associated with longer survival in patients with pancreatic adenocarcinoma (PDA). Targeting M2-like macrophages may improve patients’ outcomes. In this pilot study, we hypothesized targeting M2-like macrophages, regulated by the colony stimulating factor-1 (CSF1) pathway, would be safe and induce an intratumoral immune response in patients with PDA.

**Methods:**

We tested perioperative combination immunotherapy (CI) with GM-CSF-secreting allogenic pancreatic tumor cell vaccine (GVAX)/cyclophosphamide (CY), pembrolizumab (Pem), and CSF1 receptor blockade (IMC-CS4) in patients with PDA. Patients received two neoadjuvant cycles of CI followed by surgery and four adjuvant cycles of CI. Subsequently, they received a booster Pem every 3 weeks and GVAX/CY every 6 months, for up to one year. The co-primary endpoints were safety and immune response in paired biopsies.

**Results:**

Nine patients were enrolled and treated in this study. We observed two immune related grade 3/4 AEs (diarrhea and rash). Comparison of paired biopsies showed five of eight evaluable patients met the immunologic endpoint with >80% increase in CD8+ T cells. The increase was at least 1.8 times the baseline median absolute deviation.

**Conclusion:**

CI has a manageable safety profile and leads to increased intratumoral cytotoxic effector T cells.

**Clinical Trial Registration:**

https://clinicaltrials.gov/study/NCT03153410, identifier NCT03153410

## Introduction

Pancreatic adenocarcinoma (PDA) is one of the most lethal malignancies worldwide, with an overall 5-year survival rate of only 12% in the United States (1). Its increasing incidence highlights the need to improve outcomes. Chemotherapy remains the standard of care (SOC) therapy, but rarely leads to long term survival. Immunotherapy with immune checkpoint inhibition (ICI) has revolutionized cancer care and dramatically improved survival outcomes in a variety of cancer types (2–4) but its effectiveness in PDA is limited. Multiple clinical trials that tested ICI monotherapy and combination therapy in PDA yielded negative results (5–7).

A key barrier to immunotherapy’s effectiveness in PDA is its highly immunosuppressive tumor microenvironment (TME) (8–11) comprised of a dense extracellular matrix and immunosuppressive cellular populations that allow cancer cells to evade immune surveillance, progress and metastasize (12, 13). Compared to normal pancreatic tissue, infiltration of neutrophil, macrophage, regulatory T cell (Treg), cancer-associated fibroblast, and myeloid derived suppressor cells (MDSCs) are significantly increased in PDA tissue, while the infiltration of immune effector cells such as CD8+ cytotoxic T and natural killer cells are typically reduced (14–16). *In vivo* depletion of myeloid cells in murine PDA transgenic models has been shown to result in tumor growth arrest and CD8^+^ T cell–dependent tumor regression (17).

Our group has studied sequential immunotherapy combinations to enhance the immunogenicity of the PDA TME by increasing T cell infiltration and prime it for a response to ICI. GVAX (GM- CSF-secreting allogenic pancreatic tumor cell vaccine) was developed to expose patients to many tumor antigens and induce an immune response (18). We demonstrated that GVAX induces a peripheral T cell response and that a single dose of neoadjuvant GVAX induces development of lymphoid aggregates (LAs) in PDA tumors within 2 weeks of administration. The LAs contain not only T and B cells, but also MDSCs (19, 20). An increase in PDL1 expression is seen in both tumor epithelial cells and myeloid cells within the LAs, suggesting that the vaccine may prime the PDA TME to respond to ICI (19, 20).

We subsequently tested in patients GVAX combined with ICI, as preclinical studies combining anti-PD1 antibodies with GVAX noted enhanced effector T cell infiltration in PDA tumors and increased cure-rates in PDA-bearing mice (21). Correlative studies of pre- and posttreatment PDA specimens from our platform trial evaluating GVAX with or without nivolumab demonstrated that higher densities of tumor associated neutrophils (TANs) following combination therapy portended poorer overall survival (OS), while increased CD137+ T cells correlated with increased OS (9, 22).

We then tested the combination of neoadjuvant stereotactic body radiotherapy (SBRT), GVAX, and pembrolizumab (Pem) in locally advanced PDA and found increased M2:M1 tumor- associated macrophage (TAM) polarization was associated with inferior survival (23). Based on these findings, we hypothesized that targeting M2-like macrophages, regulated by the colony stimulating factor-1 (CSF1) pathway, would be a rational target to improve outcomes.

CSF1-receptor (CSF1R) inhibition has been shown to upregulate T cell checkpoint molecules, including PDL1 and CTLA4, and reprogram TAMs in PDA models (24). Our preclinical study showed that triple combination therapy with anti-CSF1R antibody before and after GVAX and anti-PD1 antibody enhanced survival rate compared with doublet therapy with GVAX and anti-PD1 antibody (25). We also found that the triplet combination administered after gemcitabine resulted in increased tumor regression and improved survival compared to gemcitabine alone (25). These findings provided the rationale for testing this combination of immunotherapy in PDA patients.

To translate the above preclinical findings, we investigated the safety and intratumoral immune response of neoadjuvant/adjuvant combination immunotherapy with GVAX/CY, Pem, and CSF1R blockade with IMC-CS4 in patients with resectable or borderline resectable (BR) PDA.

## Methods

### Study design

This was a single-center, open-label pilot study to evaluate the safety, immune effect, and early signals of clinical activity of the combination of GVAX/CY, Pem, and IMC-CS4 in neoadjuvant and adjuvant settings in patients with resectable and BR PDA, following completion of standard neoadjuvant chemotherapy.

### Study population

Eligibility criteria included age 18 years or older; histologically confirmed PDA staged as resectable or BR based on NCCN or surgical guidelines and discussions at the Johns Hopkins Hospital (JHH) pancreas tumor board; an Eastern Cooperative Oncology Group (ECOG) performance status of 0-1; measurable disease per Response Evaluation Criteria in Solid Tumors (RECIST), version 1.1; and adequate hematologic, hepatic, and renal function. Eligible patients were required to have completed standard neoadjuvant chemotherapy with or without SBRT. The key exclusion criteria included prior immunotherapy, autoimmune disease, or concurrent uncontrolled illness. Patients must have completed chemotherapy >14 days and/or radiation >28 days prior to enrollment. Full lists of the inclusion and exclusion criteria are provided in the protocol.

### Treatment schema and assessments

**Figure 1:**
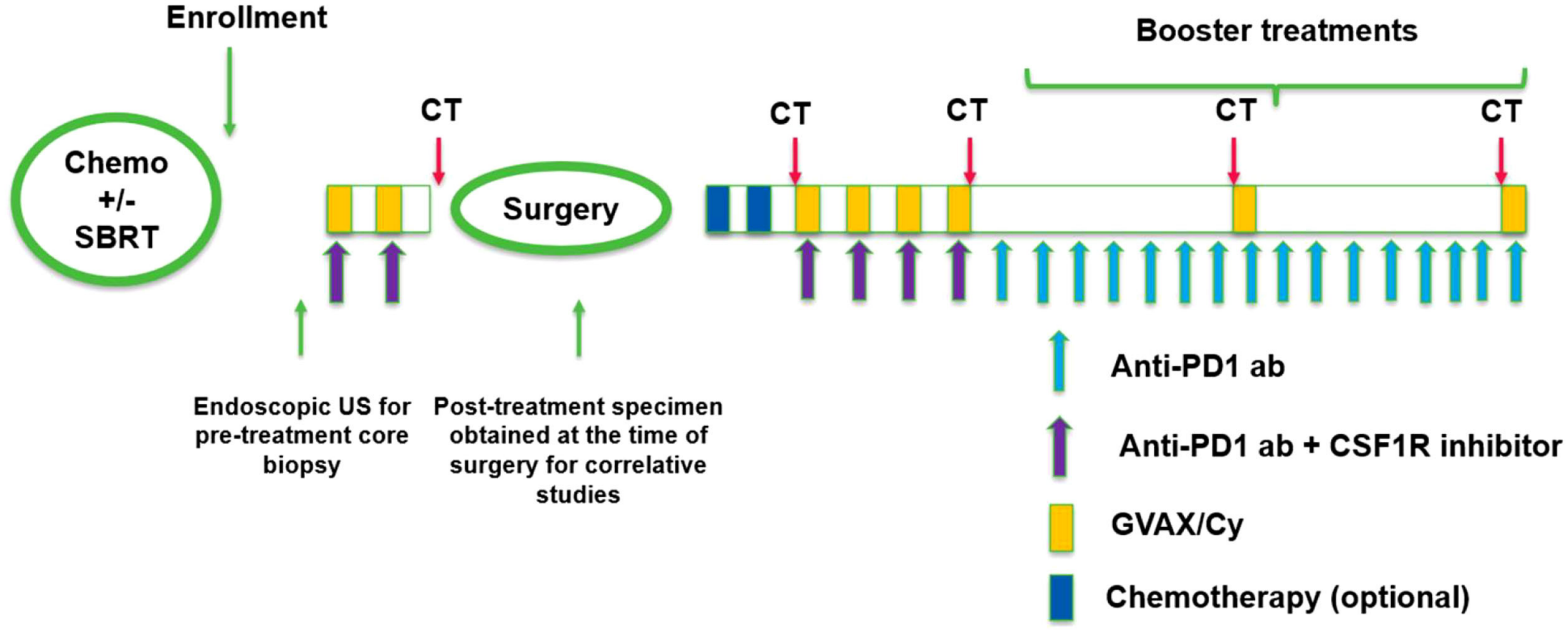
Study schema.

Potential candidates were identified while receiving standard neoadjuvant chemotherapy with 5- fluorouracil, irinotecan and oxaliplatin (FOLFIRINOX) or gemcitabine and nab-paclitaxel. A screening visit occurred 7 days after neoadjuvant chemotherapy completion. Eligible patients underwent EUS guided pretreatment research core biopsy of the pancreatic tumor within 14 days of CI initiation. The first dose of CI was given after neoadjuvant chemotherapy completion. Treatment with CI as detailed in **Figure 1** consisted of neoadjuvant CI in 21-day cycles consisting of CY 200 mg/m^2^ administered intravenously (IV) and pembrolizumab 200 mg IV on day 1, IMC- CS4 on days 1, 8, and 15, and GVAX administered as six intradermal injections on day 2. Immunomodulatory CY was administered prior to GVAX as a standard pre-vaccination treatment at the time the trial protocol was developed (26). Patients received a total of two cycles of CI prior to surgical resection.

Following the two neoadjuvant CI cycles, patients without radiographic progression underwent surgical resection. In the 4–8 weeks after surgery, patients were permitted to receive adjuvant chemotherapy, at the discretion of the primary oncologist. Patients were re-screened within 90 days after surgery in the absence of adjuvant chemotherapy and within 180 days after surgery if they received adjuvant chemotherapy. Those who continued to meet the study criteria received 4 cycles of adjuvant CI. After this, patients received Pem boosters every 3 weeks for 12 cycles. and CY/GVAX boosters every 18 weeks. The CY/GVAX boosters were given with booster doses 6 and 12 of Pem. The schedule is shown in **Figure 1**.

Patients were assessed for local or distant disease recurrence by CT scan prior to enrollment, prior to surgery, following surgery and immediately prior to starting adjuvant CI, following adjuvant CI and prior to starting booster therapy, halfway through booster therapy, and again at the end of booster therapy.

### Multiplex immunohistochemistry

Multiplexed IHC was used to evaluate the intratumoral immune response. Pre-treatment core biopsies and post-treatment surgically resected tumor specimens were prepared as formalin-fixed paraffin-embedded (FFPE) tissue slides. The slides were stained with hematoxylin (CS70030-2, Dako) and scanned using NanoZoomer (Hamamatsu). After scanning, the slides were de-covered in TBST with two minutes of agitation and incubated with hydrogen peroxidase (MKCN2357, Sigma) for 30 minutes for endogenous peroxidase blocking. The heat-mediated antigen retrieval step was accomplished using Antigen Retrieval Citra (HK0809K, BioGenex) and microwave heating. Nonspecific binding was blocked by incubating the slides in blocking buffer containing 5% normal goat serum (G9023, Sigma) and 2.5% BSA (A9647-500G, Sigma) for 10 minutes. Subsequently, the slides were ready for staining procedures, including incubation with the primary and secondary antibodies and development of the AEC solution. The primary antibodies are commercially available and antigen-specific. The secondary antibodies (NICHIREI BIOSCIENCES) are horseradish peroxidase (HRP)-conjugated and react with the AEC solutions containing chromogenic substrates to show color. The AEC solution development time varies depending on the targeted protein marker and was optimized to show the best contrast between positive and background staining. Unlike traditional single IHC which only stains for one marker, mIHC performs antibody and AEC color-stripping procedures to allow for the next staining cycle. Stripping was accomplished by incubating the slides with alcohol and repeating the same antigen retrieval step. The primary or secondary antibodies used, the dilution ratio of antibodies, and the length of antibody or AEC solution incubation time varied between each cycle depending on the targeted markers (**Supplementary Table 1**). In our experiment, we stained 15 markers including CD68, PD-1, PD-L1, CD163, T-bet, Granzyme, CSF1R, Foxp3, CD4, CD8, CD66b, CD3, CD45,

CD56 for 15 cycles plus hematoxylin nucleus staining, totaling 16 staining cycles.

Using the sequential mIHC technique, each FFPE slide was examined for marker expression and the corresponding immune cell-subtype. Immune cell subtypes corresponding to the 15 immune cell biomarkers were defined and gated, as described in **Supplementary Table 2**. Cells were considered positive through manual gating by comparing the cytometry results of the negative control staining using FCS software. After the 16 staining cycles, each FFPE tissue slide was digitally scanned as whole-tissue section images. Instead of analyzing the entire tissue section, several tumor regions within the whole tissue section were selected as our Regions of Interest (ROI), and the percentage of cells expressing specific markers inside each ROI was analyzed. To quantify the percentage of cells that were positively stained with a particular marker, scanned images were processed using a computational analysis workflow that included three major steps: coregistration, visualization, and final quantification. Coregistration aims to align all the scanned images and was achieved by selecting 200x200 pixel landmark images using Image Scope software and running these landmark images with a pre-established coregistration pipeline in CellProfiler software. After aligning all the scanned images, Image Scope software was used again to exact one to four 3000x3000 pixel images from each aligned scanned whole-tissue section image depending on the area of tumor regions designated as ROIs. The selected ROIs were all located within the tumor regions that were cycled by a specialized pathologist. Subsequently, these ROIs were treated with a pre-established AEC extraction and hematoxylin color deconvolution algorithms using ImageJ Software to extract only the staining color but exclude unwanted background colors. Finally, background-color-excluded ROIs were processed with a pre-established cytometry pipeline using the CellProfiler software to segment tissues and quantify the staining intensity on single-cell bases. The generated pixel intensity and cell segmentation files were compatible with FCS Express cytometry data analysis software. Through manual gating, FCS software provided numeric percentages of positive staining for each marker within each ROI. The FCS software also calculated the corresponding cell subtype density for each ROI based on the combination of specific markers (**Supplementary Table 2**). After averaging the data for all ROIs, the mean percentage of cells expressing specific markers and the corresponding cell subtypes for each slide specimen were calculated.

### Statistical considerations

The co-primary endpoints were safety and immune response. The safety of IMC-CS4 was assessed using a two-part dose-escalation procedure. Toxicities were graded using the Common Toxicity Criteria for Adverse Events Version 5.0 (CTCAE v.5). The first three patients received IMC-CS4 at dose level 1 (75 mg) and were monitored for unacceptable toxicities during the first two cycles of immunotherapy and subsequent surgical resection. If ≤1 patient develops unacceptable toxicity, the remaining patients would be given IMC-CS4 at dose level 2 (100 mg). The Bayesian stopping principle was used based on the rate of grade 3 or 4 toxicity. The combination therapy would be considered safe if the rate of grade 3 or 4 toxicity was lower than 30%. Postoperative complications within 30 days of surgery were defined based on the Clavien- Dindo Criteria (27).

The primary immunologic endpoint was the change in CD8+ T cell density in pretreatment versus posttreatment surgical tumor specimens. At the individual patient level, a significant treatment- related immunologic effect (i.e immune response) in tumor infiltrating CD8 density was defined as a >80% increase or decrease (post vs pre-surgery) AND such increase/decrease is at least 1.8 times the baseline median absolute deviation (MAD), where the baseline MAD is defined as the median of the absolute deviation from the median calculated across patients and is thus a more robust measure of the variability than sample standard deviation. This approach and cut- offs were pre-specified at the time of study design. A sample size of up to 12 patients was chosen for enrollment to achieve a sample size of 8 evaluable patients with paired pre and post-treatment specimens for immunologic endpoint assessments. Immune response was defined as a >80% increase or decrease in tumor-infiltrating CD8+ T cell density and such change is at least 1.8 times the baseline median absolute deviation (MAD). Composite endpoints, such as the median of the absolute deviation from the median across patients, serves as a more robust measure of variability than standard deviation (28–30). If 3 of 8 patients demonstrate an immune response, the 90% confidence interval of response rate is [11%, 71%]. Ruling out a lower limit of 10% for immune response rate is considered clinically meaningful. The density of each immune cell subtype was defined by calculating its percentage among all CD45+ cells in the tumor areas.

Descriptive statistics were used to report immune parameters in pretreatment biopsy specimens and that in matched posttreatment specimens. Paired t-tests were used to assess the change of expression of each immune cell subtype. Tests were two-sided, and the differences were considered significant when the p-value < 0.05.

Secondary endpoints included disease free survival (DFS), OS, objective response rate as determined by imaging studies, surgical resectability, and pathologic response (pathR) using the tumor regression grading system for PDA after neoadjuvant chemotherapy of the College of American Pathologists (31, 32). DFS was defined as time from the start of the study treatment until radiographic recurrent disease and/or death. Individuals were censored with respect to DFS at the date of last restaging scan if they had no evidence of disease. OS was defined as time from the start of study treatment until death from any cause. DFS and OS calculations were performed using Kaplan-Meir analyses. Survival follow up continued until August 2023.

### Study approval

The study was conducted at the Sidney Kimmel Comprehensive Cancer Center at JHH in Baltimore, Maryland, USA between September 2018 and March 2022. The protocol and amendments were approved by the institutional review board at JHH. The trial was conducted in accordance with the International Council for Harmonization Good Clinical Practice guidelines and the principles of the Declaration of Helsinki. Written informed consent was obtained from all the participants before enrollment.

### Data availability

The authors declare that the minimal data set for this study cannot be shared publicly due to ethical and legal restrictions on sharing de-identified data that aligns with the consent of research participants. Current JHU compliance policies require data with no direct consent for public open access sharing be under restricted access. We will provide access through Vivli, an established repository for clinical data that provides open access without a fee restricted to approved researchers under a Data Use Agreement. JHU compliance policy for Vivli requires additional anonymization of certain demographics, including use of age ranges and limiters to outlier values for weight, height, and certain rare diseases, while retaining sufficient value for reference and validation of results. Researchers can request more detailed data from the corresponding author shared though an approved collaboration arrangement.

## Results

### Patient enrollment

A total of eleven patients were enrolled and nine were treated between September 2018 and January 2021 (two withdrew consent prior to the first dose of study therapy). Prior to study enrollment all patients received SOC chemotherapy (n=9). The majority (78%) of patients received FOLFIRINOX as chemotherapy with the remainder receiving gemcitabine and nab- paclitaxel. The median age of the patients was 64 years (range: 47-75). Seven (78%) patients were classified as stage III according to the American Joint Committee on Cancer 8th TNM staging system and clinical BR at diagnosis. Two patients had clinically resectable disease at diagnosis, one of whom was stage IA and the other was stage IB by TNM. All seven patients with BR disease underwent neoadjuvant SBRT. All patients underwent surgical resection with an R0 resection rate of 100%. The complete patient characteristics are presented in **Table 1**.

**Table 1:** Demographics/patient characteristics.

Median age (range) – yr	64 (47-75)
Male sex – no. (%)	4 (44%)
Race – no. (%)	
Asian	1 (11%)
White	7 (78%)
Hispanic/Latino	1 (11%)
Disease stage at diagnosis – no. (%)	
I	2 (22%)
II	0 (0%)
III	7 (78%)
Chemotherapy received – no. (%)	
FOLFIRINOX	7 (78%)
Gemcitabine and nab-paclitaxel	2 (22%)
Prior radiation – no. (%)	
SBRT	7 (78%)
None	2 (22%)
Clinical staging - no. (%)	
Resectable	2 (22%)
Borderline resectable	7 (78%)

### Safety (co-primary endpoint)

All patients received the full course of neoadjuvant combination immunotherapy (CI) without dose adjustments. The first three patients received 75 mg of IMC-CS4 for safety run in and 6 received 100 mg. There were no differences in safety signals between the two dosing groups. The most frequently reported treatment-related AEs were grade 1 or 2 vaccine site reactions, including erythema at the injection site in all patients and pruritus and induration in eight of nine patients. Two immune-related grade 3/4 AEs were reported: grade 3 diarrhea and grade 3 rash. The diarrhea required hospitalization and steroids that were tapered over 4 weeks. The skin rash resolved with oral steroids within one week and was considered an immune-related AE. Grade 1/2 periorbital edema was observed in one third of patients. Grade 1/2 AST elevation was observed in four of nine patients. Complete treatment- and immune-related AE data are shown in **Tables 2**, **3**. No treatment-related deaths occurred.

**Table 2:** Treatment-related AE table.

System organ class preferred term	Grade 1/2	Grade 3/4	Total (N= 9) N (%)
Blood and lymphatic system disorders			
Anemia	1	0	1 (11%)
Endocrine disorders			
Hypothyroidism	1	0	1 (11%)
Eye disorders			
Periorbital edema	3	0	3 (33%)
Gastrointestinal disorders			
Diarrhea	1	1	2 (22%)
Nausea	1	0	1 (11%)
General disorders			
Chills	1	0	1 (11%)
Fatigue	1	0	1 (11%)
Fever	2	0	2 (22%)
Flu-like symptoms	1	0	1 (11%)
Investigations			
Alanine aminotransferase increased	2	0	2 (22%)
Aspartate aminotransferase increased	4	0	4 (44%)
CPK increased	1	0	1 (11%)
Musculoskeletal and connective tissue disorders			
Arthralgia	1	0	1 (11%)
Skin and subcutaneous tissue disorders			
Bruising, vaccine site	1	0	1 (11%)
Erythema, vaccine site	9	0	9 (100%)
Induration, vaccine site	8	0	8 (89%)
Pruritus, vaccine site	8	0	8 (89%)
Rash	3	1	4 (44%)
Warmth, vaccine site	3	0	3 (33%)

**Table 3:** Immune-related AE table.

System organ class preferred term	Grade 1/2	Grade 3/4	Total (N= 9) N (%)
Endocrine disorders			
Hypothyroidism	1	0	1 (11%)
Gastrointestinal disorders			
Diarrhea	1	1	1 (22%)
Investigations			
Alanine aminotransferase increased	1	0	1 (11%)
Aspartate aminotransferase increased	3	0	3 (33%)
Skin and subcutaneous tissue disorders			
Rash	3	1	4 (44%)

### Immunologic endpoints (co-primary endpoint)

We compared baseline pretreatment endoscopic ultrasound (EUS) core biopsies to surgical posttreatment tumor specimens in eight of the nine treated patients. The excluded patient underwent pretreatment EUS but obtaining the biopsy was deemed unfeasible.

**Figure 2:**
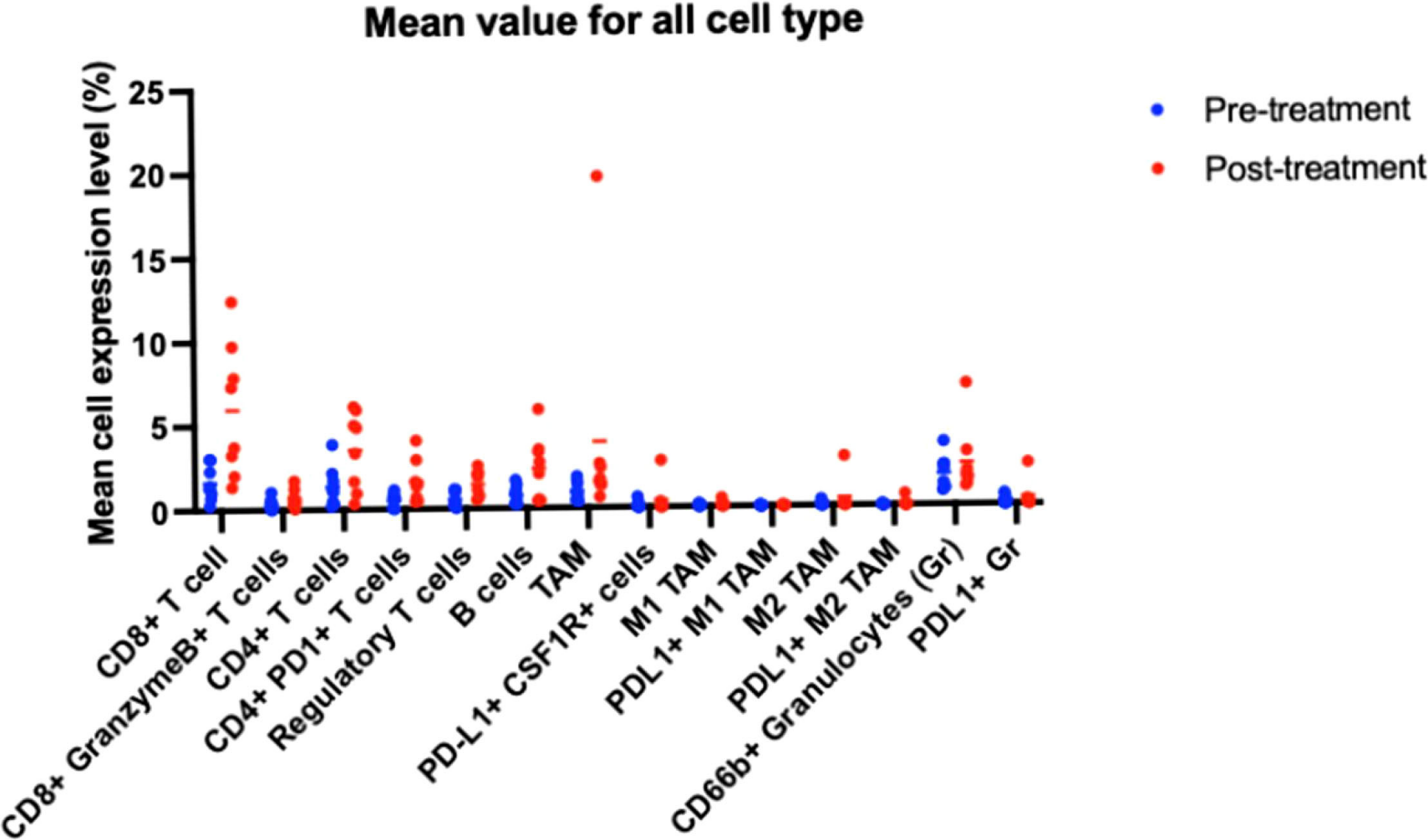
Mean density of immune cell subtypes.

**Figure 3:**
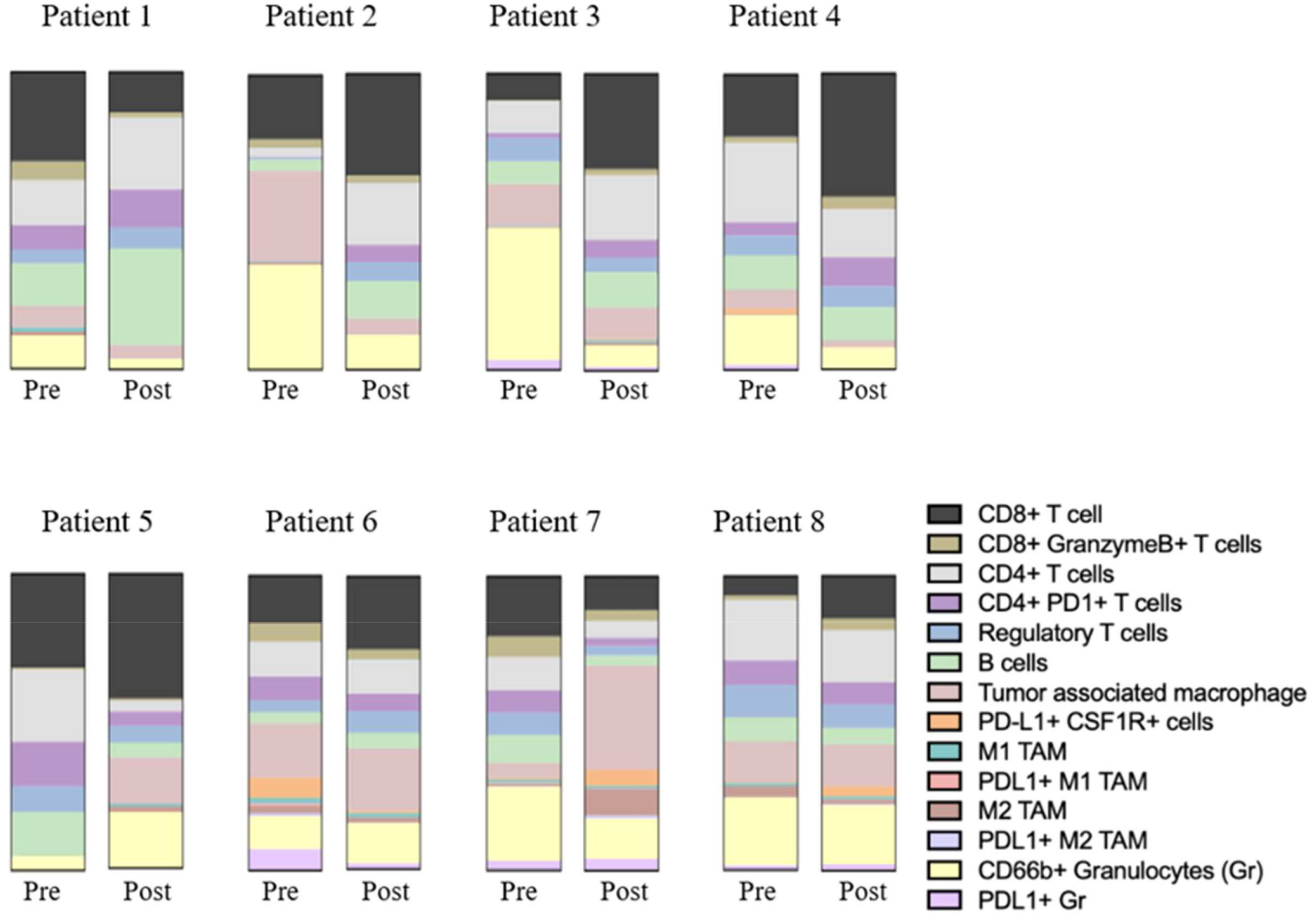
Composition of immune cell subtypes before and after treatment.

**Figure 4:**
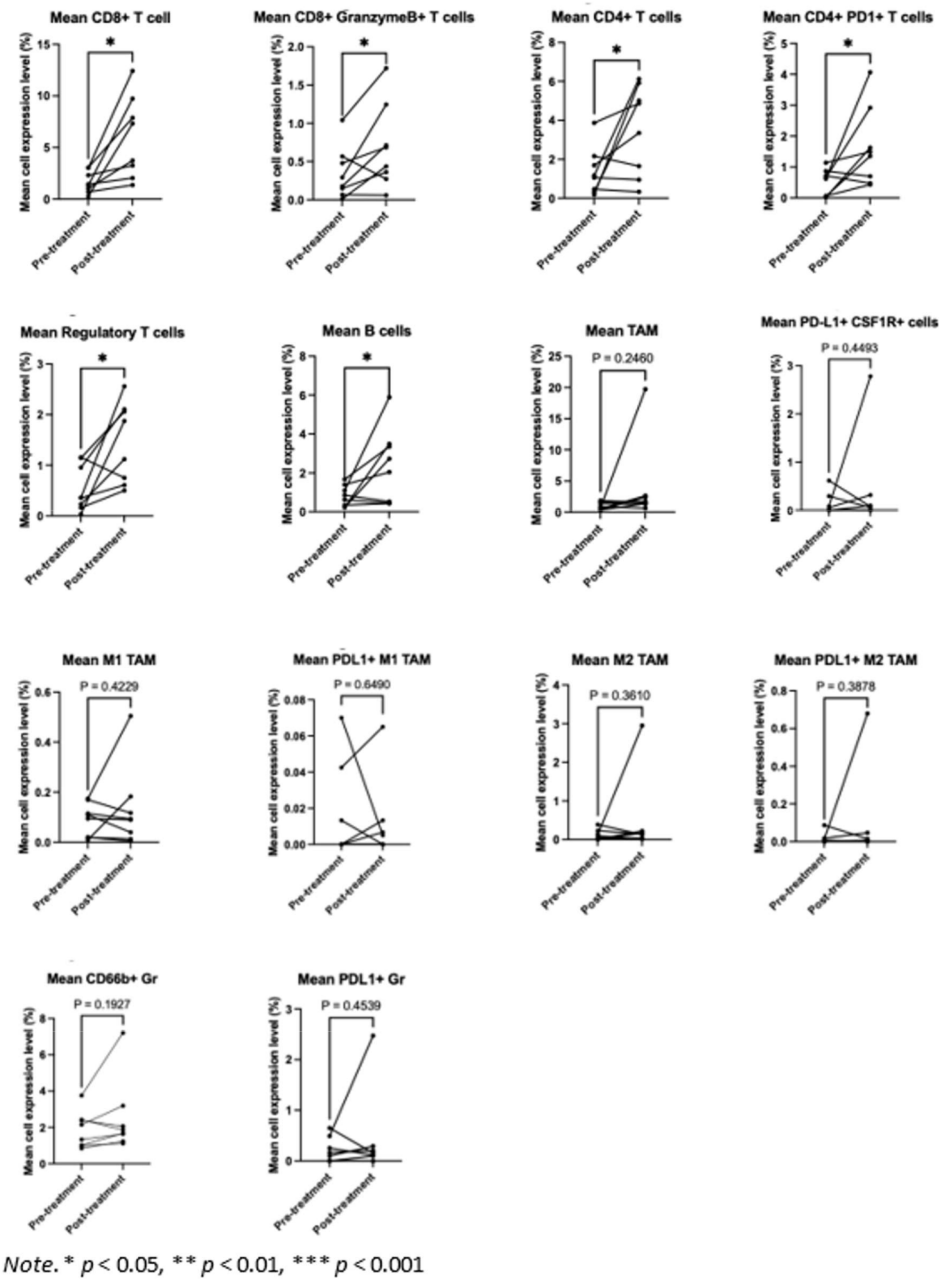
Paired t-test comparing the mean density for each immune cell subtype before and after treatment. * p < 0.05.

CD8+ T cells, CD4+ T cells, CD66b+ granulocytes, and TAMs were the most common cellular subtypes the TME (**Figure 2**). The differences in immune cell subtype densities between pretreatment and posttreatment tumor specimens were assessed using multiplex immunohistochemistry (mIHC) (**Figure 3**). CD8+ T cells were significantly increased following CI (p= 0.0118; **Figure 4**).

The study met its primary immunologic endpoint showing five of eight patients (63%) had a >80% increase in CD8+ T cells and the increase was at least 1.8 times the baseline median absolute deviation (MAD) following CI. Of the five patients who achieved the primary immune endpoint, four showed an increase in Granzyme B+ CD8+ T cells with an increment of >80% and 1.8 times the MAD, indicating cytotoxic CD8+ effector T cell activation.

There was a significant increase in Granzyme B+ CD8+ T cells (p=0.0454). B cells, CD4+ T cells, and Tregs were also significantly increased following the study treatment (p=0.0487, p=0.0487, and p=0.0206, respectively). No significant changes in myeloid cell subtypes including TAMs, PDL1+ macrophages, and granulocytes were observed.

Notably, six of the patients had lower CD66b+ granulocyte levels posttreatment compared to pretreatment, and four of those had a major pathR of grade 0 or 1 (see patients 1–4 in **Figure 2**).

### Efficacy endpoints

**Figure 5:**
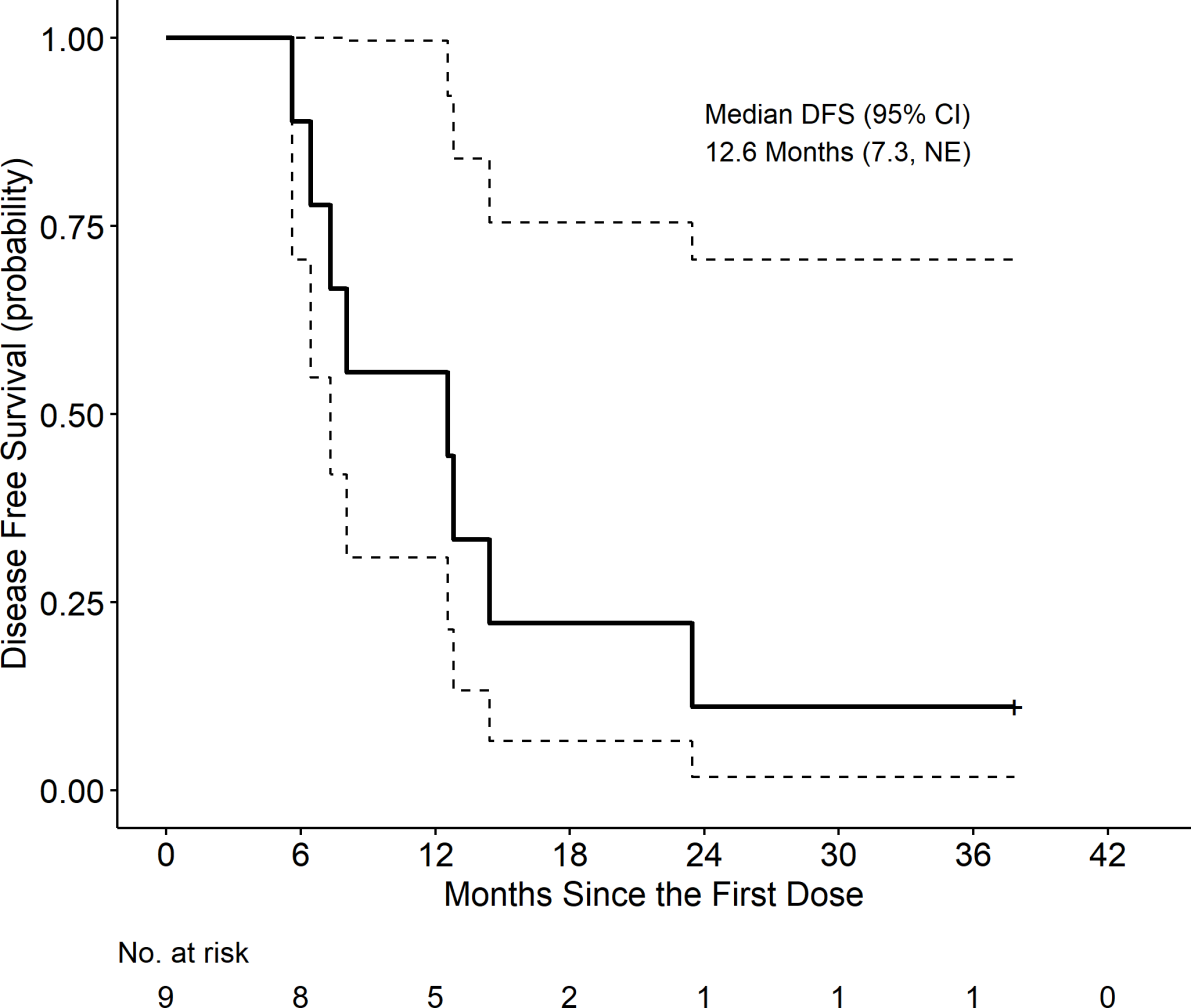
Disease free survival (DFS) is defined as time from first dose to progression/death date, censored at last scan date if alive or death date > 3 months after last scan.

**Figure 6:**
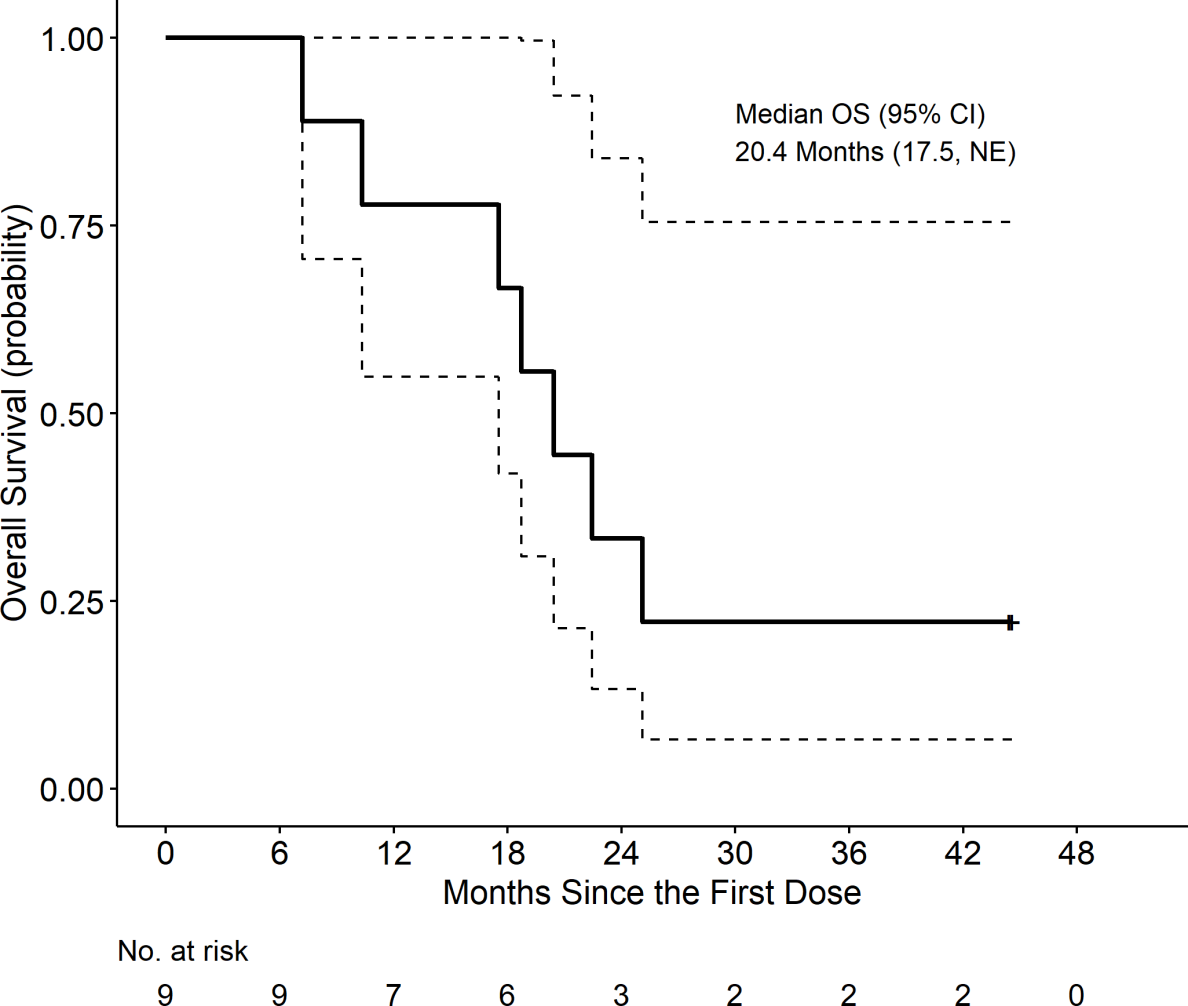
Overall Survival (OS) is defined as time from first dose to death, censored at date of last known alive.

At a median follow-up of 20.4 months, the median disease-free survival (DFS) was 12.6 months (95% CI: 7.3, not estimable (NE)), and the median OS was 20.4 months (95% CI: 17.5, NE). Kaplan-Meir curves are displayed in **Figures 5** and **6**. These includes survival for all patients.

All nine patients (100%) had stable disease per the immune-related Response Evaluation Criteria for Solid Tumors (irRECIST) on CT scan preceding surgery. All nine patients (100%) underwent surgery within two weeks of completion of neoadjuvant CI, with a pathologic response (pathR) rate of 78% (7/9) including one complete pathR (see **Table 4**). The patient who had a complete pathR had stage III disease at diagnosis and disease recurrence 6 months after surgery. The pathological tumor stage following treatment ranged from stage 0 to stage III. Two patients (22%) completed all planned adjuvant CI and remained alive at 2.5 and 3.5 year follow-up. **Supplementary Table 3**includes DFS and OS for all patients.

**Table 4:** Pathologic response using the tumor regression grading system for pancreatic ductal adenocarcinoma after neoadjuvant chemotherapy of the College of American Pathologists.

Pathologic response grade – no. (%)	
0 – No viable tumor cells (complete response)	1 (11%)
1 – Single cells or rare groups of cancer cells (near complete response)	3 (33%)
2 – Residual cancer with evidence tumor regression, but more than single cells or rare groups of cancer cells (partial response)	3 (33%)
3 – Extensive residual cancer with no evident tumor regression (poor or no response)	2 (22%)

## Discussion

In this translational pilot study, we hypothesized targeting M2-like macrophages, regulated by the colony stimulating factor-1 (CSF1) pathway, would be safe and induce an intratumoral immune response in patients with PDA. The study met the primary endpoints and demonstrated that CI with GVAX/CY, IMC-CS4, and Pem is feasible, has a manageable safety profile, and increased the density of activated CD8+ T cells in PDA tumors in patients treated in the perioperative setting. Lower CD66b+ granulocyte levels were correlated with a major pathR in 4 patients.

The safety findings of our study are consistent with previous studies evaluating CSF1R inhibition with ICI, that demonstrated tolerability but notable grade ≥3 toxicities in creatine phosphokinase (CPK) and liver transaminase elevations (33–39). The most common AEs, aside from vaccine injection site reactions, were increased liver transaminases and periorbital edema. Elevated ALT and AST, can be seen in CSF1R inhibition due to decreased clearance due to Kupffer cell inhibition (40). Periorbital edemais considered a class effect of CSF1R inhibitors (41). Our safety profile was more tolerable than previously reported in dual-ICI trials, where about half of the patients experienced treatment-related grade ≥3 AEs (4). Pre-op immunotherapy did not interfere with the feasibility of surgical procedures.

Our study met its primary immunologic endpoint in five out of eight patients. Of the five patients who achieved the primary immune endpoint, four showed an increase in Granzyme B+ CD8+ T cells, indicating cytotoxic CD8+ effector T cell activation. Two studies evaluating combination anti-CSF1R inhibition with ICI have evaluated the immune response comparing paired pre- and post-treatment biopsies in a limited number of patients with metastatic disease (36, 38). In a study combining the anti-CSF1R antibody emactuzumab with the anti-PDL1 antibody atezolizumab, patients without progression were found to have a significantly higher total CD8+ T cell infiltrate present at baseline (36). Baseline TAM densities did not differ between those who progressed and those who did not. When comparing paired biopsies, authors noted a marked decrease in CSF1R+ TAM levels. The median change was comparable in those with or without progressive disease, but was lower in those without progression, suggesting loss of CSF1R may be a mechanism of resistance. A separate study combining AMG 820 and Pem found an increase in PDL1 expression and doubling of CD4+ and CD8+ cell numbers (38). Overall, these findings are in alignment with our study.

In contrast to prior studies, a decrease in TAM levels was not observed in our study. It is possible GM-CSF in the GVAX platform may have contributed to these divergent findings due to the stimulatory effects of GM-CSF on myeloid cell subtypes altering numerical quantification. GM- CSF can drive recruitment, differentiation, and activation of multiple myeloid populations including macrophages (42). Recent studies also suggest potential synergy when using engineered GM-CSF combined with CSF1R inhibition which can reprogram M2-like macrophages toward M1-like antitumor phenotypes (43, 44). The combination of CSF1R blockade with GM-CSF in acute myeloid leukemia models reprogrammed macrophages and disrupted pro-tumoral interactions (42). Further studies addressing the TME changes with and without GM-CSF and paired with clinical endpoints may help elucidate these findings. The observed association between lower CD66b+ granulocyte levels and major pathologic response suggests that CSF1R inhibition may have preferentially targeted neutrophils expressing CSF1R (26). The stability of tumor-associated neutrophil (TAN) levels in patients without major pathological response, suggests a potential resistance mechanism although the exact mechanism remains to be elucidated. Increased Treg density and activation may be a compensatory mechanism of resistance to CSF1R inhibition (27, 31). Other potential mechanisms of resistance may be due to heterogeneity of tumors in patients with PDAC although difficult to address given the limitations of tumor sampling.

Our study enrolled patients after completing standard neoadjuvant therapy. Recent studies examining the effect of neoadjuvant chemotherapy and chemoradiotherapy in the TME have favored a shift to an anti-tumorigenic state with reduction of regulatory T cells and immunosuppressive granulocytes while enhancing proinflammatory cytokine production by tumor-infiltrating T cells (32, 45–47). FOLFIRINOX has been shown to reduce immunosuppressive M2 macrophage density and increase the M1:M2 ratio (47, 48). M1-polarized macrophages localize closer to tumor cells, and this colocalization can correlate with improved pathologic response and survival (47). Neoadjuvant FOLFIRINOX with or without chemoradiation may reduce HLA-A defects, increase CD8+ cell density, decrease T regulatory cell and M2 macrophage density (48). In a randomized SWOG S1505 trial comparing FOLFIRINOX versus gemcitabine/nab-paclitaxel showed that complete/major pathologic response was associated with higher total CD3+ T cell density (49). Thus, it is possible that the chemotherapy and radiation therapy received by the patients prior to enrollment in this study may have altered the TME before they received investigational drugs. It is important to note, previous studies noted immune cells exhibited substantial heterogeneity across patients’ tumors. In contrast, our study used paired biopsies to assess immunological changes within each individual patient by comparing the post immunology sample (surgical specimen) to the baseline sample. The baseline biopsy was obtained after patient had completed SOC chemotherapy +/- chemoradiation. This approach may decrease biases accounted for tumor heterogeneity across different patients and previously received neoadjuvant chemotherapy. Although neoadjuvant chemoimmunotherapy (combined) versus immunotherapy alone has higher rates of treatment-related adverse events across multiple tumor types (50), the safety profile remains acceptable as treatment-related deaths remain infrequent and most immune-related adverse events are manageable with appropriate monitoring (51). We used sequential administration with immunotherapy given after completion of SOC. This approach has demonstrated safety with no dose-limiting toxicities and no surgical delays in several small studies of PDA (22, 52, 53).

Our study was not powered to test clinical endpoints and the small sample size precludes objective assessment of clinical efficacy endpoints. In our enrolled patients, survival data shows similar DFS and OS data to those of studies utilizing SOC neoadjuvant chemotherapy with or without SBRT (42, 44). A positive note was the long-term survival of two patients who completed the full regimen and demonstrated an immune response. The two patients who completed adjuvant therapy remain alive at 2.5 and 3.5 year follow up. Although the primary tumor TME was altered with our immunotherapy approach, additional work is needed to achieve improved survival outcomes in a larger number of patients.

The limitations of our study include its small size and the lack of a control arm. It is important to note that this was a pilot study with the main objectives of evaluating the CI’s safety in patients and biological effect on the TME. This approach, including pre- and posttreatment biopsies, provides the opportunity for a comprehensive immune analysis of treatment-related changes in the heterogeneous TME and evaluation of the scientific merits of our CI before performing larger efficacy studies. Additional limitations include potential spatial and temporal biases introduced by comparing pretreatment EUS core biopsies with posttreatment surgical specimens. Core biopsies capture only a small fraction of the tumor, potentially missing critical heterogeneous regions.

## Conclusion

In summary, the combination of neoadjuvant and adjuvant GVAX/CY, IMC-CS4, and Pem had a manageable safety profile, increased intratumoral activated and cytotoxic T-cells, and demonstrated promising TME modulation in resectable and BR PDA patients, warranting further study. We observed an impressive immunological response to CI. While the two patients who had long term survival demonstrated an immune response, not all who had an immune response derived clinical benefit. Additional work is needed to further understand PDA tumor biology and identify ways to effectively alter its TME, decreasing resistance to ICI.

## Data Availability

The original contributions presented in the study are included in the article/**Supplementary Material**. Further inquiries can be directed to the corresponding author/s.
