# Supplementary material for: Safety and immunologic impact of neoadjuvant/adjuvant GVAX, cyclophosphamide, pembrolizumab, and anti-CSF1R agent IMC-CS4 in pancreatic adenocarcinoma
Source: Front Immunol. 2026 Mar 9;17:1715761. doi: 10.3389/fimmu.2026.1715761 (PMC13006681; doi:10.3389/fimmu.2026.1715761)
Supplement: Supplementary file 1 [file DataSheet1.pdf]

TITLE: A Pilot Study of a GVAX Pancreas Vaccine (with Cyclophosphamide) in Combination with a PD-1 Blockade Antibody (Pembrolizumab) and a Macrophage Targeting Agent (CSF1R inhibitor) for the Treatment of Patients with Borderline Resectable Adenocarcinoma of the Pancreas

Johns Hopkins #: J1766/IRB00130267

Merck Protocol #: MK-3475-536

Principal Investigator:
Ana De Jesus-Acosta, M.D.
1650 Orleans St, Rm 409
Baltimore, MD 21287
Office: 443-287-0411 / Fax: 410-614-8216
adejesu1@jhmi.edu

Translational Principal Investigators:
Lei Zheng, MD, Ph.D.
1650 Orleans St, Rm 488
Baltimore, MD 21287
Office: 410-502-6241 / Fax: 410-614-8216
lzheng6@jhmi.edu

Regulatory Specialist:
Christina Rodriguez
Skip Viragh Outpatient Cancer Building, Rm#8134
201 N. Broadway
Baltimore, MD 21287
Phone: 410-614-6189
Fax: 410-955-6801
Crodri38@jhmi.edu

Statistician:
Hao Wang, Ph.D.
550 Bldg, Room 1101
Baltimore, MD 21287
(410)-614-3426
hwang76@jhmi.edu

Lead Study Coordinator:
Davis Liu
Skip Viragh Outpatient Cancer Building 8200-36, Box 11
201 N. Broadway
Baltimore, MD 21287
Phone: 410-955-6632/Fax: 410-955-6487
mliu5@jhmi.edu

Lead Research Nurse: Carol Judkins, RN
201 N Broadway, 8152 Box 11
Baltimore, MD 21287
410-614-5241/410-502-2730
judkica@jhmi.edu

Program Manager: Katrina Purtell, RN
201 N. Broadway 8150 Box 11
Baltimore, MD 21287
410-502-7448/Fax: 410-502-5860
Email: kalino1@jhmi.edu

Merck Supplied Agent: Pembrolizumab (KEYTRUDA®, MK-3475)

Eli Lilly Supplied Agent: IMC-CS4 (LY3022855)

JHU Supplied Agent: GVAX pancreas vaccine
(Panc 10.05 pcDNA-1/GM-Neo, Panc 6.03 pcDNA-1/GM-Neo)

Commercial Agent: Cyclophosphamide

IND: BB IND16793

IND Sponsor: Elizabeth Jaffee, MD
1650 Orleans St, Room 4M07
Baltimore, MD 21287
Office: 410-955-2957 / Fax: 410-614-8216
ejaffee@jhmi.edu

Protocol Type / Version # / Version Date: Original/ Version 1 / April 18, 2017
Original/ Version 1.1 / May 31, 2017
Amendment 1/ Version 2 / March 6, 2018
Amendment 2/Version 3 / October 25, 2018
Amendment 3/Version 4 / January 3, 2019
Amendment 3/Version 4.1/ February 1, 2019
Amendment 4/Version 5.0/August 8, 2019
Amendment 5/Version 6.0/June 17, 2020
Amendment 6/Version 7.0/July 14, 2020
Amendment 7/Version 8.0/August 10, 2020
Amendment 8/Version 9.0/December 7, 2020

SCHEMA

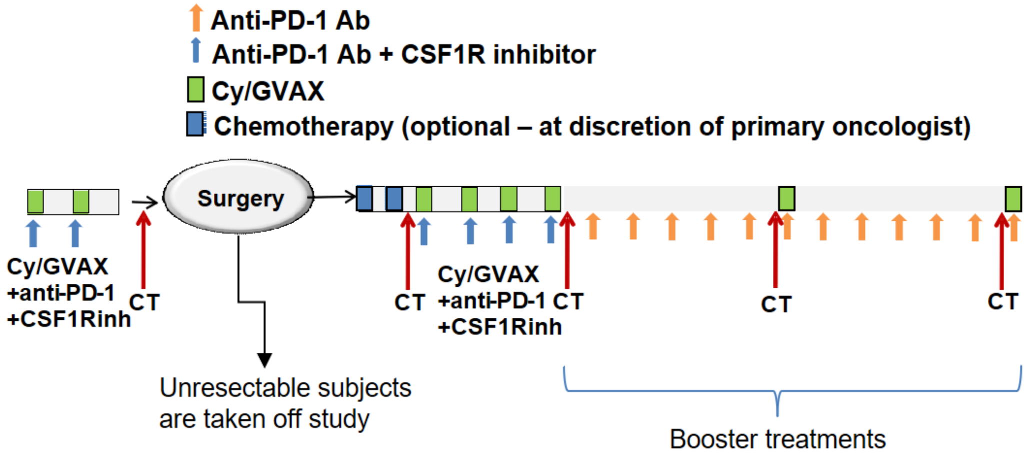
Anti-PD-1 Ab
Anti-PD-1 Ab + CSF1R inhibitor
Cy/GVAX
Chemotherapy (optional – at discretion of primary oncologist)
Surgery
Cy/GVAX
+anti-PD-1
+CSF1Rinh
CT
CT
Cy/GVAX
+anti-PD-1
+CSF1Rinh
CT
CT
CT
Unresectable subjects
are taken off study
Booster treatments

TABLE OF CONTENTS

SCHEMA ... 2

1. OBJECTIVES ... 5
1.1 Primary Objectives ... 5
1.2 Secondary Objectives ... 5
1.3 Exploratory Objectives ... 5
1.4 Primary Endpoint ... 6
1.5 Secondary Endpoints ... 7
1.6 Study Design ... 7

2. BACKGROUND ... 10
2.1 Disease Type ... 10
2.2 Management of Borderline and Resectable Pancreatic Cancer (BRPC) ... 10
2.3 Vaccine Therapy (GVAX) ... 11
2.4 anti-PD-1/PD-L1 blockade [Pembrolizumab (MK-3475 or Keytruda)] ... 12
2.5 CSF-1R Inhibition (IMC-CS4) ... 14
2.6 Preclinical and Clinical Trial Data ... 14
2.7 Rationale ... 14

3. PATIENT SELECTION ... 15
3.1 Eligibility Criteria for enrollment into study and to receive neoadjuvant study treatment ... 15
3.2 Eligibility Criteria to receive adjuvant study treatment ... 17
3.3 Eligibility Criteria to receive study booster therapy ... 18
3.4 Exclusion Criteria for enrollment into study and for the administration of adjuvant/booster immunotherapy: ... 19
3.5 Inclusion of Women and Minorities ... 21

4. TREATMENT PLAN ... 21
4.1 Agent Administration ... 21
4.2 General Concomitant Medication and Supportive Care Guidelines ... 24
4.3 Definition of an Overdose for this Protocol ... 31
4.4 Contraception, Use in Pregnancy, Use in Nursing ... 32
4.5 Unacceptable Toxicities ... 35
4.6 Criteria for Removal from Study Treatment ... 36
4.7 Off Study/Safety Follow-up Visit ... 37
4.8 Duration of Follow Up ... 37

5. DOSING DELAYS/DOSE MODIFICATIONS ... 38
5.1 Dose Modifications ... 38
5.2 Dosing Delays ... 39

6. ADVERSE EVENTS: LIST AND REPORTING REQUIREMENTS ... 44
6.1 Definitions ... 44
6.2 Relationship ... 46
6.3 Expectedness ... 47
6.4 Handling of Expedited Safety Reports ... 47
6.5 Reporting ... 48

6.6 Special Considerations for Adverse Events that Occur During the Surgery and 50

7. PHARMACEUTICAL INFORMATION 54
7.1 Cyclophosphamide (Cytoxan®, CY) 54
7.2 GVAX Pancreas Vaccine 56
7.3 Pembrolizumab (KEYTRUDA®, MK-3475) 58
7.4 IMC-CS4/ LY3022855 61

8. CORRELATIVE/SPECIAL STUDIES 64
8.1 Tumor Tissue and Whole Blood for Peripheral Blood Mononuclear Cells (PBMC) and Circulating Tumor Cells (CTCs) Studies 64
8.2 Serum and Plasma Marker Studies 66
8.3 Diagnostic Tissue Samples 66
8.4 Genomic Analysis 66

9. STUDY SCHEDULE 67
9.1 Study Schedule Prior to Surgery 67
9.2 Study Schedule After Surgery/ Adjuvant Chemotherapy 71
9.3 Study Schedule for Booster Study Immunotherapy 75

10. MEASUREMENT OF EFFECT 78
10.1 Antitumor Effect – Solid Tumors 78

11. DATA REPORTING / REGULATORY REQUIREMENTS 79
11.1 Data Management 79
11.2 Safety Meetings 80
11.3 Monitoring 80

12. STATISTICAL CONSIDERATIONS 81
12.1 Sample Size/Accrual Rate 81
12.2 Early Stopping Boundaries for Toxicity 82
12.3 Analysis of Primary Safety Endpoints 83
12.4 Analysis of Primary Immunology Endpoints 85
12.5 Analysis of Secondary and Exploratory Endpoints 85
12.6 Biomarker Analysis 86

REFERENCES 88

APPENDIX A: Performance Status Criteria 91

APPENDIX B: Adverse Event of Clinical Interest (ECI) Reporting Form 92

APPENDIX C: SAE Reporting Form 95

APPENDIX D: Immune Related Response Criteria 98

1. OBJECTIVES

1.1 Primary Objectives

1.1.1 To assess the intratumoral immune response in the primary tumor after neoadjuvant administration of combination immunotherapy including cyclophosphamide (CY), GVAX pancreas vaccine, pembrolizumab, and CSF1R inhibitor IMC-CS4.

1.1.2 To determine the safety of the combination of GVAX Pancreas Vaccine (with CY), pembrolizumab, and a macrophage targeting agent (CSF1R inhibitor IMC-CS4) in patients with resectable or borderline resectable pancreatic cancer (BRPC) prior to and following surgery.

1.2 Secondary Objectives

1.2.1 To determine the overall survival (OS) of patients with resectable or borderline resectable pancreatic cancer treated with standard neoadjuvant chemotherapy (with or without radiation) who subsequently receive CY/GVAX/pembrolizumab/IMC-CS4.

1.2.2 To determine the disease free survival (DFS; for those who undergo curative resection) of subjects with BRPC treated with neoadjuvant chemotherapy (with or without radiation) who subsequently receive CY/GVAX/pembrolizumab/IMC-CS4.

1.2.3 To determine the objective response rate following the study immunotherapy prior to surgical resection. Imaging studies to assess response will be evaluated using immune-related RECIST criteria (irRC).

1.2.4 To assess the surgical resectability rate in subjects treated with standard neoadjuvant chemotherapy (with or without radiation) who subsequently receive CY/GVAX/pembrolizumab/IMC-CS4.

1.2.5 To assess the pathologic response rate of patients with BRP and resectable pancreas cancer treated with neoadjuvant chemotherapy (with or without radiation) who subsequently receive CY/GVAX/pembrolizumab/IMC-CS4.

1.3 Exploratory Objectives

1.3.1 To evaluate the effects of the combination of pembrolizumab, CY/GVAX, and IMC-CS4 upon the activation and expansion of T effector cells (Teffs) infiltrating into the tumor microenvironment (TME) compared to data from

ongoing JHU trials evaluating the TME in patients treated with 1) chemotherapy and radiation alone or 2) immunotherapy alone

1.3.2 To assess the response to CSF1R inhibition to measure serum levels of $CD14^{dim}CD16^{+}$ monocyte subsets and using ELISA to measure serum CSF1 levels and whether these changes in levels correlate with clinical response to CSF1R inhibition

1.3.3 To monitor the IHC (immunohistochemistry) of immune parameters relevant to the activation of PD-L1/PD-1 associated immunosuppressive pathways, vaccine induced immune regulatory signatures, and peripheral and intratumoral antigen specific T cell responses after treatment with GVAX, pembrolizumab, CY, IMC-CS4, and standard multimodality treatments

1.3.4 To evaluate the effects of the combination of an anti-PD1 antibody, CY/GVAX, and CSF1R inhibitor on PD-L1/PD-1, the PD-L1/PD-1 associated pathways, M1 vs. M2 tumor-associated macrophages (TAMs), macrophage markers (including CD40, 4-IBBL, OX40L), Th1 vs. Th2 vs. Th17, and Teff vs. Treg cells

1.3.5 To assess tumor tissue for molecular determinants of response, progression, and disease stability using next generation sequencing technology

1.3.6 To assess tumor burden dynamics using both standard protein biomarkers such as CA19-9 and other exploratory circulating biomarkers in serial collections of sera and plasma at baseline and throughout treatment

1.3.7 To assess baseline characteristics of the subjects enrolled and to correlate these molecular and clinical/pathologic criteria with treatment response and toxicity. DNA will be extracted from whole blood and used to evaluate for any germline mutations which may correlate with response or toxicity.

1.3.8 To collect peripheral blood mononuclear cells to explore the association of PD-1 positivity, and lymphocyte activation markers with clinical responses

1.4 Primary Endpoint

1.4.1 Immune endpoints

- To evaluate intratumoral immune parameters including but not limited to the following: tumor-associated macrophages (TAM), effector T cell density, and vaccine-induced lymphoid aggregates in patients who received neoadjuvant combination immunotherapy and underwent surgery.

1.4.2 Safety endpoints

- Number of grade 3 and higher toxicities according to National Cancer Institute Common Toxicity Criteria for Adverse Events (NCI CTCAE; Version 4.0) that occur after Cycle 1, day 1
- Incidence, nature and severity of all adverse events that occur on or after Cycle 1, Day 1

1.5 Secondary Endpoints

1.5.1 Efficacy endpoints

- Disease free survival (DFS) from the time of cycle 1, day 1 of immunotherapy until first documented evidence of disease recurrence or death.
- Objective response rate to combination immunotherapy with imaging studies.
- OS from the time of cycle 1, day 1 of immunotherapy until death from any cause
- Surgical resectability after neoadjuvant therapy as determined by the number of patients able to undergo surgical resection.
- Pathologic response as determined by surgical margins status at the time of surgery and response to neoadjuvant treatment per surgical specimen assessment.

1.5.2 Exploratory endpoints

- IHC, flow cytometry, quantitative PCR assays and microarray analysis compared between pre- and post-treatment tumor specimens to evaluate
  - Immune parameters relevant to the PD-L1/PD-1 pathway
  - CSF-1 serum levels and levels of $CD14^{dim}CD16^{+}$ monocyte subsets
  - Densities and distribution of T-cells in TME
  - Macrophage markers
  - Expression of T helper cell differentiation markers
- Cytokine/chemokine assays to assess changes in intratumoral inflammatory markers
- Whole exome sequencing to identify neoantigens therapy by comparing tumor-specific non-synonymous mutations before and after treatment
- Peripheral antigen specific T-cell responses
- Intratumoral antigen specific T-cell responses
- Biomarker changes (including standard protein biomarkers such as CA 19-9) and correlation to evaluate for prognostic or predictive factors
- To assess correlation between immune parameters and clinical outcomes using univariate and multivariate Cox regression models

1.6 Study Design

This is a single center, open label, pilot study to evaluate the safety, immune effect and early signs of clinical activity of a combination of multiple immunotherapy agents in a

neoadjuvant/adjuvant setting in patients with BRPC or resectable pancreatic cancer. Patients with BRPC or resectable pancreas cancer will receive the combination of CY/GVAX with pembrolizumab and IMC-CS4 following completion of standard neoadjuvant chemotherapy (with or without radiation). Immunotherapy agents will be given prior to and after surgical resection.

We will enroll subjects with initial diagnosis of BRPC or resectable pancreas cancer based upon criteria from NCCN Guidelines as determined by review of imaging and pathology with a multidisciplinary team (MDT). Eligible patients must have received neoadjuvant chemotherapy (with or without radiation) and must be considered a candidate for curative resection.

Consented and eligible patients will undergo EUS-guided pretreatment research core biopsy of the pancreatic tumor and research blood draws to evaluate for circulating tumor cells and other immune/inflammatory markers within 14 days prior to the first immunotherapy treatment. Participants will receive their first dose of combination immunotherapy, consisting of cyclophosphamide (CY) 200 mg/m^2 given intravenously (IV) and pembrolizumab 200 mg IV on day 1, IMC-CS4 on day 1, 8, 15, and GVAX administered as six intradermal injections on day 2, repeated every 21 days as one cycle. Patients will receive a total of two cycles of immunotherapy prior to surgical resection.

The study will comprise of <u>two parts</u> for assessment of the safety of IMC-CS4 dose escalation.

- Part One of the study will enroll three patients who will receive IMC-CS4 at Dose Level 1 (DL1). They will be monitored for unacceptable toxicities (as defined in **Section 4.5**) and potential immune-related adverse events through the first two cycles of immunotherapy and subsequent surgical resection. If there is ≤ 1 patient during Part One who develops an unacceptable toxicity, the study will progress to Part Two. If two or more patients develop an unacceptable toxicity, the study will be paused and the protocol will be re-evaluated and discussed with the IND Sponsor.

- Part Two of the study will enroll the remaining nine patients, who will receive IMC-CS4 at Dose Level 2 (DL2), which is the IMC-CS4 dose that is expected to be the recommended phase 2 dose from JSCA (NCT01346358). DL2 will not be initiated until a minimum of 6 patients have been treated with 100 mg flat dosing cohort in JSCA for at least one cycle and ≤ 1 patient experience a dose-limiting toxicity in JSCA. During Part Two, we will follow the Bayesian stopping guideline to monitor unacceptable toxicities as outlined in **Section 12.2** and **Table 9.** If a subject develops an unacceptable toxicity on DL2, treatment for that subject will be discontinued. Subsequently enrolled patients in Part 2 will still be initiated on DL2. If it becomes evident that the proportion of patients who experience an

unacceptable toxicity as defined in section 4.5 convincingly exceeds 30% during Part Two of the study, the study will be halted for a safety consultation with IND Sponsor. The stopping rule will hold enrollment if the posterior probability of toxicity risk exceeding 30% is 75% or higher.

Seven to fourteen days following the 2 cycles of immunotherapy, patients will undergo restaging imaging to evaluate response to immunotherapy. After discussion at MDC and/or pancreatic tumor board if patients have stable disease or further response as compared to baseline will be candidates for surgical resection. Standard surgical procedure will be determined by the operating surgeon. Pancreatectomy is considered standard of care for pancreatic cancer and therefore will not be part of the protocol.

An internal and external real-time monitoring plan will be put in place to ensure that toxicities are captured and evaluated in a timely, appropriate, and non-biased manner. Patients will be monitored at regular intervals for a broad range of toxicities, including enhanced inflammatory related complications prior to surgery, delayed surgery related healing, dermatologic toxicity at the vaccine site, GM-CSF-related toxicity, CY-related toxicity, and immune-related adverse events (irAEs).

During the four to eight weeks following surgical resection, patients have the option of being treated with adjuvant chemotherapy at the discretion of the primary oncologist. While the patient is receiving the adjuvant chemotherapy, she/he will not receive any immunotherapy.

All patients (whether or not they received adjuvant chemotherapy) who underwent surgical resection and have no evidence of recurrence/progression will receive four additional cycles of adjuvant combination immunotherapy as long as they do not fulfill the exclusion criteria below.

The following will be the exclusion criteria to continue additional adjuvant study immunotherapy:

1. BRPC patients: More than 90 days have elapsed since surgery (in the absence of adjuvant chemotherapy). The patient will be considered off study. More than 180 days have elapsed since the surgery (for those patients who receive adjuvant chemotherapy). Patients with resectable disease will be allowed more than 180 days if a longer course of adjuvant chemotherapy is required.
2. Patients who were found to have unresectable or metastatic disease intraoperatively
3. Patients who develop disease progression at any point in the study. These patients will be monitored for the safety endpoint but will not continue to receive further immunotherapy. The patients will be offered an optional biopsy for research purposes.

Following the 4 adjuvant study immunotherapy cycles, as long as the patient remains disease free and more than 90 days have not elapsed since the last immunotherapy

treatment, the patients will receive 12 booster cycles of pembrolizumab every 3 weeks, and 2 booster cycles of CY/GVAX given every 18 weeks. The first booster CY/GVAX will be given when receiving 6th booster dose of pembrolizumab (cycle 12), and the second booster CY/GVAX will be given when receiving 12th booster dose of pembrolizumab (cycle 18).

Subjects that are required to stop treatment with pembrolizumab and/or IMC-CS4 due to toxicity may continue to receive CY/GVAX pancreas vaccine until they develop disease progression or unacceptable toxicities.

After patients have completed all planned therapy, they will undergo standard of care evaluations through their primary oncologist, consisting of history and physical, CT of the chest/abdomen/pelvis (or MRI abdomen/pelvis and non-contrast CT chest if do not tolerate contrast) every three to six months or as indicated to evaluate for local progression and metastatic disease. The study will collect the follow-up information from primary oncologist.

Per the FDA requirement for patients treated with genetically modified products, all research participants will be encouraged to enroll in a long-term follow-up protocol, following their completion of all interventional studies. These patients will be followed for disease progression, survival and potential long term toxicity of gene therapy in an existing protocol entitled "Long term follow-up of patients who received lethally irradiated allogeneic pancreatic tumor cells transfected with the GM-CSF gene (IRB # 02-10-14-03, SKCCC J0248)".

2. BACKGROUND

2.1 Disease Type

Over 46,000 people were diagnosed with pancreatic ductal adenocarcinoma (PDAC) in the United States in 2014, with only a 6.7% expected 5 year survival.[1, 2] Surgical resection is the only potentially curative modality of treatment. At the time of diagnosis, most patients present with unresectable disease and less than 20% of patients are considered candidates for surgery. Following resection, the majority of patients with initially resectable disease develop recurrence.[3] There are a subgroup of patients with "borderline resectable" pancreatic cancer (BRPC) in which the tumor is technically resectable but has increased risk of positive margins, particularly if no neoadjuvant therapy is administered.[4]

2.2 Management of Borderline and Resectable Pancreatic Cancer (BRPC)

To increase the probability of negative margins, patients with borderline resectable disease undergo preoperative regimens including chemotherapy and radiation to help downstage the primary tumor prior to surgical resection. Our analysis of BRPC patients who received neoadjuvant chemotherapy at the Johns Hopkins Hospital showed a

resectability rate of 48.8%. Despite this, the median overall survival of the BRPC patients who underwent surgical resection was only 22.9 months.[5] This is shorter than the 31-34 months reported survival of patients with resectable disease at diagnosis who receive neoadjuvant chemoradiation followed by surgical resection.[6, 7] The results suggest that there is a need for better neoadjuvant therapy to improve the resectability of BRPC as well as better postoperative therapy to prolong overall survival. Therefore, additional systemic chemotherapy and immunotherapy are needed to help prevent local and systemic progression.

In patients with resectable disease standard of care has traditionally been upfront surgery followed by adjuvant therapy. Although multiple contemporary studies have clearly established the benefit of adjuvant chemotherapy, its administration is not uniform across all resected patients. It is estimated that up to 50% of patients do not receive adjuvant therapy due to variety of post-operative circumstances. Additionally, post-operative recovery delays time to adjuvant therapy. As such, many any centers are now using chemotherapy in the neoadjuvant setting as standard of care for patients with resectable disease. Similarly, NCCN guidelines suggest clinical trial participation.

2.3 Vaccine Therapy (GVAX)

Immunotherapy has been investigated as a potential concurrent treatment mechanism by stimulating the immune system to recognize a diverse array of tumor antigens. Vaccine therapy has been successful in stimulating immunologic killing of tumor cells via alternate mechanisms than that of chemotherapy or radiation. The GVAX pancreas vaccine is a combination of two irradiated, granulocyte-macrophage colony-stimulating factor (GM-CSF) secreting allogeneic pancreatic tumor cell lines.[8, 9] GM-CSF is an important cytokine in inducing the growth and differentiation of dendritic cells, which act as antigen-presenting cells in encoding tumor antigens. Autologous GM-CSF secreting vaccines have shown immune activation in 10-40% of treated subjects in melanoma, renal cell, prostate, lung, breast, and pancreatic cancers.[10-12] However, autologous vaccines are often technically difficult to produce, and so allogeneic GM-CSF vaccines have been tried and shown to be safe, tolerable, and prime even HLA-incompatible CD8+ effector T cells to tumor beds.[9, 13]

The initial Phase I study of GVAX in PDAC was a dose escalation trial of adjuvant vaccination in 14 patients with Stage 2 or 3 disease.[9] Subjects received vaccination 8 weeks following resection, then adjuvant chemoradiation, then three additional monthly vaccinations. Treatment was well tolerated, with toxicities limited to grade 1 and 2 local reactions at the vaccine site, and self-limited systemic rashes, with DTH responses observed in 1 of 3 patients receiving 10^8 and in 2 of 5 patients receiving 5 x 10^8 vaccine cells. Analysis of 60 patients in a follow-up phase II trial of adjuvant GVAX who subsequently received a total of 5 vaccinations in addition to chemoradiation showed 86% one-year survival and 61% two-year survival.[14] The vaccine was again well tolerated, with transient local site reactions, mild eosinophilia, rashes, flu-like symptoms (low grade fever, chills, malaise, arthralgias, myalgias, and

fatigue).

Further studies have shown the boosted effect of GVAX by its combination with low dose cyclophosphamide (Cytoxan, CY) as an immune modulator. Tumors have several mechanisms for evading immune surveillance, including the development of tolerance with immunosuppressive regulatory T cells (Treg).[15-18] In murine breast cancer models treatment with CY prior to vaccination showed an enhancing effect with suppression of Treg and increase in effector T cells (Teff).[19] In a phase II trial of treatment for Stage IV pancreatic cancer patients, the combination of CY/GVAX was noted to be safe and well tolerated in gemcitabine resistant metastatic pancreatic cancer, with grade 3/4 treatment related events in only one of 30 subjects[20] Median survival was 2.3 months versus 4.7 months in subjects receiving GVAX without and with CY, respectively. Pathologic evaluation revealed that there was a trend toward prolonged progression-free survival in subjects who had persistent mesothelin-specific T cell responses with therapy.

A study of GVAX given both neo-adjuvantly and adjuvantly was performed recently, either alone or with immune modulating doses of cyclophosphamide (Cytoxan, Cy) to deplete regulatory T cells (Tregs).[21] Pathological examination of PDA tumor tissue resected just two weeks following a single neoadjuvant dose of GVAX identified the formation of novel vaccine-induced, immunologically active, tertiary lymphoid aggregates, organized lymph node-like structures that are not observed in tumor tissue resected from unvaccinated patients. However, we also found that activated T cells secrete interferon-γ, which in turn upregulates the PD-1/PD-L1 pathway.[21, 22] These data support an emerging concept that vaccines are required to induce a T cell response that is capable of infiltrating the TME. However, vaccination is just the first step toward establishing an effective antitumor immune response, converting the PDA TME into an environment similar to what is observed in melanomas exhibiting infiltrating but immunosuppressed T cells prior to immunotherapy treatment. Thus, we hypothesize that treatment with GVAX primes the PDA TME for anti-PD-1/PD-L1-targeted therapy. Supporting this hypothesis, we showed in a preclinical model of PDA that combining anti-PD-1 and anti-PD-L1 antibodies with GVAX+Cy enhances the infiltration of effector T cells into PDA tumors as well as the cure rate in PDA tumor-bearing mice.[22]

2.4 anti-PD-1/PD-L1 blockade [Pembrolizumab (MK-3475 or Keytruda)]

The importance of intact immune surveillance in controlling outgrowth of neoplastic transformation has been known for decades.[23] Accumulating evidence shows a correlation between tumor-infiltrating lymphocytes (TILs) in cancer tissue and favorable prognosis in various malignancies. In particular, the presence of $CD8^+$ T-cells and the ratio of $CD8^+$ effector T-cells / $FoxP3^+$ regulatory T-cells seems to correlate with improved prognosis and long-term survival in solid malignancies such as ovarian, colorectal and pancreatic cancer, hepatocellular carcinoma, malignant MEL

and RCC. TILs can be expanded *ex vivo* and re-infused, inducing durable objective tumor responses in cancers such as melanoma.[24, 25]

The PD-1 receptor-ligand interaction is a major pathway hijacked by tumors to suppress immune control. The normal function of PD-1, expressed on the cell surface of activated T-cells under healthy conditions, is to down-modulate unwanted or excessive immune responses, including autoimmune reactions. The ligands for PD-1 (PD-L1 and PD-L2) are constitutively expressed or can be induced in various tumors.[26-29] Binding of either PD-1 ligand to PD-1 inhibits T-cell activation triggered through the T-cell receptor. PD-L1 is expressed at low levels on various non-hematopoietic tissues, most notably on vascular endothelium, whereas PD-L2 protein is only detectably expressed on antigen-presenting cells found in lymphoid tissue or chronic inflammatory environments. PD-L2 is thought to control immune T-cell activation in lymphoid organs, whereas PD-L1 serves to dampen unwarranted T-cell function in peripheral tissues.[30]   Although healthy organs express little (if any) PD-L1, a variety of cancers were demonstrated to express abundant levels of this T-cell inhibitor. High expression of PD-L1 on tumor cells (and to a lesser extent of PD-L2) has been found to correlate with poor prognosis and survival in various cancer types, including renal cell carcinoma (RCC),[31] pancreatic carcinoma,[32] hepatocellular carcinoma,[33] ovarian carcinoma.[34] Furthermore, PD-1 has been suggested to regulate tumor-specific T-cell expansion in patients with malignant melanoma.[35] The observed correlation of clinical prognosis with PD-L1 expression in multiple cancers suggests that the PD-1/PD-L1 pathway plays a critical role in tumor immune evasion and should be considered as an attractive target for therapeutic intervention.

We showed in a preclinical model of PDA that combining anti-PD-1 and anti-PD-L1 antibodies with GVAX+Cy enhances the infiltration of effector T cells into PDA tumors as well as the cure rate in PDA tumor-bearing mice[22], helping support the hypothesis that GVAX primes the PDA TME for anti-PD-1/PD/L1-targeted therapy.

Pembrolizumab (previously known as SCH 900475, MK-3475) is a potent and highly-selective humanized mAb of the IgG4/kappa isotype designed to directly block the interaction between PD-1 and its ligands, PD-L1 and PD-L2. Pembrolizumab contains the S228P stabilizing mutation and has no antibody-dependent cell-mediated cytotoxicity (ADCC) or complement-dependent cytotoxicity (CDC) activity. Pembrolizumab strongly enhances T lymphocyte immune responses in cultured blood cells from healthy human donors, cancer patients, and primates. In T- cell activation assays using human donor blood cells, the EC50 was in the range of 0.1 to 0.3 nM. Pembrolizumab also modulates the level of interleukin-2 (IL-2), tumor necrosis factor alpha (TNFα), interferon gamma (IFNγ), and other cytokines. The antibody potentiates existing immune responses only in the presence of antigen and does not nonspecifically activate T- cells.

Pembrolizumab is FDA-approved for unresectable or metastatic melanoma at 2 mg/kg every 3 weeks, and for metastatic non-small cell lung cancer and recurrent or metastatic head and neck squamous cell carcinoma at 200 mg flat dose every 3 weeks.

2.5 CSF-1R Inhibition (IMC-CS4)

Colony-stimulating factor 1 (CSF-1) and its receptor CSF-1R regulate the migration, differentiation and survival of macrophages.[36] In solid tumors, tumor-associated macrophages (TAMs) secrete growth factors which release immunosuppressive cytokines which promote tumor growth and are associated with poor prognosis.[37] CSF-1R inhibition in combination with chemotherapy in preclinical models has been shown to result in increased $CD8^+$ T cells, TAM-derived interleukin-10 (IL-10) production and a reduction in tumor burden.[38] CSF-1R inhibition alone rarely produces a potent anti-tumor response but there is emerging evidence that CSF/CSF-1R blockade can reprogram TAMs and render tumors more susceptible to PD-1 blockade.[39]

Multiple monoclonal antibody and small molecule inhibitors of CSF-1R (CSF-1Ri) have been developed, including Eli Lilly's IMC-CS4 and Plexxikon's PLX339. IMC-CS4 is a human IgG1 monoclonal antibody designed to target the CSF-1R. In vitro, IMC-CS4 triggers depletion of TAMs. The CSF-1R inhibitor PLX3397 is a small molecule tyrosine kinase inhibitor which potently and selectively inhibits FMS, Kit, and Flt3-ITD kinases. It was studied in a phase I study in 26 patients with advanced solid tumors.[40] PLX339 is mostly well tolerated with specific drug-related toxicities including fatigue, anemia, rash, and nausea.

To help decrease risk of toxicities with the combination of GVAX/CY, Pembrolizumab and IMC-CS4, we will start a dose level below the recommended phase 2 dose of the IMC-CS4 phase 1 dose-escalation study (as described in **Section 1.6**). We have significant clinical trial experience with GVAX/CY at our institution and the toxicities of pembrolizumab have been well-characterized, so this will help use elucidate toxicities due to the CSF-1R inhibitor, IMC-CS4.

2.6 Preclinical and Clinical Trial Data

Refer to the Investigator's Brochure [IB] for Preclinical and Clinical Data

2.7 Rationale

Attempts to apply anti-CTLA4 and anti-PD1 agents directly to GI tumors and PDAC have been limited due to their nonspecific immune modulation and diminished reactivity in these tumor types, except in select populations. For example, though anti-PD-1 appears to have minimal activity in patients with colorectal cancer, a subset of colorectal cancer patients with microsatellite instability (MSI) disease, were noted incidentally to have complete response to PD-1 inhibitors.[35] Pathologic review of those more sensitive samples is noted to have increased numbers of neo-antigens (to stimulate immune response) and tumor infiltrating lymphocytes (TILs).[41]

GVAX administration recruits Teffs into the tumor microenvironment (TME), as well as upregulate the immunosuppressive regulatory mechanisms such as Treg infiltration

and increased PD-L1 expression. This provides an opportunity to convert PDAC TME into an environment similar to that observed in melanomas with infiltrating, but immunosuppressed T cells that may be more amenable to checkpoint inhibitors.[21]

The combination of ipilimumab (CTLA-4 inhibitor) with GVAX in advanced pancreatic cancer patients is ongoing, and has shown promise in a phase I study of 30 patients with previously treated PDAC showing median OS 3.6 v 5.7 months and 7 versus 27% 1-yr OS in patients treated with ipilimumab without and with GVAX respectively.[42] However, anti-CTLA4 antibodies may have higher toxicity profiles than PD-1 inhibitors. Additionally, PD-1 ligand is highly expressed in a variety of human tumors, including some pancreatic cancer and thus may be a more specific target.[43] In addition, treatment of tumors with GVAX in preclinical models demonstrate that the increased Teffs leads to secretion of interferon-ɣ, which subsequently upregulates the PD-1/PD-L1 pathway.[21, 22] The synergistic effect has been demonstrated in a preclinical model of PDAC where the combination of anti-PD-1 and anti-PD-L1 antibodies with CY/GVAX leads to an enhanced infiltration of effector T cells into PDAC tumors as well as the cure rate in PDAC tumor-bearing mice.[22] There is also emerging evidence that CSF/CSF-1R blockade can reprogram TAMs and render tumors more susceptible to PD-1 blockade.[39]

This clinical trial will therefore evaluate the combination of CY/GVAX and anti-PD-1 antibody (pembrolizumab), and CSF-1R inhibitor (IMC-CS4) for BRPC or resectable pancreas cancer following neoadjuvant treatment given before and after surgical resection.

3. PATIENT SELECTION

3.1 Eligibility Criteria for enrollment into study and to receive neoadjuvant study treatment

3.1.1 Patients with histologically or cytologically confirmed adenocarcinoma of the pancreas.

3.1.2 A research core biopsy is required within 14 days prior to starting therapy. If a biopsy is attempted and unsuccessful, that patient still may be treated after review and approval of the PI and IND Sponsor.

3.1.3 Patients must have borderline or resectable pancreatic adenocarcinoma at time of diagnosis as defined by NCCN guidelines. Discussion will take place at multidisciplinary pancreatic tumor board.

3.1.4 Patients must not have metastatic disease.

3.1.5 Patients must have measurable disease, defined as at least one lesion that can be accurately measured in at least one dimension (longest diameter to be recorded)

as ≥20 mm with conventional techniques or as ≥10 mm with spiral CT scan. See **Section 10.1.2** for the evaluation of measurable disease.

3.1.6 Patients who received radiation therapy must have received last dose no longer than 28 days prior to enrollment.

3.1.7 Patients must have received last dose of chemotherapy at least 14 days or longer prior to receiving study related immunotherapy.

3.1.8 Age ≥18 years.

3.1.9 ECOG performance status 0-1 (**Appendix A**).

3.1.10 Adequate hematologic, renal, and liver function as defined below:

Lymphocytes	≥ 500 cells/mm^3
Absolute neutrophil count	≥ 1,000 cells/mm^3
Hemoglobin	≥ 9g/dL
Platelets	≥ 100,000 cells/mm^3
Serum creatinine	within normal limits
AST and ALT	≤ 2 x ULN
Total bilirubin	≤ 1.5 x ULN **

**Subjects with Gilbert's Syndrome should have direct bilirubin within normal institutional limits

3.1.11 Female patient of childbearing potential (WOCBP) [defined in **Section 4.4.1**] must have a negative urine or serum pregnancy test. If the urine test is positive or cannot be confirmed as negative, a serum pregnancy test will be required to be shown as negative for the patient to be eligible.

3.1.12 WOCBP (defined in **Section 4.4.1**) must be willing to use an adequate method of contraception as outlined in **Section 4.4.1** - Contraception, starting with Visit 1 through 120 days after the last dose of study therapy.

Note: Complete abstinence is acceptable if this is the usual lifestyle and preferred contraception for the subject.

Male subjects of childbearing potential (**Section 4.4.1**) must agree to use an adequate method of contraception as outlined in **Section 4.4.1** - Contraception, starting with Visit 1 through 120 days after the last dose of study therapy.

Note: Abstinence is acceptable if this is the usual lifestyle and preferred contraception for the subject.

3.1.13 Ability to understand and the willingness to sign a written informed consent document.

3.2 Eligibility Criteria to receive adjuvant study treatment

3.2.1 Patients must have had surgical resection of primary tumor (R0, R1 and R2 resections).

3.2.2 Patients must not have evidence of metastatic disease by imaging or disease progression by imaging as per irRC.

3.2.3 If no adjuvant chemotherapy was given, patient must have had surgery within 90 days prior to planned day 1 of adjuvant study immunotherapy. If adjuvant chemotherapy was given BRPC patients must have had surgery within 180 days of adjuvant study immunotherapy. Patients with resectable disease will be allowed more than 180 days if a longer course of adjuvant chemotherapy is required.

3.2.4 ECOG performance status 0-1 (**Appendix A**).

3.2.5 Adequate hematologic, renal, and liver function as defined below:

Lymphocytes	$\geq$ 500 cells/mm^3
Absolute neutrophil count	$\geq$ 1,000 cells/mm^3
Hemoglobin	$\geq$ 9g/dL
Platelets	$\geq$ 100,000 cells/mm^3
Serum creatinine	within normal limit
AST and ALT	≤ 2 x ULN
Total bilirubin	≤ 1.5 x ULN **

**Subjects with Gilbert's Syndrome should have direct bilirubin within normal institutional limits

3.2.6 Female patient of childbearing potential (WOCBP) [defined in **Section 4.4.1**] must have a negative urine or serum pregnancy test. If the urine test is positive or cannot be confirmed as negative, a serum pregnancy test will be required to be shown as negative for the patient to be eligible.

3.2.7 WOCBP (defined in **Section 4.4.1**) must be willing to use an adequate method of contraception as outlined in **Section 4.4.1** - Contraception, starting with Visit 1 through 120 days after the last dose of study therapy.

Note: Complete abstinence is acceptable if this is the usual lifestyle and preferred contraception for the subject.

Male subjects of childbearing potential (**Section 4.4.1**) must agree to use an adequate method of contraception as outlined in **Section 4.4.1** - Contraception, starting with Visit 1 through 120 days after the last dose of study therapy.

Note: Abstinence is acceptable if this is the usual lifestyle and preferred contraception for the subject.

3.2.8 Ability to understand and the willingness to sign a written informed consent document.

3.3 Eligibility Criteria to receive study booster therapy

3.3.1 Patients must not have metastatic disease by interval study imaging or disease progression by interval study imaging as per irRC done.

3.3.2 ECOG performance status 0-1 (**Appendix A**).

3.3.3 Adequate hematologic, renal, and liver function as defined below:

Lymphocytes	$\geq$ 500 cells/mm^3
Absolute neutrophil count	$\geq$ 1,000 cells/mm^3
Hemoglobin	$\geq$ 9g/dL
Platelets	$\geq$ 100,000 cells/mm^3
Serum creatinine	$\leq$ 1.5 x ULN*
AST and ALT	$\leq$ 2 x ULN
Total bilirubin	$\leq$ 1.5 x ULN**

*If the serum creatinine is > 1.5 x ULN, but the estimated GFR is > 30 mL/min, then the booster therapy can be administered.

**Subjects with Gilbert's Syndrome should have direct bilirubin within normal institutional limits

3.3.4 Female patient of childbearing potential (WOCBP) [defined in **Section 4.4.1**] must have a negative urine or serum pregnancy test. If the urine test is positive or cannot be confirmed as negative, a serum pregnancy test will be required to be shown as negative for the patient to be eligible.

3.3.5 WOCBP (defined in **Section 4.4.1**) must be willing to use an adequate method of contraception as outlined in **Section 4.4.1** - Contraception, starting with Visit 1 through 120 days after the last dose of study therapy.

Note: Complete abstinence is acceptable if this is the usual lifestyle and preferred contraception for the subject.

Male subjects of childbearing potential (**Section 4.4.1**) must agree to use an adequate method of contraception as outlined in **Section 4.4.1** - Contraception, starting with Visit 1 through 120 days after the last dose of study therapy.

Note: Abstinence is acceptable if this is the usual lifestyle and preferred contraception for the subject.

3.3.6 Ability to understand and the willingness to sign a written informed consent document.

3.4 Exclusion Criteria for enrollment into study and for the administration of adjuvant/booster immunotherapy:

3.4.1 Patient is currently participating or has participated in a study of an investigational agent within 28 days of C1D1 of this study or is currently using an investigational device.

3.4.2 Patients who have had surgery 28 days prior to first dose of the study drug excluding minor procedures (dental work, skin biopsy, etc.), celiac plexus block, and biliary stent placement.

3.4.3 Patients must not have received systemic steroids, immunosuppressant medications and anti-neoplastic treatment in the past 14 days (with the exception of steroids utilized for the purpose of premedication for contrast CT).

3.4.4 Patients with a history of prior treatment (outside of this study) with immunotherapy agents (including, but not limited to: IL-2, interferon, anti-PD-1, anti-PD-L1, anti-PD-L2, anti-CD137, anti-OX-40, anti-CD40, or anti-CTLA-4 antibodies).

3.4.5 Patients receiving growth factors including, but not limited to, granulocyte-colony stimulating factor (G-CSF), GM-CSF, erythropoietin, within 14 days of study drug administration. Use of such agents while on study is also prohibited during immunotherapy treatment.

3.4.6 Patients who have received any prophylactic vaccine within 14 days of first dose of study drug or received a live vaccine within 30 days of planned start of study therapy.

3.4.7 Uncontrolled, intercurrent illness including, but not limited to, ongoing or active infection, symptomatic congestive heart failure, unstable angina, cardiac arrhythmia, metastatic cancer, or psychiatric illness/social situations that would limit compliance with study requirements.

3.4.8 History of any autoimmune disease, including any history of inflammatory bowel disease, including ulcerative colitis and Crohn's Disease, as well as

history of symptomatic disease (e.g. rheumatoid arthritis, systemic progressive sclerosis [scleroderma], systemic lupus erythematosus, autoimmune vasculitis [e.g., Wegener's Granulomatosis]); CNS or motor neuropathy considered of autoimmune origin (e.g., Guillain-Barre Syndrome and Myasthenia Gravis, multiple sclerosis). Patients with thyroid disease will be allowed. Autoimmune diagnoses not listed here must be approved by the Principal Investigator.

3.4.9 Presence of any tissue or organ allograft, regardless of need for immunosuppression, including corneal allograft. Patients with a history of allogeneic hematopoeitic stem cell transplant will be excluded.

3.4.10 Has history of (non-infectious) pneumonitis that required steroids, history or evidence of interstitial lung disease or active, non-infectious pneumonitis.

3.4.11 Has a pulse oximetry < 92% on room air.

3.4.12 Evidence of ascites on imaging at study enrollment (allowed post-surgery during administration of adjuvant and booster therapy).

3.4.13 Requires the use of home oxygen.

3.4.14 Has a known history of Human Immunodeficiency Virus (HIV) (HIV1/2 antibodies), hepatitis B, or hepatitis C infection (patients who are hepatitis C antibody positive may be enrolled if they are confirmed with negative viral load at screening).

3.4.15 Any concurrent malignancy or myeloproliferative disorder whose natural history or treatment has the potential to interfere with safety or efficacy assessment of this study's investigational drug. Patients with a previous non-pancreatic, non-periampullary malignancy without evidence of disease for > 5 years will be allowed to enter the trial.

3.4.16 History of severe hypersensitivity reaction to any monoclonal antibody.

3.4.17 Patient has a known or suspected hypersensitivity to GM-CSF, hetastarch, corn, dimethyl sulfoxide, fetal bovine serum, trypsin (porcine origin), yeast or any other component of GVAX pancreas vaccine.

3.4.18 Women who are pregnant or breastfeeding.

3.4.19 Women with a positive pregnancy test on enrollment or prior to investigational product administration.

3.4.20 Women of child bearing potential or sexually active fertile men with partners who are women of child bearing potential who are unwilling or unable to use an

acceptable method to avoid pregnancy for the entire study and for up to 120 days after the last dose of investigational product.

3.4.21 Patient is unwilling or unable to follow the study schedule for any reason.

3.5 Inclusion of Women and Minorities

Both men and women of all races and ethnic groups are eligible for this trial.

4. TREATMENT PLAN

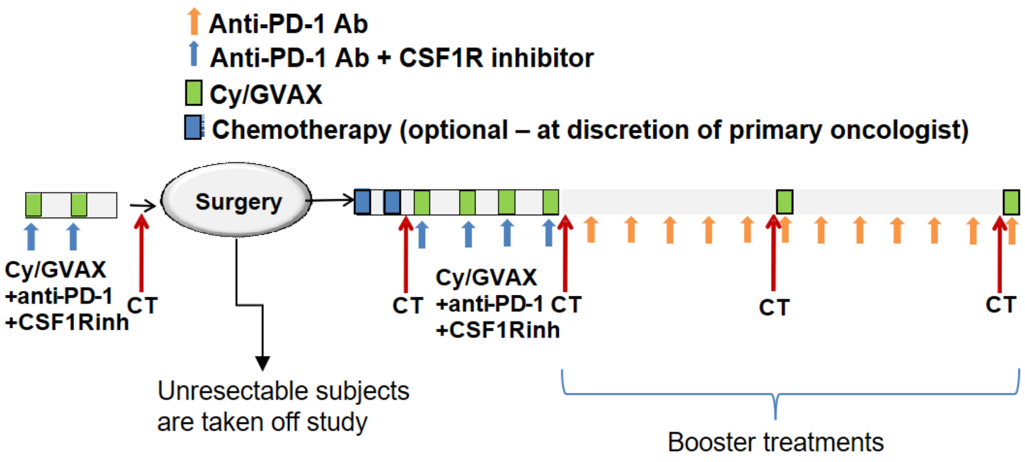

4.1 Agent Administration

Treatment will be administered on an outpatient basis. Dosing delays are described in **Section 5.3**. Subjects will receive two initial treatments of combined immunotherapy (pembrolizumab, immuno-modulatory dose of cyclophosphamide, GVAX, and IMC-CS4) separated by three weeks after completing neoadjuvant chemotherapy and/or radiation therapy and undergoing pre-screening for metastatic disease. One to two weeks after receiving the first two cycles of combination immunotherapy, patients with be reevaluated for surgery. If the patient remains a surgical candidate, he/she will undergo surgery two to three weeks following two cycles of neoadjuvant immunotherapy. Following surgery, the patient will have the option of receiving cycles of adjuvant chemotherapy at the discretion of primary oncologist. While the patient is receiving the adjuvant chemotherapy, she/he will not receive any immunotherapy. All patients who underwent surgical resection will receive adjuvant combination study immunotherapy every 3 weeks for another 4 cycles unless 1) BRPC Patients: more than 90 days have elapsed since surgery in the absence of adjuvant chemotherapy or >180 days for those who received adjuvant chemotherapy; Patients with resectable disease: will be allowed more than 180 days if a longer course of adjuvant chemotherapy is required or 2) there was unresectable or metastatic disease intraoperatively, or 3) there has been progression of disease.

Following the 4 adjuvant study therapy cycles, as long as the patient remains disease free and more than 90 days have not elapsed since the last immunotherapy treatment, the patients will receive 12 booster cycles of pembrolizumab every 3 weeks, and 2 booster cycles of CY/GVAX given every 18 weeks. The first booster CY/GVAX will be given when receiving the 6th booster dose of pembrolizumab (cycle 12), and the second booster CY/GVAX will be given when receiving the 12th booster dose of pembrolizumab (cycle 18).

Table 1: Study Regimen (listed in order of administration)

<table>
<tr><th>Agent</th><th>Premedications, Precautions</th><th colspan="2">Dose</th><th>Schedule</th><th>Route[1]</th></tr>
<tr><td>Cyclophosphamide (CY)</td><td>Subjects may be pre-medicated with anti-emetics</td><td colspan="2">200 mg/m2 in 100 mL NS</td><td>Q3W</td><td>IV infusion over 30 minutes</td></tr>
<tr><td>GVAX Pancreas Vaccine</td><td>EMLA cream (approximately 2.5gms/site at least 1 hour prior to vaccination)</td><td colspan="2">5x10^8 cells</td><td>Q3W</td><td>Six intradermal injections</td></tr>
<tr><td>Pembrolizumab</td><td>No prophylactic pre-medications unless indicated by previous experience in an individual subject</td><td colspan="2">200 mg</td><td>Q3W</td><td>IV over 30 minutes</td></tr>
<tr><td rowspan="2">IMC-CS4</td><td rowspan="2">No prophylactic pre-medications unless indicated by previous experience in an individual subject</td><td>Dose Level 1 (DL1)</td><td>75 mg</td><td>Q1W</td><td rowspan="2">IV infusion duration will depend on dose number and presence or absence of previous infusion reactions[2]</td></tr>
<tr><td>Dose Level 2 (DL2)</td><td>100 mg</td><td>Q1W</td></tr>
</table>

[1] Infusion times are approximate (+/- 10 min) and may need to be adjusted based on subject tolerability.

[2] The first dose of IMC-CS4 should be administered over 90 minutes. If there was no infusion reaction to the first dose, then the next dose can be administered over 60 minutes. The third and subsequent doses can be administered over 30 minutes if there were no previous infusion reactions.

Please see **Section 5.3** for guidance regarding dosing delays. Subjects that are required to stop treatment with pembrolizumab and IMC-CS4 due to toxicity may continue to receive CY/GVAX pancreas vaccine (assessment schedule per **Section 9**).

4.1.1 Cyclophosphamide (CY)

Subjects may be pre-medicated prior to administration with anti-emetics per

institutional guidelines.

Dosing calculation based on weight:
The dosing calculations should be based on the body weight. If the subject's weight on the day of dosing differs by > 10% from the weight used to calculate the dose, the dose must be recalculated.

Subjects should be observed for a minimum of 30 minutes after administration of CY, and before administration of GVAX.

4.1.2 GVAX Pancreas Vaccine

The vaccine consists of equal numbers (2.5 $\times 10^8$ irradiated cells each) of Panc 6.03 pcDNA1GM-CSF and Panc 10.05 pcDNA1GM-CSF cells combined for a total vaccination of 5 $\times 10^8$ cells. Vaccine cells from each tumor cell line frozen at 1.25 $\times 10^8$ cells/vial (2 vials per cell line) in an injectable formulation of Hespan (6% hetastarch in 0.9% sodium chloride injection, 2% human serum albumin, 5% dimethyl sulfoxide [DMSO]) will be thawed on the day of vaccination, combined and split into 6 syringes for administration. The vaccine has been previously irradiated with 150 Gy prior to storage. Each vaccination will consist of six total intradermal injections: 2 each in the upper right and left thighs, and 2 in the non-dominant arm. If one of these limbs is contraindicated, the dominant arm may be used. A lidocaine-based topical anesthetic (may include, but is not limited to, EMLA or ELA-MAX) cream will be applied to the injection site at least 1 hour prior to vaccination to diminish the discomfort associated with intradermal injections.

Subjects should be observed for a minimum of 30 minutes after administration of GVAX, and before administration of pembrolizumab and a minimum of 30 minutes before administration of IMC-CS4.

4.1.3 Pembrolizumab (KEYTRUDA®, MK-3475)

Pembrolizumab will be administered IV over 30 minutes at 200 mg every 3 weeks (Q3W). The choice of the 200 mg Q3W as an appropriate dose for the switch to fixed dosing is based on simulations performed using the population PK model of pembrolizumab showing that the fixed dose of 200 mg Q3W will provide exposures that 1) are optimally consistent with those obtained with the 2 mg/kg dose Q3W, 2) will maintain individual patient exposures in the exposure range established in melanoma as associated with maximal efficacy response and 3) will maintain individual patients exposure in the exposure range established in melanoma that are well tolerated and safe.

The Pharmacy Manual contains specific instructions for pembrolizumab administration. Antiemetic medications should not be routinely administered prior to dosing of pembrolizumab (if needed, patient will already be receiving anti-emetics prior to cyclophosphamide per institutional guidelines). See

Section 4.2.3.1 for subsequent premedication recommendations following a pembrolizumab-related infusion reaction.

Subjects should be observed for a minimum of 30 minutes after administration of Pembrolizumab, and before administration of pembrolizumab and a minimum of 30 minutes before administration of IMC-CS4.

4.1.4 CSF1R inhibitor IMC-CS4

IMC-CS4 is a human IgG1 monoclonal antibody designed to target the CSF1R. Premedication is not recommended to be administered prior to the first infusion of IMC-CS4. The recommended starting dose of IMC-CS4 is DL1 (Dose Level 1) administered as an intravenous infusion every week.

The first dose of IMC-CS4 should be administered over 90 minutes. If there was no infusion reaction to the first dose, then the next dose can be administered over 60 minutes. The third and subsequent doses can be administered over 30 minutes if there were no previous reactions.

The study will comprise of <u>two parts</u> for assessment of the safety and efficacy of IMC-CS4 dose escalation.

- Part One of the study will involve three patients who will receive a flat dose of IMC-CS4 at Dose Level 1 (DL1) (**Table 1**). They will be monitored for unacceptable toxicities (as defined in **Section 4.5**) and potential immune-related adverse events through the first two cycles of immunotherapy and subsequent surgical resection. If there is ≤ 1 patient during Part One who develops an unacceptable toxicity, then the study will progress to Part Two.

- Part Two of the study will enroll the remaining 9 subjects in the study, who will receive IMC-CS4 at DL2, which is the IMC-CS4 dose that is expected to be the recommended phase 2 dose from JSCA. DL2 will not be initiated until a minimum of 6 patients have been treated with 100 mg flat dosing cohort in JSCA for at least one cycle and ≤ 1 patient experience a dose-limiting toxicity in JSCA. If a subject develops an unacceptable toxicity on DL2, the next dose of IMC-CS4 will be held pending improvement of toxicity to grade 1 or 2. These subjects will undergo dose modification and transition to the DL1 dosing for subsequent cycles of immunotherapy. Subsequently enrolled patients will still be initiated on DL2, and the study will be halted for safety evaluation if the Bayesian stopping guideline (outlined in **Section 12.2** and **Table 9**) is met.

4.2 General Concomitant Medication and Supportive Care Guidelines

4.2.1 Cyclophosphamide (CY)

Acute reactions will be managed using standard therapy for acute drug reactions as per institutional guidelines.

4.2.2 GVAX Pancreas Vaccine

Local vaccine site reaction may be treated with topical applications of aloe vera or vitamin E gel or lotion. Significant local inflammation that is causing the subject severe pain or is interfering with the activities of daily living may be treated with oral analgesics. Local toxicities of pruritus at the vaccine sites and systemic pruritus may be treated with topical or oral diphenhydramine hydrochloride (Benadryl®) or topical aloe vera. If oral diphenhydramine hydrochloride is used the recommended dose shall be 25-50 mg every four to six hours as needed for pruritus, not to exceed 300 mg/day. Cases of local ulceration should be manageable with local wound care, with or without antibiotics. Severe local inflammation or significant clinical autoimmunity will be managed on a case-by-case basis.

4.2.3 Pembrolizumab

Pembrolizumab is a humanized monoclonal antibody. Subjects should be closely monitored for potential adverse reactions during antibody infusion and potential adverse events throughout the study.

Unexpected Grade 3 or greater laboratory abnormalities should be repeated within 24-72 hours if clinically indicated and monitored as necessary to determine if event meets toxicity criteria.

4.2.3.1 Infusion Reactions

Pembrolizumab infusion reactions may consist of fever, chills/rigor, nausea, pruritus, angioedema, hypotension, headache, bronchospasm, urticaria, rash, vomiting, myalgia, dizziness or hypertension. Severe reactions may include acute respiratory distress syndrome, myocardial infarction, ventricular fibrillation, and cardiogenic shock. Patients should be closely monitored for such reactions. Guidelines for patients who experience an infusion related or allergic reaction during or after infusion with pembrolizumab are shown below in **Table 2**.

Table 2: Guidance on Infusion and Hypersensitivity Reactions for Pembrolizumab

NCI CTCAE Grade	Treatment	Premedication at subsequent dosing
Grade 1<br>Mild reaction; infusion interruption not indicated; intervention not indicated	Increase monitoring of vital signs as medically indicated until the subject is deemed medically stable in the opinion of the investigator.	None
Grade 2<br>Requires infusion interruption but responds promptly to symptomatic treatment (e.g., antihistamines, NSAIDS, narcotics, IV fluids); prophylactic medications indicated for < =24 hrs	**Stop Infusion and monitor symptoms.**<br>Additional appropriate medical therapy may include but is not limited to:<br>IV fluids<br>Antihistamines<br>NSAIDS<br>Acetaminophen<br>Narcotics<br>Increase monitoring of vital signs as medically indicated until the subject is deemed medically stable in the opinion of the investigator.<br>If symptoms resolve within one hour of stopping drug infusion, the infusion may be restarted at 50% of the original infusion rate (e.g., from 100 mL/hr to 50 mL/hr). Otherwise dosing will be held until symptoms resolve and the subject should be premedicated for the next scheduled dose.<br>**Subjects who develop Grade 2 toxicity despite adequate premedication should be permanently discontinued from further trial treatment administration.**	Subject may be premedicated 1.5h (± 30 minutes) prior to infusion of pembrolizumab (MK-3475) with:<br><br>Diphenhydramine 50 mg po (or equivalent dose of antihistamine).<br><br>Acetaminophen 500-1000 mg po (or equivalent dose of antipyretic).
Grades 3 or 4<br>Grade 3:<br>Prolonged (i.e., not rapidly responsive to symptomatic medication and/or brief interruption of infusion); recurrence of symptoms following initial improvement; hospitalization indicated for other clinical sequelae (e.g., renal impairment, pulmonary infiltrates)<br>Grade 4:<br>Life-threatening; pressor or ventilatory support indicated	**Stop Infusion.**<br>Additional appropriate medical therapy may include but is not limited to:<br>IV fluids<br>Antihistamines<br>NSAIDS<br>Acetaminophen<br>Narcotics<br>Oxygen<br>Pressors<br>Corticosteroids<br>Epinephrine<br><br>Increase monitoring of vital signs as medically indicated until the subject is deemed medically stable in the opinion of the investigator.<br>Hospitalization may be indicated.<br>**Subject is permanently discontinued from further trial treatment administration.**	No subsequent dosing
Appropriate resuscitation equipment should be available in the room and a physician readily available during the period of drug administration.		

4.2.4 IMC-CS4 (LY3022855)

IMC-CS4 is a humanized monoclonal antibody. It is recommended that patients receiving IMC-CS4 should be monitored for serum levels of CK; markers of liver function (eg, levels of transaminases, LDH, bilirubin, coagulation disorders); muscle function; bone function; and signs indicative of inflammation (eg, leukocyte alterations, C-reactive protein) and facial/periorbital swelling.

Unexpected Grade 3 or greater laboratory abnormalities should be repeated within 24-72 hours if clinically indicated and monitored as necessary to determine if event meets toxicity criteria.

4.2.4.1 Infusion Reactions

As with other monoclonal antibodies, infusion-related reactions to IMC-CS4 (LY3022855) may occur. Monitor patients during and following the infusion for signs and symptoms of infusion-related reactions from the start of the infusion until at least 1 hour after the end of the infusion, in an area containing resuscitation equipment and medications necessary for advanced life support and cardiopulmonary resuscitation. For Grade 1 or 2 infusion-related reactions, adjust IMC-CS4 (LY3022855) dose per **Table 3**. Immediately and permanently discontinue IMC-CS4 (LY3022855) for Grade 3 or 4 infusion- related reactions.

Table 3: Guidance on Infusion and Hypersensitivity Reactions for IMC-CS4

NCI CTCAE Grade	Treatment	Premedication at subsequent dosing
Grade 1<br>Mild reaction; infusion interruption not indicated; intervention not indicated	The infusion rate should be decreased by 50% for the duration of the infusion.<br><br>Increase monitoring of vital signs as medically indicated until the subject is deemed medically stable in the opinion of the investigator.	Diphenhydramine 50 mg po (or equivalent dose of antihistamine).<br><br>Additional pre-medication may be administered at the investigator's discretion.
Grade 2<br>Requires infusion interruption but responds promptly to symptomatic treatment (e.g., antihistamines, NSAIDS, narcotics, IV fluids); prophylactic medications indicated for < =24 hrs	**The infusion must be stopped until resolution** to Grade ≤1; the infusion may then be resumed at 50% of the prior infusion rate.<br><br>Increase monitoring of vital signs as medically indicated until the subject is deemed medically stable in the opinion of the investigator.	Subject may be pre-medicated 1.5h (± 30 minutes) prior to infusion of LY3022855 with:<br><br>Diphenhydramine 50 mg po (or equivalent dose of antihistamine).<br><br>Additional pre-medication may be administered at the investigator's discretion.
Grades 3 or 4<br>Grade 3:<br>Prolonged (i.e., not rapidly responsive to symptomatic medication and/or brief interruption of infusion); recurrence of symptoms following initial improvement; hospitalization indicated for other clinical sequelae (e.g., renal impairment, pulmonary infiltrates)<br>Grade 4:<br>Life-threatening; pressor or ventilatory support indicated	**Stop Infusion.**<br><br>Increase monitoring of vital signs as medically indicated until the subject is deemed medically stable in the opinion of the investigator.<br>Hospitalization may be indicated.<br><br>**Subject is permanently discontinued from further trial treatment administration.**	No subsequent dosing
Appropriate resuscitation equipment should be available in the room and a physician readily available during the period of drug administration.		

4.2.5 Immune-Related Adverse Events (IRAEs) for Pembrolizumab and IMC-CS4

Subjects should receive appropriate supportive care measures as deemed necessary by the treating investigator. Suggested supportive care measures for the management of adverse events with potential immunologic etiology are outlined below. Where appropriate, these guidelines include the use of oral or intravenous treatment with corticosteroids as well as additional anti-inflammatory agents if symptoms do not improve with administration of

corticosteroids. Note that several courses of steroid tapering may be necessary as symptoms may worsen when the steroid dose is decreased. For each disorder, attempts should be made to rule out other causes such as metastatic disease or bacterial or viral infection, which might require additional supportive care. The treatment guidelines are intended to be applied when the investigator determines the events to be related to pembrolizumab and IMC-CS4 (LY3022855).

Note: if after the evaluation the event is determined not to be related, the investigator does not need to follow the treatment guidance (as outlined below). It may be necessary to perform conditional procedures such as bronchoscopy, endoscopy, or skin photography as part of evaluation of the event. NCCN guidelines have outlined management for immunotherapy related toxicities

- **Pneumonitis:**

  - For **Grade 2** events, treat with systemic corticosteroids. When symptoms improve to Grade 1 or less, steroid taper should be started and continued over no less than 4 weeks.
  - For **Grade 3-4** events, immediately treat with intravenous steroids. Administer additional anti-inflammatory measures, as needed.
  - Add prophylactic antibiotics for opportunistic infections in the case of prolonged steroid administration.

- **Diarrhea/Colitis:**

  Subjects should be carefully monitored for signs and symptoms of enterocolitis (such as diarrhea, abdominal pain, blood or mucus in stool, with or without fever) and of bowel perforation (such as peritoneal signs and ileus).

  - All subjects who experience diarrhea/colitis should be advised to drink liberal quantities of clear fluids. If sufficient oral fluid intake is not feasible, fluid and electrolytes should be substituted via IV infusion. For Grade 2 or higher diarrhea, consider GI consultation and endoscopy to confirm or rule out colitis.
  - For **Grade 2** diarrhea/colitis, administer oral corticosteroids.
  - For **Grade 3 or 4** diarrhea/colitis, treat with intravenous steroids followed by high dose oral steroids.
  - When symptoms improve to Grade 1 or less, steroid taper should be started and continued over no less than 4 weeks.

- **Type 1 diabetes mellitus (T1DM) (if new onset, including diabetic ketoacidosis [DKA]) or ≥ Grade 3 Hyperglycemia, if associated with ketosis (ketonuria) or metabolic acidosis (DKA)**

- For T1DM or Grade 3-4 Hyperglycemia
  - Insulin replacement therapy is recommended for Type I diabetes mellitus and for Grade 3-4 hyperglycemia associated with metabolic acidosis or ketonuria.
  - Evaluate patients with serum glucose and a metabolic panel, urine ketones, glycosylated hemoglobin, and C-peptide.

- **Hypophysitis:**

  - For **Grade 2** events, treat with corticosteroids. When symptoms improve to Grade 1 or less, steroid taper should be started and continued over no less than 4 weeks. Replacement of appropriate hormones may be required as the steroid dose is tapered.
  - For **Grade 3-4** events, treat with an initial dose of IV corticosteroids followed by oral corticosteroids. When symptoms improve to Grade 1 or less, steroid taper should be started and continued over no less than 4 weeks. Replacement of appropriate hormones may be required as the steroid dose is tapered.

- **Hyperthyroidism or Hypothyroidism:**

  Thyroid disorders can occur at any time during treatment. Monitor patients for changes in thyroid function (at the start of treatment, periodically during treatment, and as indicated based on clinical evaluation) and for clinical signs and symptoms of thyroid disorders.

  - **Grade 2** hyperthyroidism events (and **Grade 2-4** hypothyroidism):
    - In hyperthyroidism, non-selective beta-blockers (e.g. propranolol) are suggested as initial therapy.
    - In hypothyroidism, thyroid hormone replacement therapy, with levothyroxine or liothyroinine, is indicated per standard of care.
  - **Grade 3-4** hyperthyroidism
    - Treat with an initial dose of IV corticosteroid followed by oral corticosteroids. When symptoms improve to Grade 1 or less, steroid taper should be started and continued over no less than 4 weeks. Replacement of appropriate hormones may be required as the steroid dose is tapered.

- **Hepatic:**

  - Elevated LFTs can occur secondary to IMC-CS4 and may improve by holding drug.
  - For **Grade 2** events, hold immunotherapy and monitor liver function tests more frequently until returned to baseline values (consider every 3-5 days). Grade 2 elevated LFTs that do not improve to $\leq$ grade 1 after

holding immunotherapy drugs for two weeks should be treated with systemic corticosteroids.
  - For **Grade 3-4** events, treat with intravenous corticosteroids for 24 to 48 hours followed by oral steroids.

- **Renal Failure or Nephritis:**

  - For **Grade 2** events, treat with corticosteroids.
  - For **Grade 3-4** events, treat with systemic corticosteroids.
  - When symptoms improve to Grade 1 or less, steroid taper should be started and continued over no less than 4 weeks.

- **Elevated CK:**

  - For **Grade 2** events that have clinical or laboratory signs of end organ damage (e.g. elevated urine and serum myoglobin) hold IMC-CS4 (Refer to **Table 4**).
  - For **Grade 3-4** events hold IMC-CS4 (Refer to **Table 4**).
  - Based on the mechanism of action of IMC-CS4, elevated CK levels are expected (J Immunother Cancer. 2017; 5:53; Am J Pathol. 2011 Jul;179 (1): 240-247). Since there is not clear inflammatory basis for these reactions, the need for steroids is questionable and holding drug until CK decreases can be advised. Corticosteroids could be considered in cases with clinical or laboratory signs indicative of immune mediated tissue inflammation.

- **All other drug-related toxicity:**
  - For **Grade 3-4** events, treat with systemic steroids.
  - When symptoms improve to Grade 1 or less, steroid taper should be started and continued over no less than 4 weeks.

4.2.6. Prohibited and Restricted Therapies

Patients may not use any of the following agents during the study:

- Any non-study anticancer or immunotherapy agent (investigational or non-investigational)

- Any other investigational agents

- Any other immunotherapy treatment, including, but not limited to: IL-2, interferon, anti-PD-1, anti-PD-L1, anti-PD-L2, anti-CD137, anti-OX-40, anti-CD40, or anti-CTLA-4 antibodies

- Systemic glucocorticoids for any purpose other than to modulate symptoms from an event of clinical interest of suspected immunologic etiology. The use of physiologic doses of corticosteroids may be approved after consultation with the IND Sponsor. Steroid treatment should be completed at least 14 days prior to resuming study-related treatments. However, study-related treatments may be given after short-term steroid use ($\leq$ 4 days) with prior approval by the Principal Investigator and IND Sponsor (See **Section 5.3** for dosing delays for steroids). Further, if patients require high dose steroids, while on study, pembrolizumab can be re-administered if the steroids are tapered over a 12 week period. Steroids used as premedication for contrast CT allergies can be utilized when clinically indicated and do not require a 14 days washout.

- Filgrastim (Neupogen® or G-CSF) or sargramostim (Leukine® or GM-CSF)

- Live vaccines (examples of live vaccines include, but are not limited to: measles, mumps, rubella, chicken pox, yellow fever, rabies, BCG, and typhoid [oral] vaccine). Seasonal influenza vaccines for injection are generally killed virus vaccines and are allowed. However, intranasal influenza vaccines (e.g. Flu-Mist®) are live attenuated vaccines, and are not allowed.

4.3 Definition of an Overdose for this Protocol

Overdose of pembrolizumab or IMC-CS4 will be defined as:
The patient was given (accidentally or intentionally) a dose of 1000 mg or greater ($\geq$5 times the indicated dose) of pembrolizumab. The patient was given (accidentally or intentionally) a dose of 115 (which is 15% more than the recommended phase 2 dose) of IMC-CS4. No specific information is available on the treatment of overdose of pembrolizumab and IMC-CS4. In the event of overdose, pembrolizumab or IMC-CS4 should be discontinued and the subject should be observed closely for signs of toxicity. Appropriate supportive treatment should be provided if clinically indicated.

If an adverse experience(s) is associated with ("results from") the overdose of test drug or vaccine, the adverse experience(s) is reported as a serious adverse experience, even if no other criteria for serious are met.

If a dose of test drug or vaccine meeting the protocol definition of overdose is taken without any associated clinical symptoms or abnormal laboratory results, the overdose is reported as a non-serious Event of Clinical Interest (ECI), using the terminology "accidental or intentional overdose without adverse effect."

All reports of overdose with and without an adverse experience must be reported within 24 hours to the IND Sponsor, Merck Global Safety, and Eli Lilly local safety representative (contact information can be found in **Section 6.5.1)**.

4.4 Contraception, Use in Pregnancy, Use in Nursing

4.4.1 Contraception

The investigational agents may have adverse effects on a fetus *in utero*. Furthermore, it is not known if the investigational agents have transient adverse effects on the composition of sperm.

Given the potential risk related to pharmacologically-mediated inhibition of the PD-1 pathway, no reproductive or developmental toxicity studies were conducted with pembrolizumab. IMC-CS4 has also not been studied in pregnant women and must not be given to patients who are pregnant. Furthermore, animal reproduction studies have not been conducted with IMC-CS4. Therefore, potential effects of IMC-CS4 use during pregnancy are not known and the potential for developmental toxicity cannot be excluded.

For this trial, male subjects will be considered to be of non-reproductive potential if they have azoospermia (whether due to having had a vasectomy or due to an underlying medical condition).

Female subjects will be considered of non-reproductive potential if they are either:

(1) postmenopausal (defined as at least 12 months with no menses without an alternative medical cause; in women < 45 years of age a high follicle stimulating hormone (FSH) level in the postmenopausal range may be used to confirm a post-menopausal state in women not using hormonal contraception or hormonal replacement therapy. In the absence of 12 months of amenorrhea, a single FSH measurement is insufficient.);

OR

(2) have had a hysterectomy and/or bilateral oophorectomy, bilateral salpingectomy or bilateral tubal ligation/occlusion, at least 6 weeks prior to screening;

OR

(3) has a congenital or acquired condition that prevents childbearing.

Female and male subjects of reproductive potential must agree to avoid becoming pregnant or impregnating a partner, respectively, while receiving study drug and for 120 days after the last dose of study drug by complying with one of the following:

(1) practice abstinence† from heterosexual activity;

OR

(2) use (or have their partner use) acceptable contraception during heterosexual activity.

Acceptable methods of contraception are‡:

Single method (one of the following is acceptable):

- intrauterine device (IUD)
- vasectomy of a female subject's male partner
- contraceptive rod implanted into the skin

Combination method (requires use of two of the following):

- diaphragm with spermicide (cannot be used in conjunction with cervical cap/spermicide)
- cervical cap with spermicide (nulliparous women only)
- contraceptive sponge (nulliparous women only)
- male condom or female condom (cannot be used together)
- hormonal contraceptive: oral contraceptive pill (estrogen/progestin pill or progestin-only pill), contraceptive skin patch, vaginal contraceptive ring, or subcutaneous contraceptive injection

†Abstinence (relative to heterosexual activity) can be used as the sole method of contraception if it is consistently employed as the subject's preferred and usual lifestyle and if considered acceptable by local regulatory agencies and ERCs/IRBs. Periodic abstinence (e.g., calendar, ovulation, sympto-thermal, post-ovulation methods, etc.) and withdrawal are not acceptable methods of contraception.

‡If a contraceptive method listed above is restricted by local regulations/guidelines, then it does not qualify as an acceptable method of contraception for subjects participating at sites in this country/region.

Subjects should be informed that taking the study medication may involve unknown risks to the fetus (unborn baby) if pregnancy were to occur during the study. In order to participate in the study subjects of childbearing potential must adhere to the contraception requirement (described above) from the day of study medication initiation (or 14 days prior to the initiation of study medication for oral contraception) throughout the study period up to 120 days after the last dose of trial therapy. If there is any question that a subject of childbearing potential will not reliably comply with the requirements for contraception, that subject should not be entered into the study.

Non-pregnant, non-breast-feeding women may be enrolled if they are considered highly unlikely to conceive. Highly unlikely to conceive is defined as 1) surgically sterilized, or 2) postmenopausal (a woman who is ≥45 years of age and has not had menses for greater than 2 years will be considered postmenopausal), or 3) amenorrheaic for <2 years without a hysterectomy and oophorectomy and with a documented FSH value in the postmenopausal range, or 4) not heterosexually active for the duration of the study, or 5) heterosexually active and willing to use 2 methods of birth control (which is also required for the female partners of male patients). The 2 birth control methods can be 2 barrier methods *or* a barrier method plus a hormonal method to prevent pregnancy, used throughout the study starting with Visit 1 through 120 days after the last dose of study medication. Male patients enrolled in this study must also agree to use an adequate method of contraception starting with Visit 1 through 120 days after the last dose of study drug.

The following are considered adequate barrier methods of contraception (2 barrier methods are required): diaphragm, condom (by the partner), copper intrauterine device, sponge, or spermicide. Appropriate hormonal contraceptives will include any registered and marketed contraceptive agent that contains an estrogen and/or a progestational agent (including oral, subcutaneous, intrauterine, or intramuscular agents).

Patients should be informed that taking the study medication may involve unknown risks to the fetus (unborn baby) if pregnancy were to occur during the study. In order to participate in the study they must adhere to the contraception requirement (described above) for the duration of the study. If there is any question that a patient will not reliably comply with the requirements for contraception, that patient should not be entered into the study.

4.4.2 Use in Pregnancy

The investigational agents may have adverse effects on a fetus; therefore, women with a positive pregnancy test at screening will not be eligible for enrollment. If a patient inadvertently becomes pregnant while on treatment with combination immunotherapy, the patient will immediately be removed from the study. The site will contact the patient at least monthly and document the patient's status until the pregnancy has been completed or terminated. The outcome of the pregnancy will be reported to the IND Sponsor, to Merck Global Safety, and to Eli Lilly local safety representative without delay. The outcome must be reported to the IND Sponsor within 24 hours, Merck Global Safety and Eli Lilly local safety representative if the outcome is a serious adverse experience (e.g., death, abortion, congenital anomaly, or other disabling or life-threatening complication to the mother or newborn). If a male patient's partner becomes pregnant on study the pregnancy must be reported to the IND Sponsor, Merck

Global Safety and Eli Lilly local safety representative as described in **Section 6.5.1**. The study Investigator will make every effort to obtain permission to follow the outcome of the pregnancy and report the condition of the fetus or newborn to the IND Sponsor, Merck Global Safety and Eli Lilly local safety representative.

4.4.3 Use in Nursing Women

It is unknown whether the investigational agents are excreted in human milk. Since many drugs are excreted in human milk, and because of the potential for serious adverse reactions in the nursing infant, patients who are breast-feeding are not eligible for enrollment.

4.5 Unacceptable Toxicities

Unacceptable toxicities are defined as any AE that is temporally related to study drug administration and is not due to the subject's underlying malignancy and for which there is no clear evidence for an alternative etiology and meets one of the following NCI CTCAE criteria:

Hematologic Toxicities

− Grade 4 anemia

− Grade 3 or 4 neutropenia lasting ≥ 14 days

− Grade ≥ 3 neutropenia with fever

− Grade 4 thrombocytopenia, or Grade 3 thrombocytopenia with clinically significant bleeding

Other Toxicities

− Treatment-related ≥ grade 4 AEs

− Grade 3 Pneumonitis

− Grade 3 Nephritis

− Grade 3 elevated LFTs that do not improve to ≤ grade 2 after holding IMC-CS4 for two weeks.

- Grade 4 creatine kinase elevation >10 × ULN

− Grade ≥ 2 eye pain or reduction of visual acuity that does not respond to topical therapy and does not improve to ≤ grade 1 severity within 2 weeks of starting therapy, or requires systemic therapy

− Any other clinically significant Grade ≥ 3 toxicity that do not improve to ≤ grade 2 under therapy within 2 weeks except for the following:

- o Grade 3 nausea, vomiting, or diarrhea that resolves to Grade ≤ 1 within 7 days, with or without appropriate supportive therapy
- o Grade ≥ 3 rash that resolves to Grade ≤ 2 within 7 days

- Grade 3 fatigue that resolves to ≤ Grade 2 within 14 days
- Grade ≥ 3 laboratory abnormalities that, in the judgment of the investigator, are not clinically significant
- Asymptomatic amylase/lipase elevation

Unexpected Grade 3 or greater laboratory abnormalities should be repeated within 24-72 hours if clinically indicated and monitored as necessary to determine if event meets toxicity criteria. Hyponatremia has been reported and will be monitored.

The proportion of unacceptable toxicities will be monitored. If the toxicity levels are unacceptable (high probability that it is >30% of subjects), then enrollment will be suspended until further review and consideration by the IND Sponsor and MEC.

4.6 Criteria for Removal from Study Treatment

Patients will be removed from study treatment when any of the criteria listed below applies. The reason for study treatment removal and the date the patient was removed must be documented in the Case Report Form.

A subject must be discontinued from the trial for any of the following reasons:

- The subject or legal representative (such as a parent or legal guardian) withdraws consent, or
- Patient is lost to follow-up.

A subject must be discontinued from treatment (but may continue to be monitored in the post-treatment follow-up portion of the trial) for any of the following reasons:

- The subject or legal representative (such as a parent or legal guardian) withdraws consent for treatment,
- Development of local recurrence or distant metastatic disease,
- Patients who were found to have unresectable disease intraoperatively,
- Intercurrent illness that prevents further administration of treatment,
- Unacceptable adverse event(s) (see **Section 4.5**),
- Investigator's decision to withdraw the subject,
- Severe or life-threatening pembrolizumab or IMC-CS4-related AE(s) per **Table 4**,

- Need for >2 dose delays due to the same toxicity as per the dose delay guidelines. However, if patients have undergone surgery and subsequently develop grade $\geq$ 3 irAEs to pembrolizumab or IMC-CS4, they can be allowed to continue CY/GVAX booster therapy, at the discretion of the investigator,

- Inability to reduce corticosteroid dose for immune-related adverse reactions to ≤10 mg prednisone or equivalent per day,

- BRPC Patients: More than 90 days have elapsed since the last immunotherapy treatment (except for the adjuvant study immunotherapy, which needs to be restarted within 90 days of surgery if no adjuvant chemotherapy was provided or within 180 days if adjuvant chemotherapy is provided). Patients with resectable disease will be allowed more than 180 days if a longer course of adjuvant chemotherapy is required,

- If in the opinion of the Investigator, a change or temporal or permanent discontinuation of therapy would be in the best interest of the patient. The IND Sponsor should be included in this decision,

- Noncompliance with trial treatment or procedure requirements, or

- Patient becomes pregnant.

4.7 Off Study/Safety Follow-up Visit

After a patient is discontinued from treatment, a mandatory Off Study/Safety Follow-Up Visit should be performed approximately 30 days after the last infusion of study medication (or within 7 days prior to initiation of a new anti-cancer treatment, whichever comes first). Procedures and assessments performed at this visit and beyond should follow the respective guidelines described in **Section 9** as appropriate. The patient will be monitored for adverse events up to the mandatory Off Study/Safety Follow-Up Visit or to resolution of toxicity to Grade 0-1, whichever occurs later. Serious Adverse Events (SAEs) that occur within 90 days of the last infusion of pembrolizumab or before initiation of a new antineoplastic treatment should also be followed and recorded.

4.8 Duration of Follow Up

Subjects who discontinue from treatment should continue to follow up with their primary oncologist and be contacted every six months (with a +/- 1 month window) to monitor OS and for potential disease progression. Information of other cancer therapies after discontinuation from the study treatment will be collected.

Subjects who are discontinued from the study treatment due to an unacceptable drug-related AE will be monitored for safety until the resolution of the AE to ≤ grade 1 or stabilization or until initiation of a new therapy for their cancer, whichever occurs first.

All subjects will be followed for at least 30 days after their last dose of study drug for the development of AEs. SAEs that occur within 90 days of the last infusion of pembrolizumab or before initiation of a new antineoplastic treatment should also be followed and recorded.

5. DOSING DELAYS/DOSE MODIFICATIONS

5.1 Dose Modifications

5.1.1 Dose reduction or dose increase of CY, GVAX, and pembrolizumab will not be permitted in individual patients.

5.1.2 Part One of the study will evaluate the initial three patients enrolled in the study for IMC-CS4 dose-related toxicities in particular transaminitis and CK elevation which have occurred during the single agent phase 1 study with IMC-CS4.

During Part One, patients will receive IMC-CS4 at DL1. DL1 (75 mg) is one dose level below recommended phase 2 dose of IMC-CS4 per phase 1 single agent studies to allow this evaluation. Subjects will be monitored for the number of grade 3 or higher toxicities through the first two cycles of immunotherapy.

5.1.2.1 During Part One, further dose-reduction beyond DL1 will be at the discretion of the principal investigator and upon discussions PI or IND sponsor.

5.1.3 Part Two of the study will be initiated pending monitoring of the initial Part One. If there is ≤ 1 patient who develops unacceptable toxicities in Part One, the study will progress to Part Two, where dosing of IMC-CS4 will be at Dose-Level 2 (DL2). DL2 (100 mg) is dose that is expected to be the recommended phase 2 dose from JSCA. DL2 will not be initiated until minimum of 6 patients have been treated with 100 mg flat dosing cohort in JSCA for at least one cycle and ≤ 1 patient experience a dose-limiting toxicity in JSCA. Dose modifications and management of LFTs toxicities will occur the same as during Part One.

5.1.4 The following table provides dose levels of the study:

Dosing Table for Part 1 and Part 2

Dose of IMC-CS4	Dose
DL-1	50 mg
DL1	75 mg
DL2	100 mg

5.2 Dosing Delays

5.2.1. All scheduled neoadjuvant and adjuvant treatments within a cycle are to be given approximately 3 weeks apart. If necessary, a cycle may be delayed for up to 1 week. In this case, subsequent cycles should continue so that a subject can still receive all cycles given that the cycles are a minimum of 3 weeks apart and they have not experienced an AE(s) necessitating discontinuation. If delayed more than 1 week, the Principal Investigator must be contacted for further instructions on continued treatment. Additional delays or modifications to the treatment schedule must be approved by the PI or IND Sponsor.

5.2.2 Pembrolizumab and IMC-CS4 will be withheld for suspected drug-related toxicities and severe or life-threatening AEs as per **Table 4**. Modifications for IMC-CS4 and discontinuation criteria are specified in **Table 4**.

5.2.3 Dosing of study therapy will be delayed if the following laboratory criteria are not met:

Neoadjuvant Study Treatment (Day 1, 8, and 15 of Cycles 1 and 2)

Absolute neutrophil count	≥ 1,000 cells/mm^3
Hemoglobin	≥ 9g/dL
Platelets	≥ 100,000 cells/mm^3
Serum creatinine	≤ 1.5 x ULN*
AST and ALT	≤ 3.0 x ULN
Total bilirubin	≤ 1.5 x ULN **

Adjuvant Study Treatment (Day 1, 8, and 15 of Cycles 3-6)

Absolute neutrophil count	≥ 1,000 cells/mm^3
Hemoglobin	≥ 9g/dL
Platelets	≥ 100,000 cells/mm^3
Serum creatinine	≤ 1.5 x ULN*
AST and ALT	≤ 3.0 x ULN
Total bilirubin	≤ 1.5 x ULN **

Boosting Study Treatment (Day 1 of Cycle 7-18)

Absolute neutrophil count	≥ 1,000 cells/mm^3
Hemoglobin	≥ 9g/dL
Platelets	≥ 100,000 cells/mm^3
Serum creatinine	≤ 1.5 x ULN*
AST and ALT	≤ 3.0 x ULN
Total bilirubin	≤ 1.5 x ULN**

*If the serum creatinine is > 1.5 x ULN, but the estimated GFR is > 30 mL/min, then the booster therapy can be administered.

**Subjects with Gilbert's Syndrome should have direct bilirubin within normal institutional limits

Table 4: Pembrolizumab and IMC-CS4 Dose Delay, Modifications and Discontinuation Criteria

Toxicity	Hold Treatment For Grade	Timing for Restarting Pembrolizumab Treatment	Timing for Restarting IMC-CS4 Treatment and Dose Modifications	Treatment Discontinuation
Diarrhea/Colitis	2-3	Toxicity resolves to Grade 0-1.	Toxicity resolves to Grade 0-1.	Toxicity does not resolve within 12 weeks of last dose or inability to reduce corticosteroid to 10 mg or less of prednisone or equivalent per day within 12 weeks.
	4	Permanently discontinue	Permanently discontinue	Permanently discontinue
AST[1], ALT, or Increased Bilirubin	2[1]	Toxicity resolves to Grade 0-1	First occurrence: If resolution within two weeks to Grade ≤ 1 can restart IMC-CS4 at same dose after symptoms resolve.<br><br>Second occurrence: If resolution within two weeks to Grade ≤ 1 can restart IMC-CS4 with reduction of one dose level.	Toxicity does not resolve within 12 weeks of last dose.
	3	Toxicity resolves to Grade 1-2	Toxicity resolves to Grade 1-2 Restart at one dose level reduction	Permanently discontinue IF toxicity continues after two week period.
	4	Permanently discontinue	Permanently discontinue	Permanently discontinue
CK[2]	2 with clinical or laboratory signs of end organ damage (e.g. elevated urine and serum myoglobin)<br><br>3	Toxicity resolves to Grade 0-1	Toxicity resolves to Grade 0-1	Toxicity does not resolve within 12 weeks of last dose
	4	Permanently discontinue	Permanently discontinue	Permanently discontinue
Type 1 diabetes mellitus (if new onset) or Hyperglycemia	T1DM or 3-4	Hold treatment for new onset Type 1 diabetes mellitus or Grade 3-4 hyperglycemia associated with evidence of beta cell failure.	Hold treatment for new onset Type 1 diabetes mellitus or Grade 3-4 hyperglycemia associated with evidence of beta cell failure.	Resume pembrolizumab and IMC-CS4 when patients are clinically and metabolically stable.
Hypophysitis	2-4	Toxicity resolves to Grade 0-1. Therapy can be continued while endocrine replacement therapy is instituted	Toxicity resolves to Grade 0-1. Therapy can be continued while endocrine replacement therapy is instituted	Toxicity does not resolve within 12 weeks of last dose or inability to reduce corticosteroid to 10 mg or less of prednisone or equivalent per day within 12 weeks.

Toxicity	Hold Treatment For Grade	Timing for Restarting Pembrolizumab Treatment	Timing for Restarting IMC-CS4 Treatment and Dose Modifications	Treatment Discontinuation
Hyperthyroidism	3	Toxicity resolves to Grade 0-1	Toxicity resolves to Grade 0-1	Toxicity does not resolve within 12 weeks of last dose or inability to reduce corticosteroid to 10 mg or less of prednisone or equivalent per day within 12 weeks.
	4	Permanently discontinue	Permanently discontinue	Permanently discontinue
Hypothyroidism		Therapy with pembrolizumab and IMC-CS4 can be continued while thyroid replacement therapy is instituted	Therapy with pembrolizumab and IMC-CS4 can be continued while thyroid replacement therapy is instituted	Therapy with pembrolizumab can be continued while thyroid replacement therapy is instituted.
Infusion Reaction	3-4	Permanently discontinue	Permanently discontinue	Permanently discontinue
Pneumonitis	2	Toxicity resolves to Grade 0-1	Toxicity resolves to Grade 0-1	Toxicity does not resolve within 12 weeks of last dose or inability to reduce corticosteroid to 10 mg or less of prednisone or equivalent per day within 12 weeks. Permanently discontinue for recurrent grade 2 toxicity.
	3-4	Permanently discontinue	Permanently discontinue	Permanently discontinue
Renal Failure or Nephritis	2	Toxicity resolves to Grade 0-1	Toxicity resolves to Grade 0-1	Toxicity does not resolve within 12 weeks of last dose or inability to reduce corticosteroid to 10 mg or less of prednisone or equivalent per day within 12 weeks.
	3-4	Permanently discontinue	Permanently discontinue	Permanently discontinue
All Other Drug-Related Toxicity[3]	3	Toxicity resolves to Grade 0-1	Toxicity resolves to Grade 0-1	Toxicity does not resolve within 12 weeks of last dose or inability to reduce corticosteroid to 10 mg or less of prednisone or equivalent per day within 12 weeks.
	4	Permanently discontinue	Permanently discontinue	Permanently discontinue

Note: Permanently discontinue for any severe or Grade 3 drug-related AE that recurs or any life-threatening event.

[1] Patients who develop isolated Grade 2 AST elevation (i.e. without concurrent ALT or bilirubinemia to suggest a liver source of transaminase) may continue dosing with combined immunotherapy of pembrolizumab and IMC-CS4 since this elevation is thought to be due to pharmacodynamics effect rather than direct muscle toxicity per the IND Sponsor. However, if there is grade 2 AST elevation with concurrent ALT or bilirubin elevation, then adjust treatment was written above for grade 2 AST, ALT, bilirubin abnormality.

[2] Patients who develop Grade 2 CK elevation will be allowed continued dosing with combined immunotherapy of pembrolizumab and IMC-CS4 as long as there is no clinical or laboratory signs of end organ damage and (2) urine and serum myoglobin are within normal range.

[3] Patients with intolerable or persistent Grade 2 drug-related AE may hold study medication at physician discretion. Permanently discontinue study drug for persistent Grade 2 adverse reactions for which treatment with study drug has been held, that do not recover to Grade 0-1 within 12 weeks of the last dose.

5.2.2.1 If any of the above events in **Table 4** occur, the investigator should discuss with the Principal Investigator to make a decision on discontinuation of pembrolizumab study treatment. In case toxicity does not resolve or improve to ≤ Grade 1 within 12 weeks after last administration of study drug, study therapy discontinuation should be considered after discussion with the Principal Investigator and IND Sponsor. With IND Sponsor and Drug Sponsor agreement, patients still at Grade 2 may continue in the study only if asymptomatic and controlled. Two dosing delays due to the same toxicity will be permitted. In the event of a third occurrence of the same toxicity which would require dosing delay, study therapy will be discontinued permanently, except in select cases of pembrolizumab-only irAEs, at which time post-surgical resection patients may be eligible for continuation of CY/GVAX booster therapy.

5.2.2.2 In case toxicity does not resolve to Grade 0-1 within 12 weeks after last infusion, pembrolizumab and/or IMC-CS4 should be discontinued after consultation with the IND Sponsor. With IND Sponsor and drug companies' agreement, subjects with a laboratory adverse event still at Grade 2 after 12 weeks may continue treatment in the trial only if asymptomatic and controlled.

5.2.3 If a delay occurs between day 1 and 2 of a cycle:

- Pembrolizumab and IMC-CS4 related infusion reactions must resolve to baseline prior to administration of GVAX.
- Day 2 GVAX treatment and assessments can be resumed without repeating Day 1 study treatments (Cy, pembrolizumab, and IMC-CS4) if the delay is within 72 hours.
- If the delay is longer than 72 hours, repeat Day 1 and Day 2 study treatments/assessments with a minimum of 2 weeks from the previous Day 1 treatment. This includes steroid treatment requiring at least a 14-day washout prior to resuming study-related treatments.

Subjects who have completed surgery that are required to stop treatment with pembrolizumab or IMC-CS4 due to toxicity may stay on study and receive CY/GVAX pancreas vaccine to complete the four-adjuvant (6 total treatments) once pembrolizumab-related or IMC-CS4 related toxicity(s) has resolved to a grade 1.

5.2.4 Patients should ideally undergo surgical resection within 7-12 weeks from last radiation dose if this was given in the neoadjuvant setting.. If treatment delays occur during the administration of neoadjuvant immunotherapy, it should be discussed between treating physician, study PI and treating surgeon as it may impact the date for surgical resection. If deemed clinically necessary patients will proceed with surgical resection as planned and still allowed to receive adjuvant therapy per protocol as long as they had received one full cycle of neoadjuvant immunotherapy.

5.2.5 Patients who develop immune related toxicities while receiving neoadjuvant immunotherapy can receive shorter courses of steroids when clinically appropriate. This would minimize complications during standard of care surgery secondary to prolonged use of steroids.

6. ADVERSE EVENTS: LIST AND REPORTING REQUIREMENTS

This study will use the descriptions and grading scales found in the revised National Cancer Institute Common Terminology Criteria for Adverse Events (CTCAE) Version 4.0 for adverse event reporting that can be found at http://ctep.cancer.gov/protocolDevelopment/electronic_applications/ctc.htm.

Information about all adverse events, whether volunteered by the subject, discovered by investigator questioning, or detected through physical examination, laboratory test or other means, will be collected, recorded, and followed as appropriate.

The PI has the primary responsibility for continuous internal monitoring for safety, protocol compliance, and identification, grading, coding, and required reporting of all anticipated and unanticipated adverse events and protocol problems. Although this responsibility is usually shared among the PI, research nurse, and data manager, the PI is ultimately responsible for grading and attribution of all events.

6.1 Definitions

6.1.1 Adverse Event (AE)

Adverse event is defined as any undesirable sign, symptom or medical condition occurring after starting the study drug (or therapy) even if the event is not considered to be related to the study. An undesirable medical condition can be symptoms (e.g., nausea, chest pain), signs (e.g., tachycardia, enlarged liver) or the abnormal results of an investigation (e.g., laboratory findings, electrocardiogram). Medical conditions/diseases present before starting the study treatment are only considered adverse events if they worsen after starting the study treatment (any procedures specified in the protocol). Adverse events occurring before starting the study treatment but after signing the informed consent form will not be recorded. Abnormal laboratory values or test results constitute adverse events only if they induce clinical signs or symptoms or require therapy.

Laboratory abnormalities: Laboratory abnormalities present at the screening visit will be recorded as pre-treatment signs and symptoms. After study treatment administration, all grade 3 and 4 clinical laboratory results that represent an increase in severity from baseline will be reported as adverse events.

A grade 1 or 2 clinical laboratory abnormality should be reported as an adverse event only if it is considered clinically significant by the investigator.

6.1.2 Serious Adverse Event (SAE)

A serious adverse event (SAE) is an undesirable sign, symptom or medical condition which:

• Results in death
• Is life threatening (defined as an event in which the subject was at risk of death at the time of the event; it does not refer to an event which hypothetically might have caused death if it were more severe)
• Requires inpatient hospitalization or causes prolongation of existing hospitalization (see note below for exceptions) > 24 hours
• Results in persistent or significant disability/incapacity
• Is a congenital anomaly/birth defect (note: reports of congenital anomalies/birth defects must also be reported on the Pregnancy Supplemental Form)
• Is an important medical event (defined as a medical event(s) that may not be immediately life-threatening or result in death or hospitalization but, based upon appropriate medical and scientific judgment, may jeopardize the subject or may require intervention [eg, medical, surgical] to prevent one of the other serious outcomes listed in the definition above.). Examples of such events include, but are not limited to, intensive treatment in an emergency room or at home for allergic bronchospasm; blood dyscrasias or convulsions that do not result in hospitalization.)
• Is a new cancer (that is not a condition of the study)
• Is associated with an overdose

Events **not** considered to be serious adverse events are hospitalizations for the:

• Admissions as per protocol for a planned medical/surgical procedure or to facilitate a procedure
• Routine health assessment requiring admission for baseline/trending of health status (eg, routine colonoscopy)
• Medical/surgical admission for purpose other than remedying ill health state and was planned prior to entry into the study. Appropriate documentation is required in these cases
• Admission encountered for another life circumstance that carries no bearing on health status and requires no medical/surgical intervention (eg, lack of housing, economic inadequacy, care-giver respite, family circumstances, administrative).

6.1.3 Events of Clinical Interest (ECI)

Events of clinical interest for this trial include:

- An overdose of study drug, as defined in **Section 4.3**, that is not associated with clinical symptoms or abnormal laboratory results.

- An elevated AST or ALT lab value that is greater than or equal to 3X the upper limit of normal and an elevated total bilirubin lab value that is greater than or equal to 2X the upper limit of normal and, at the same time, an alkaline phosphatase lab value that is less than 2X the upper limit of normal, as determined by way of protocol-specified laboratory testing or unscheduled laboratory testing.*

*Note: These criteria are based upon available regulatory guidance documents. The purpose of the criteria is to specify a threshold of abnormal hepatic tests that may require an additional evaluation for an underlying etiology.

6.2 Relationship

The relationship of an AE to the administration of the study drug is to be assessed by the investigator according to the following definitions:

- No (unrelated, not related, no relation): The time course between the administration of study drug and the occurrence or worsening of the adverse event rules out a causal relationship and another cause (concomitant drugs, therapies, complications, etc.) is suspected.

- Yes (related): The time course between the administration of study drug and the occurrence or worsening of the adverse event is consistent with a causal relationship and no other cause (concomitant drugs, therapies, complications, etc.) can be identified.

The following factors should also be considered:

- The temporal sequence from study drug administration - The event should occur after the study drug is given. The length of time from study drug exposure to event should be evaluated in the clinical context of the event.
- Underlying, concomitant, intercurrent diseases - Each report should be evaluated in the context of the natural history and course of the disease being treated and any other disease the subject may have.
- Concomitant medication - The other medications the subject is taking or the treatment the subject receives should be examined to determine whether any of them might be recognized to cause the event in question.
- Known response pattern for this class of study drug - Clinical and/or preclinical data may indicate whether a particular response is likely to be a class effect.
- Exposure to physical and/or mental stresses - The exposure to stress might induce adverse changes in the recipient and provide a logical and better explanation for the event.

The pharmacology and pharmacokinetics of the study drug - The known pharmacologic

properties (absorption, distribution, metabolism, and excretion) of the study drug should be considered.

Assessment of Grade:

The investigator will make an assessment of grade for each AE and SAE reported during the study, which will be recorded in the CRF. The assessment will be based on the National Cancer Institute's CTCAE (Version 4.0) and graded as shown below:

- Grade 1: Mild; asymptomatic or mild symptoms; clinical or diagnostic observations only; intervention not indicated
- Grade 2: Moderate; minimal, local or noninvasive intervention indicated; limiting age-appropriate instrumental activities of daily living
- Grade 3: Severe or medically significant but not immediately life-threatening; hospitalization or prolongation of hospitalization indicated; disabling; limiting self-care activities of daily living
- Grade 4: Life-threatening consequences; urgent intervention indicated
- Grade 5: Death related to AE

Any AE that changes in grade during its course will be recorded in the CRF at the highest level experience by the subject.

6.3 Expectedness

Unexpected adverse event: An adverse event, which varies in nature, intensity or frequency from information on the investigational drug/agent provided in the Investigator's Brochure, package insert or safety reports. Any adverse event that is not included in the informed consent is considered "unexpected".

Expected (known) adverse event: An adverse event, which has been reported in the Investigator's Brochure. An adverse event is considered "expected", only if it is included in the informed consent document as a risk.

6.4 Handling of Expedited Safety Reports

In accordance with local regulations, the IND Sponsor will notify investigators of all SAEs that are unexpected (i.e. not previously described in the Investigator Brochure), and definitely, probably, or possibly related to pembrolizumab, IMC-CS4, or CY/GVAX. This notification will be in the form of an expedited safety report (ESR) that is to be faxed to the investigators and the study coordinators. Upon receiving such notices, the investigator must review and retain the notice with the Investigator's Brochure and where required by local regulations, the investigator will submit the ESR to the appropriate IRB. The investigator and IRB will determine if the informed consent requires revision. The investigator should also comply with the IRB procedures for reporting any other safety information.

6.5 Reporting

6.5.1 General

All adverse events (both expected and unexpected) will be captured on the appropriate study-specific case report forms (CRFs). Adverse events experienced by subjects will be collected and reported from the first dose of the study drug, throughout the study, and will only be followed for 30 days (or within 7 days prior to initiation of a new anti-cancer treatment, whichever comes first) unless related to the study drug(s). Adverse events related to the study drug(s) will be monitored for resolution of toxicity to ≤ grade 1, stabilization, or determined to be irreversible by the investigator.

Surgery and chemotherapy (when applicable) are considered standard of care (SOC). Patients will be considered off treatment between day 30 of cycle 2 through day -1 of cycle 3, while they are receiving SOC treatment. Adverse events will be reviewed to ensure that the combination of CY, GVAX, pembrolizumab, and IMC-CS4 are not contributing to postoperative complications. Surgical complications will be collected in a separate log, but will not be recorded as adverse events unless determined to be related to immunotherapy. Please refer to **Section 6.6** for the assessment and recording of surgical AEs. Unrelated adverse events that occur during the off study period between cycles 2 and 3 of treatment will not be recorded on the CRFs.

ECIs will be collected and reported from the first dose of study drug through 30 days after the last dose of study drug (or within 7 days prior to initiation of a new anti-cancer treatment, whichever comes first). ECIs must be reported within 24 hours to the IND Sponsor (email: ejaffee@jhmi.edu), Merck Global Safety (Attn: Worldwide Product Safety; Fax 215 993-1220), and Eli Lilly (Attn: Local Safety representative; Fax: +1-866-644-1697) using the Adverse Event of Clinical Interest Case Report Form found in **Appendix B**.

SAEs that occur from the first dose of study drug to within 90 days of the last infusion of pembrolizumab (or before initiation of a new antineoplastic treatment, whichever occurs first), with the exception of unrelated SAEs that occur during the off study period between cycles 2 and 3 of immunotherapy (day 30 of cycle 2 through day -1 of cycle 3) should also be followed and recorded. **All SAEs, regardless of causality to study drug, will be reported promptly to the IND Sponsor (email: ejaffee@jhmi.edu), Merck Global Safety (Attn: Worldwide Product Safety; Fax 215-993-1220), and Eli Lilly (Attn: Local Safety representative; Fax: +1-866-644-1697) within 24 hours of recognition of the adverse event using the form found in Appendix C**. If this falls on a weekend or holiday, an email notification is acceptable but must be followed by an SAE reporting form on the next business day.

After the initial SAE report, the investigator is required to proactively follow each subject and provide further information to the safety department in regards to the subject's condition.

All SAE(s) will be followed until:

- Resolution
- The condition stabilizes
- The event is otherwise explained
- The subject is lost to follow-up

As soon as relevant information is available, a follow-up SAE report will be submitted to the IND Sponsor, Merck GS, and Eli Lilly local safety representative.

Although pregnancy and lactation are not considered adverse events, it is the responsibility of investigators or their designees to report any pregnancy or lactation in a subject (spontaneously reported to them), including the pregnancy of a male subject's female partner who has provided written consent to provide information regarding pregnancy, that occurs during the trial or within 120 days of completing the trial. All subjects who become pregnant must be followed to the completion/termination of the pregnancy. Pregnancy outcomes of spontaneous abortion, missed abortion, benign hydatidiform mole, blighted ovum, fetal death, intrauterine death, miscarriage and stillbirth must be reported as serious adverse events (Important Medical Events). If the pregnancy continues to term, the outcome (health of infant) must also be reported to the IND Sponsor, Merck GS, and Eli Lilly local safety representative.

6.5.2 Institutional Review Board (IRB) and Institutional Biosafety Committee (IBC)

Serious adverse events will be reported to the IRB and IBC per institutional guidelines. Follow-up information will be submitted to the IRB and IBC as soon as relevant information is available.

6.5.3 Food and Drug Administration (FDA)

All reporting to the FDA will be completed by the IND Sponsor.

6.5.3.1 Expedited IND Safety Reports:

7 Calendar-Day Telephone or Fax Report:
The IND Sponsor is required to notify the FDA of any fatal or life-threatening adverse event that is unexpected and assessed by the investigator to be possibly related to the investigational agent. Such reports are to be telephoned or faxed

(301-827-9796) to the FDA within 7 calendar days of first learning of the event.

15 Calendar-Day Written Report:
The IND Sponsor is required to notify the FDA of any serious adverse event that is unexpected and possibly related to the investigational agent in a written IND Safety Report.

Written IND Safety Reports should include an Analysis of Similar Events in accordance with regulation 21 CFR § 312.32. All safety reports previously filed with the IND concerning similar events should be analyzed. The new report should contain comments on the significance of the new event in light of the previous, similar reports. Follow-up information will be submitted to the FDA as soon as relevant information is available.

Written IND safety reports with Analysis of Similar Events are to be submitted to the FDA within 15 calendar days of first learning of the event.

6.5.3.2 IND Annual Reports

In accordance with the regulation 21 CFR § 312.33, the IND Sponsor shall within 60 days of the anniversary date that the IND went into effect submit a brief report of the adverse events and progress of the investigation. Please refer to Code of Federal Regulations, 21 CFR § 312.33 for a list of the elements required for the annual report. All IND annual reports will be submitted to the FDA by the IND Sponsor.

6.5.4 Recombinant DNA Advisory Committee (RAC)

Unexpected SAEs believed to be related to the investigational product(s) will be reported to RAC by email if fatal or life-threatening within 7 calendar days or within 15 calendar days if related and unexpected to the investigational product(s). SAEs that are unrelated or related and expected with the investigational product (s) will be reported to RAC in the Annual Report. Follow-up information will be submitted to the RAC as soon as relevant information is available.

6.6 Special Considerations for Adverse Events that Occur During the Surgery and Postoperative Course

Pancreatic surgery is one of the highest-risk procedures, requires prolonged hospitalization, has significant toxicities and is commonly associated with complications and comorbidities. It is deemed to be standard of care, therefore, is not part of this research study. From the day of surgery through the initiation of adjuvant immunotherapy, participants will be primarily followed by their primary surgeons for monitoring and managing the complications and comorbidities attributable to the

surgery. **Table 5** summarizes complications commonly associated with the pancreatic surgery based on several published analyses of more than 1000 patients at Johns Hopkins Medicine and other institutions[44] A normal, uncomplicated postoperative course is still commonly associated with laboratory abnormalities (grade 1/2 AEs and occasionally grade 3 AEs) without need of therapeutic interventions. At least one complication was associated with 58.5% of patients based on a recent analysis of a consecutive series of 633 patients undergoing pancreaticoduodenectomy at Johns Hopkins Medicine between February 2003 and August 2005. Grade I, II, III complications were common, in 10%, 30%, 13.5% of the patients, respectively. These complications and comorbidities are commonly associated with toxicities and laboratory abnormalities of CTCAE grade 3 and even grade 4. By contrast, Grade IV and V complications are relatively uncommon, in 3.0% and 2.0% of the patients, respectively **(Table 6)**.

Table 5: Complications after Pancreaticoduodenectomy

Anastomotic leak, pancreas	Anastomotic leak, intestinal
Wound infection	Gastrointestinal bleeding
Delayed gastric emptying	Pleural effusion
Hemorrhage, immediate postoperative or delayed	Pneumonitis
Intraabdominal abscess	Sepsis
Fascial dehiscence or evisceration	Acute respiratory distress syndrome
Supraventricular arrhythmia	Angina, cardiac ischemia
Urinary tract infection	Aspiration
Anastomotic leak, biliary	Cardiopulmonary arrest
Pancreatitis	Catheter-related infection
Hypotension, shock	Constipation
Cellulitis	Delerium tremens
Clostridium difficile colitis	Fever
Congestive heart failure, left ventricular dysfunction	Fluid imbalance
Myocardial infarction	Gastroesophageal reflux disease
Renal failure	Congestive heart failure
Apnea or hypoxia	Ileus
Atelectasis	Interstitial pneumonitis and fibrosis
Catheter-related infection	Prolonged intubation
Deep venous thrombosis	Salivary gland infection
Dehydration	Small bowel obstruction

Table 6: Classification of Surgical Complication Adopted for Pancreatic Surgery

Grade	Definition
I	Any deviation from the normal postoperative course without pharmacologic treatment or surgical, endoscopic, and radiological interventions. Allowed therapeutic regimens are: drugs as antiemetics, antipyretics, analgesics, diuretics, electrolytes, and physiotherapy. This grade also includes wound infections opened at the bedside.
II	Requiring pharmacologic treatment with drugs other than ones allowed for grade I complications. Blood transfusion and total parenteral nutrition* are also included.
III	Requiring surgical, endoscopic, or radiologic intervention
IIIa	Intervention not under general anesthesia
IIIb	Intervention under general anesthesia
IV	Life-threatening complication (including CNS complications)† requiring IC/ICU management
IVa	Single-organ dysfunction (including dialysis)
IVb	Multiorgan dysfunction
V	Death of a patient
Suffix "d"	If the patient suffers from a complication at the time of the discharge, the suffix "d" (for disability) is added to the respective grade of complication (including resection of the pancreatic remnant). This label indicates the need for a follow-up to fully evaluate the complication.

*Note regarding DGE: The insertion of a central line for TPN or nasojejunal tube by endoscopy is a grade IIIa. However, if a central line is still in place or a feeding tube has been inserted at the time of surgery, then a TPN or enteral nutrition is a grade II complication.

†Brain hemorrhage, ischemic stroke, subarachnoidal bleeding, but excluding transient ischemic attacks.

CNS indicates central nervous system; IC, intermediate care; ICU, intensive care unit.

Pancreatic surgery is commonly associated with laboratory abnormalities in blood cell counts, serum electrolytes, liver function, and renal function, etc., with a range of severity from grade 1 to grade 4 by CTCAEv4.0 criteria. Grade I-III complications are common; therefore, laboratory abnormalities associated with a normal postoperative course or Grade I-III complications are considered to be within the commonly expected range of grades of severity (**Table 7**). Laboratory abnormalities beyond these commonly expected ranges of severity should be considered uncommon. Although Grade IV and V complications have still occurred, any SAE including laboratory abnormalities associated with Grade IV/V complications should be considered uncommon.

Table 7: Laboratory abnormalities commonly associated with pancreatic surgery

Lab test Abnormality	Ranges of severity by CTCAEv3.0 criteria	
Amylase	Elevated	Grade 1-4
Lipase	Elevated	Grade 1-4
Bilirubin	Elevated	Grade 1-4
AST	Elevated	Grade 1-4
ALT	Elevated	Grade 1-4
Albumin	Decreased	Grade 1-3
Glucose	Elevated	Grade 1-4
Glucose	Decreased	Grade 1-4
Alk Phosphatase	Elevated	Grade 1-4
Creatinine	Elevated	Grade 1-3
Glomerular filtration rate	Decreased	Grade 1-3
Bicarbonate	Decreased	Grade 1-4
Acidosis	Elevated	Grade 1-4
Alkylosis	Elevated	Grade 1-4
CPK	Elevated	Grade 1-4
WBC	Elevated	Not graded by CTCAE
Hemoglobin	Decreased	Grade 1-3
Platelets	Elevated	Not graded by CTCAE
Platelets	Decreased	Grade 1-3
Sodium	Elevated	Grade 1-3
Sodium	Decreased	Grade 1-3
Potassium	Elevated	Grade 1-3
Potassium	Decreased	Grade 1-3
Magnesium	Elevated	Grade 1-3
Magnesium	Decreased	Grade 1-3
Phosphate	Elevated	Grade 1-3
Phosphate	Decreased	Grade 1-4
Calcium	Elevated	Grade 1-3
Calcium	Decreased	Grade 1-4

Therefore during this period, first, the study will be focused on monitoring and reporting the complications with uncommon grades of severity such as Grade IV and Grade V complications by criteria used at Johns Hopkins (**Table 6**). The severity of any SAEs associated with such grades of complications should be considered

uncommon. Second, any unusual complications not seen commonly with this operation will be reported. Third, the study will also be focused on monitoring and reporting any laboratory abnormality beyond the common ranges of severity (**Table 7**). Fourth, the study will also be focused on monitoring and reporting any type of toxicity not commonly attributable to the surgery or postoperative course. These events will be recorded as described in **Section 6** and their severities will still be categorized by NCI CTCAEv4.0 criteria. Relationship of these events to the investigational drug will be determined by the principal investigator together with surgical co-investigators of the study team and, if necessary, with primary surgeons, and will be categorized as described in **Section 6.2**. Reporting of these events will follow the same guidelines described in **Sections 6.5**.

7. PHARMACEUTICAL INFORMATION

7.1 Cyclophosphamide (Cytoxan®, CY)

7.1.1 Agent Accountability

The IND Sponsor or the Sponsor's representative shall take responsibility for and shall take all steps to maintain appropriate records and ensure appropriate supply, storage, handling, distribution and usage of investigational product in accordance with the protocol and any applicable laws and regulations.

7.1.2 Mode of Action

CY is a synthetic antineoplastic drug chemically related to the nitrogen mustards. CY is biotransformed principally in the liver to active alkylating metabolites by a mixed function microsomal oxidase system. These metabolites interfere with the growth of susceptible rapidly proliferating malignant cells. The mechanism of action is thought to involve cross-linking of tumor cell DNA.

7.1.3 Description

CY (CYTOXAN®; cyclophosphamide for injection, USP) is a sterile, white powder containing cyclophosphamide monohydrate and is supplied in vials for single-dose use.

7.1.4 Packaging and Labeling Information

CY is commercially available.

7.1.5 Preparation

Parenteral drug products should be inspected visually for particulate matter and discoloration prior to administration, whenever solution and container permit.

Add the diluent to the vial and shake it vigorously to dissolve. If the powder fails to dissolve immediately and completely, it is advisable to allow the vial to stand for a few minutes. Use the quantity of diluent shown below to constitute the product:

Dosage Strength	CYTOXAN Contains Cyclophosphamide Monohydrate	Quantity of Diluent
500 mg	534.5 mg	25 mL
1 g	1069.0 mg	50 mL
2 g	2138.0 mg	100 mL

CY may be prepared for parenteral use by infusion using any of the following methods:

1. CY constituted with 0.9% sterile sodium chloride may be infused without further dilution.
2. CY constituted with 0.9% sterile sodium chloride may be infused following further dilution in the following:

    - Dextrose Injection, USP (5% dextrose)
    - Dextrose and Sodium Chloride Injection, USP (5% dextrose and 0.9% sterile sodium chloride)
    - 5% Dextrose and Ringer's Injection
    - Lactated Ringer's Injection, USP
    - Sodium Chloride Injection, USP (0.45% sterile sodium chloride)
    - Sodium Lactate Injection, USP (1/6 molar sodium lactate)

7.1.6 Storage

Store vials at or below 77° F (25° C).

7.1.7 Stability

CY (prepared for either direct injection or infusion) is chemically and physically stable for 24 hours at room temperature or for 6 days in the refrigerator; it does not contain any antimicrobial preservative and thus care must be taken to assure the sterility of prepared solutions.

7.1.8 Administration

CY is administered at 200 mg/m2 in 100 mL NS by IV injection over 30 minutes.

7.1.9 Subject Care Implications

During treatment, the subject's hematologic profile (particularly neutrophils and platelets) should be monitored regularly to determine the degree of

hematopoietic suppression.

The rate of metabolism and the leukopenic activity of CY reportedly are increased by chronic administration of high doses of phenobarbital. The physician should be alert for possible combined drug actions, desirable or undesirable, involving CY even though CY has been used successfully concurrently with other drugs, including other cytotoxic drugs. CY treatment, which causes a marked and persistent inhibition of cholinesterase activity, potentiates the effect of succinylcholine chloride. If a subject has been treated with CY within 10 days of general anesthesia, the anesthesiologist should be alerted.

CY may interfere with normal wound healing.

7.1.10 Returns and Reconciliation

N/A

7.2 GVAX Pancreas Vaccine

7.2.1 Agent Accountability

The IND Sponsor or the Sponsor's representative shall take responsibility for and shall take all steps to maintain appropriate records and ensure appropriate supply, storage, handling, distribution and usage of investigational product in accordance with the protocol and any applicable laws and regulations.

7.2.2 Mode of Action

GM-CSF-secreting, irradiated, whole cell vaccines recruit and activate tumor-specific T cells and induce a cytotoxic response through two mechanisms: 1. they deliver a range of peptide antigens (without the need for specific knowledge of the relevant target antigens), and 2. GM-CSF is an important growth and differentiation factor for dendritic cells, which are potent antigen-presenting cells.

7.2.3 Description

GVAX pancreas vaccine, PANC 10.05 pcDNA-1/GM-Neo Clinical Lot (Irradiated) and PANC 6.03 pcDNA-1/GM-Neo Clinical Lot (Irradiated) is a white, milky suspension in a 2 ml cryovial. Each of the pancreatic tumor cell lines have been genetically-modified with a plasmid vector encoding the cDNA for human GM-CSF and subsequently cultured and irradiated.

The vaccine consists of equal numbers (2.5×10^8 each) of irradiated Panc

6.03pcDNA1GM-CSF and Panc 10.05pcDNA1GM-CSF cells combined into a single vaccination. The final vaccine population secretes approximately 80-100 ng/ 10^6 cells/ 24 hours of bioactive GM-CSF. Vaccine cells from each pancreas tumor cell line are frozen at 1.25×10^8 cells/vial (2 vials per cell line) in an injectable formulation of Hespan (6% hetastarch in 0.9% sodium chloride with 2% human serum albumin and 5% DMSO). The GVAX pancreas vaccine will be thawed on the day of vaccination and taken up into syringes.

7.2.4 Packaging and Labeling Information

Vial labels on each cell line will contain the cell line name, either PANC 10.05 pcDNA-1/GM-Neo Clinical Lot, (Irradiated) or PANC 6.03 pcDNA-1/GM-Neo Clinical Lot (Irradiated) along with: the lot number, cell concentration and volume, date of manufacture, vial number, storage conditions, IND caution statement and manufacturer.

7.2.5 Preparation

At the time of vaccination, the vials are removed from the appropriate storage conditions and quickly thawed in a 37°C water bath. The thawed vials are transferred from the water bath to wet ice until the vaccine is drawn from the vial with a 16 gauge needle into 1 cc luer-lock syringes and then changed to 22 gauge luer-lock needles for subject's administration. The syringes are then released to the appropriate medical personnel for intradermal injection and are kept on ice until the vaccine is administered. All injections must be given within 3 hours of thaw. The 3 hours start when the vials are placed in the water bath for thaw.

7.2.6 Storage

PANC 10.05 pcDNA-1/GM-Neo Clinical Lot (Irradiated) and PANC 6.03 pcDNA-1/GM-Neo (Irradiated) must be stored in vapor phase liquid nitrogen, less than or equal to -135°C.

7.2.7 Stability

Once thawed, the GVAX pancreas vaccine must be administered to the subject within 3 hours.

7.2.8 Administration

$5x10^8$ GVAX pancreas vaccine cells will be administered in six intradermal injections: 2 in the right thigh, 2 in the left thigh, and 2 in the non-dominant arm.

7.2.9 Subject Care Implications

There is a possible risk of anaphylaxis and should be administered in a setting where emergency treatment is available. Administration should be immediately discontinued and the appropriate therapy instituted if there is a serious allergic reaction. The subject must be observed in the clinic for at least 60 minutes after the first set of injections and 30 minutes after subsequent injections. If an anaphylactic reaction is suspected, this must be discussed with the IND Sponsor and the Principal Investigator before the subject is given another dose of vaccine.

The vaccine should not be administered to subjects with a known hypersensitivity to GM-CSF, DMSO, hetastarch, trypsin (porcine origin), or fetal bovine serum. Subjects allergic to corn can also be allergic to hetastarch. Precautions include hypersensitivity reactions and anaphylaxis. At 5-fold human doses, hetastarch is embryocidal in animals.

The vaccine should not be administered to pregnant or nursing women, to subjects with active or untreated brain metastases, or to subjects with autoimmune disorders such as systemic lupus erythematosus, sarcoidosis, rheumatoid arthritis, glomerulonephritis, vasculitis, etc. It should not be administered to subjects who may be at increased risk for bleeding or infection from intradermal injections such as individuals who have coagulopathies or decreased bone marrow function.

Systemic corticosteroids and immunosuppressive therapy should be avoided as they may inhibit the effectiveness of the vaccine, however, intranasal, intra-articular or topical steroids are permitted. The interaction with other vaccines is unknown.

The vaccine should be used with caution in subjects with a history of cardiac arrhythmias, pericardial or pleural effusions, congestive heart failure, pulmonary infiltrates, or lung disease associated with hypoxia.

7.2.10 Returns and Reconciliation

The investigator is responsible for keeping accurate records of the clinical supplies, the amount administered to subjects, and the amount remaining at the conclusion of the trial.

7.3 Pembrolizumab (KEYTRUDA®, MK-3475)

7.3.1 Agent Accountability

The IND Sponsor or the Sponsor’s representative shall take responsibility for and shall take all steps to maintain appropriate records and ensure appropriate supply, storage, handling, distribution and usage of investigational product in

accordance with the protocol and any applicable laws and regulations.

7.3.2 Mode of Action

Pembrolizumab is a highly selective humanized monoclonal antibody designed to block the interaction between PD-1 and its ligands, PD-L1 and PD-L2. Pembrolizumab is an IgG4/kappa isotype with a stabilizing sequence alteration in the Fc region.

7.3.3 Description

Clinical Supplies will be provided by Merck as summarized below:

Product Name & Potency	Dosage Form
Pembrolizumab – 50 mg	Lyophilized Powder for Injection
Pembrolizumab – 100 mg / 4 mL	Solution for Infusion

7.3.4 Packaging and Labeling Information

Clinical supplies will be affixed with a clinical label in accordance with regulatory requirements. Open label kits will be provided for patient dosing.

7.3.5 Storage and Handling Requirements

Clinical supplies must be stored in a secure, limited-access location under the storage conditions specified on the label.

Receipt and dispensing of trial medication must be recorded by an authorized person.

Clinical supplies may not be used for any purpose other than that stated in the protocol.

7.3.6 Returns and Reconciliation

The investigator is responsible for keeping accurate records of the clinical supplies received from Merck or designee, the amount dispensed to and returned by the subjects and the amount remaining at the conclusion of the trial.

Upon completion or termination of the study, all unused and/or partially used investigational product will be destroyed at the site per institutional policy. It is the Investigator's responsibility to arrange for disposal of all empty containers, provided that procedures for proper disposal have been established according to applicable federal, state, local and institutional guidelines and

procedures, and provided that appropriate records of disposal are kept.

7.3.7 Administration

200 mg pembrolizumab will be administered intravenously over 30 minutes.

7.3.8 Patient Care Implications

Based on results from the nonclinical studies, there are currently no specific safety considerations. Pembrolizumab is a humanized monoclonal Ab. Thus far, serious infusion reactions have been infrequent and manageable. However, subjects should be closely monitored for potential adverse reactions during antibody infusion and potential adverse events throughout the study. In the event that a subject experiences an allergic reaction to pembrolizumab, treatment (i.e., vasopressors, H2-blockers, antihistamines, H1-blockers, steroids) should be administered, as appropriate, and prophylaxis should be considered.

Overall, pembrolizumab has been safe and well-tolerated. Immune-related AEs (irAEs) are of special interest because of the mechanism of action of anti-PD-1. Prompt identification of irAEs and appropriate treatment (e.g. withdrawal of study medication and treatment with corticosteroids) will be critical to optimizing the therapeutic index. See **Section 4.2.5** outline specific guidance for prompt identification and treatment of irAEs. Timely discussions of how to manage irAEs is also encouraged.

7.3.9 Agent Ordering

Requests for pembrolizumab should be sent via email to:
Debra Perlow
Email: debra.perlow@merck.com

7.3.10 Returns and Reconciliation

The investigator is responsible for keeping accurate records of the clinical supplies received from Merck or designee, the amount administered to patients, and the amount remaining at the conclusion of the trial.

Upon completion or termination of the study, all unused and/or partially used investigational product will be destroyed at the site per institutional policy. It is the Investigator's responsibility to arrange for disposal of all empty containers, provided that procedures for proper disposal have been established according to applicable federal, state, local and institutional guidelines and procedures, and provided that appropriate records of disposal are kept.

7.4 IMC-CS4/ LY3022855

7.4.1 Agent Accountability

The IND Sponsor or the Sponsor's representative shall take responsibility for and shall take all steps to maintain appropriate records and ensure appropriate supply, storage, handling, distribution and usage of investigational product in accordance with the protocol and any applicable laws and regulations.

7.4.2 Mode of Action

IMC-CS4 is a human IgG1 monoclonal antibody designed to target the colony stimulating factor 1 receptor (CSF-1R) to block the interaction between the CSF-1R and its ligand colony stimulating factor 1 (CSF1).

7.4.3 Description

IMC-CS4 drug product is a sterile, preservative-free injection for infusion of IMC-CS4 formulated in an aqueous solution at a concentration of 5 mg/mL (100 mg/20-mL vial). The buffer contains 10mM histidine, 100mM glycine, 100mM arginine, and 0.01% polysorbate 80.

IMC-CS4 drug product is a clear or slightly opalescent and colorless or slightly yellow liquid, without visible particles. The pH is 6.0. The osmolality is 293 mmol/kg. All excipients used for the manufacture of IMC-CS4 drug product are of pharmacopeial grade. No animal-derived components are used in the manufacture of IMC-CS4 drug product excipients.

7.4.4 Packaging and Labeling Information

IMC-CS4 drug product is supplied in single-use, 20-mL nominal volume, United States Pharmacopeia (USP) Type I glass vials. Each vial contains 100 mg of IMC-CS4 at a concentration of 5 mg/mL in a sterile, preservative-free solution. Each vial is stoppered with a coated chlorobutyl latex-free rubber stopper and sealed with an aluminum seal and a flip-off cap.

7.4.5 Preparation

The dose of IMC-CS4 should be aseptically withdrawn from the vial and transferred to a sterile IV container. IMC-CS4 is compatible with infusion containers composed of polyolefin, polyvinyl chloride, ethylene vinyl acetate, and evacuated glass (USP Type II or local equivalent). An infusion bag composed of polyolefin, polypropylene, and polyethylene prefilled with 0.9% sodium chloride injection, such as AVIVA, may also be used.

To administer using pre-filled IV infusion containers: Calculate the respective dose and remove the corresponding volume of normal saline from the prefilled 250-mL container of the correct composition. Aseptically transfer the calculated dose of IMC-CS4 drug product to the container to bring the final volume in the container back to 100 mL or 250 mL, as specified in the protocols. Gently invert the container to mix.

To administer using empty IV infusion containers: Aseptically transfer the calculated dose of IMC-CS4 drug product into an empty IV container of the correct composition and add a sufficient quantity of sterile normal saline (0.9% weight/volume) to the container to bring the total volume to 100 mL or 250 mL, as specified in the protocols. Gently invert the container to mix.

7.4.6 Storage

7.4.6.1 Drug Product

The drug product must be stored under refrigeration at 2°C to 8°C (36°F to 46°F) with protection from direct light. **DO NOT FREEZE AND/OR SHAKE LY3022855 DRUG PRODUCT.** Stability studies have demonstrated that the drug product can withstand transient excursion to room temperature without adverse effect; however, storage at this temperature is not recommended.

7.4.6.2 Dosing Solution for Infusion

Chemical and physical in-use stability for the prepared IMC-CS4 dosing solution has been demonstrated for up to 24 hours below 25°C (77°F). However, it is recommended that the prepared dosing solution be used immediately in order to minimize the risk of microbial contamination. If not used immediately, the prepared IMC-CS4 dosing solution should be stored under refrigeration at 2°C to 8°C (36°F to 46°F) for no longer than 24 hours. If the prepared solution is held at room temperature (below 25°C [77°F]), it must be used within 4 hours. Store protected from light. Brief exposure to ambient light is acceptable while preparation and administration is taking place. **DO NOT FREEZE AND/OR SHAKE PREPARED LY3022855 DOSING SOLUTION FOR INFUSION.**

7.4.7 Stability

Stability studies have demonstrated that the drug product can withstand transient excursion to room temperature without adverse effect; however, storage at this temperature is not recommended.

7.4.8 Administration

IMC-CS4 is administered as an IV infusion. The infusion rate should not exceed 25 mg/minute. Premedication is not recommended to be administered prior to the first infusion of IMC-CS4 If the patient experiences a Grade 1 or 2 infusion-

related reaction, premedication with diphenhydramine hydrochloride 50 mg IV (or equivalent) must be provided prior to any subsequent doses of IMC-CS4 Additional premedication may be administered at the investigator's discretion.

The Part One dosing of IMC-CS4 (LY3022855) is DL1 (75 mg) and is administered as an intravenous infusion. The Part Two dosing is DL2 (100 mg). In this study, IMC-CS4 will be administered intravenously every week. IMC-CS4 is administered as an IV infusion. The infusion rate should not exceed 25 mg/minute.

7.4.9 Patient Care Implications

As with other monoclonal antibodies, infusion-related reactions to IMC-CS4 may occur. Monitor patients during and following the infusion for signs and symptoms of infusion-related reactions. As a routine precaution, patients treated with IMC-CS4 should be closely monitored by the medical staff for signs and symptoms indicative of an infusion-related reaction from the start of the infusion until at least 1 hour after the end of the infusion.

It is recommended that patients receiving IMC-CS4 should be monitored for serum levels of CK; markers of liver function (eg, levels of transaminases, LDH, bilirubin, coagulation disorders); muscle function; bone function; and signs indicative of inflammation (eg, leukocyte alterations, C-reactive protein) and facial/periorbital swelling.

Toxicities encountered with immuno-oncology agents may represent an immune-related etiology, which may be based on the mechanism of action of those agents. These immune- mediated reactions, or immune-related adverse events (irAEs), can result in an inflammatory response in any organ or tissue. The irAEs may occur shortly after the first dose or several months after the last dose of treatment. Potential irAEs include immune-mediated enterocolitis, dermatitis, hepatitis, and endocrinopathies. Patients should be monitored for signs and symptoms of irAEs. In the absence of an alternate etiology (for example, infection or progressive disease), signs or symptoms of enterocolitis, dermatitis, pneumonitis, hepatitis, and endocrinopathy should be considered immune-related. Refer to **Section 4.2.5** for supportive care guidelines for immune-mediated reactions, including use of corticosteroids.

7.4.10 Agent Ordering

For ordering IMC-CS4, drug re-orders will be ordered from Eli Lilly by the Johns Hopkins Weinberg pharmacy personnel.

7.4.11 Returns and Reconciliation

The investigator is responsible for keeping accurate records of the clinical supplies, the amount dispensed to subjects, and the amount remaining at the conclusion of the trial.

Upon completion or termination of the study, all unused and/or partially used investigational product will be destroyed at the site per institutional policy. It is the Investigator's responsibility to arrange for disposal of all empty containers, provided that procedures for proper disposal have been established according to applicable federal, state, local and institutional guidelines and procedures, and provided that appropriate records of disposal are kept.

8. CORRELATIVE/SPECIAL STUDIES

8.1 Tumor Tissue and Whole Blood for Peripheral Blood Mononuclear Cells (PBMC) and Circulating Tumor Cells (CTCs) Studies

A research tumor biopsy will be collected (4 to 6 cores, fine needle aspiration will not be acceptable) during the scheduled endoscopy that will occur following the completion of chemotherapy and/or radiation and within 14 days prior to the first day of treatment. This post-radiation biopsy is not standard of care, but it is a mandatory part of the research study. If subjects have inaccessible tumors or for whom the biopsies are thought not in their best interest, they will be ineligible for the study. Additional biopsies (via surgical resection specimen) will also be requested of patients after completion of 2 doses of combined immunotherapy. Archival tumor samples may also be collected for every patient (slides and/or blocks). These tissue samples will be banked for the evaluation of PD-L1/PD-1, their associated immune suppressive pathways and other immune activation pathways to assess the effect of treatment upon the tumor microenvironment and the correlations between these immune parameters and clinical response.

Whole blood for PBMCs (up to 120cc) will be collected at the specified timepoints (See **Section 9**). Up to 20cc of whole blood may be collected for the isolation of circulating tumor cells. Detailed instructions for blood collection, processing, and storage are provided in the Laboratory Manual.

Immunohistochemistry, flow cytometry, quantitative PCR assays and microarray analysis will be employed. Peripheral Blood Mononuclear Cells (PBMC) and tumor infiltrating lymphocytes (TIL), either directly from FFPE tumor sections, or following isolation, will be used for the TCR repertoire analysis by next-generation sequencing. Intratumoral response to CSF-1R inhibition will be measured by IHC of monocytes/macrophages on the FFPE slides of pre- and post-treatment biopsy specimens, and measured by using ELISA to measure serum CSF1 levels.

Results from the sequencing studies will not be released to the patients. These studies are for research purposes only and are not using a clinically validated platform.

8.1.1 Immunohistochemistry

Immunohistochemistry of immune parameters relevant to the PD-L1/PD-1 pathway will be performed on FFPE slides of pre-treatment core biopsy and post- treatment surgically resected PDA specimens. The IHC protocols were previously established. We will examine the densities and distribution of effector T cells (Teff) including CD8, CD45RO, CXCR3, CD68+ and CD69+ cells, expression of immune suppressive signals including PD-1, PD-L1, PD-L2, LAG3, TIM3, and IDO1, immune activation signals including CD137(4-1BB), 4-1BBL, OX40, OX40L, CD40, and CD40L on T cells and macrophages, and the expression of T helper cell differentiation markers including Tbet (for Th1), GATA3 (for Th2), RORγT (for Th17), and Foxp3 (for Treg) as previously described.[14] Quantification of IHC on immune markers will be conducted by Aperio's Immune Analysis Software Program as described previously[14] and will be supervised by our pathologist (Dr. Robert Anders). We will particularly examine whether the combination of GVAX, anti-PD-1 therapy, and CSF1R inhibitor will collaboratively suppress the Treg pathway as our preclinical study in the mouse model showed that GVAX and anti-PD-1 antibody were able to collaboratively bring down the increased infiltration of Treg induced by either GVAX or anti-PD1-antibody.[22]

8.1.2 Gene Expression Assays

Quantitative gene expression assays such as nanostring assays and cytokine arrays will be used to assess whether the combination of anti-PD-1 therapy and CSF-1R inhibition tips the balance between Th1 vs. Th2, M1 vs. M2, Teff vs. Treg within tumor microenvironment. Tissues derived from FFPE sections of biopsy specimens will also be subject to the analysis of Merck's nanostring panels in collaboration with Merck's scientists (Shankaran et al. ASCO GI 2015). We will particularly focus on the genes expression signatures listed in **Table 8** and the signature founded to correlate with responses to the pembrolizumab treatment. In addition, fresh frozen biopsy specimens will be subjected to the cytokine array analysis by a well-established protocol in the Zheng Lab using a commercially available cytokine protein array (R&D). We will particularly focus on the Th1 vs. Th2 cytokines, M1 vs. M2 cytokines and the chemokines that are involved in T cell trafficking.

8.1.3 T cell receptor repertoire analysis

Tumor antigen-specific T cells that traffic into the tumor are the most relevant T cells to study when evaluating antitumor immune responses. We will examine the TCR repertoire in PBMC and TIL from FFPE sections, using next-generation sequencing. We will compare TCR repertoires in pre-treatment vs. post-treatment PBMC and pre-treatment biopsy specimens vs. post-treatment

specimens from the same patients to identify TCR's expressed by T cells that expand following treatment. We will also compare TCR repertoires in PBMC vs TIL from the same patients to identify any T cell clones that are induced or expanded in PBMC by vaccine+ Pembro+CSF1R inhibitor and also enriched in TIL. We will use these data to determine if T cells in PBMC and TIL undergo clonal expansion following GVAX+Pembro+CSF1R inhibitor treatment, and if so, whether the vaccine-expanded T cell clones traffic to the PDA TME.

Table 8. Immune-Related Gene Expression Signatures (Shankaran et al. ASCO GI 2015)

IFN γ	Expanded Immune	TCR Signaling	De Novo
IDO1<br>CXCL10<br>CXCL9<br>HLA-DRA<br>STAT1<br>IFNG	CD3D NKG7<br>IDO1 HLA-E<br>CIITA CXCR6<br>CD3E LAG3<br>CCL5 TAGAP<br>GZMK CXCL10<br>CD2 STAT1<br>HLA-DRA GZMB<br>CXCL13<br>IL2RG	CD27<br>TIGIT<br>CD8A<br>CD3D<br>GRAP2<br>LCK<br>PTPRCAP<br>CD4<br>CCL5<br>IL2RB IKZF3<br>CD3G CD74	IKZF3 SAMHD1 CD38<br>HLA-DPB1 TIGIT CRTAM<br>CD27 IL2RB CD8A<br>AMICA1 TARP CXCL9<br>CD74 CD3D HLA-C<br>LY9 CD3G GPR18<br>CD4 HLA-B IL18<br>HLA-DRA IGJ CX3CR1<br>B2M IRF1 CXCL10<br>IGSF6 BST2 SIT1<br>FASLG PTPN7 LCK

IFN = interferon; TCR = T-cell receptor.

8.2 Serum and Plasma Marker Studies

Sera (up to 5 cc) and plasma (up to 20cc) will be collected at the specified timepoints (See **Section 9**) to identify potential therapeutic targets, biomarkers, and predictors of response and autoimmune toxicity through proteomic approaches. ELISA will also be used to measure serum CSF1 levels. Detailed instructions for blood collection, processing, and storage are provided in the Laboratory Manual.

8.3 Diagnostic Tissue Samples

Tissue, fluid, or blood may be collected from standard of care procedures used to treat or diagnoses immune related toxicities.

8.4 Genomic Analysis

Genomic sequencing library construction, whole genome/exome sequencing, whole transcriptome sequencing, microbial sequencing, neoepitope prediction, mutation burden, and bioinformatic analysis will be performed either at an on-campus laboratory or at an off-campus sequencing service. All the samples will be de-identified before

sending to any laboratory for sequencing. The FASTQ files, BAM files and VCF files will be generated and analyzed.

Results from the sequencing studies will not be released to the patients. These studies are for research purposes only and are not using a clinically validated platform.

9. STUDY SCHEDULE

9.1 Study Schedule Prior to Surgery

Subjects will be screened after completing neoadjuvant therapy for resectable or borderline resectable pancreatic cancer. Treatment should start no later than 28 days of last dose of neoadjuvant therapy.

Study Schedule Prior to Surgery<br><br>Procedure	Screening visit (7 days from last dose of neoadjuvant therapy[1]	Cycle 1 Immunotherapy[2] (7-28 days from last dose of neoadjuvant therapy)				Cycle 2 Immunotherapy (3 weeks after Immunotherapy #1)				Post Immunotherapy Evaluation (7-14 days after Immunotherapy#2)[17]
		C1 D1	C1 D2	C1 D8	C1 D15	C2 D1	C2 D2	C2 D8	C2 D15	
Visit Window[3]		-	-	-	-	+7	-	-	-	+7
Cyclophosphamide		X				X				
GVAX			X				X			
Pembrolizumab		X				X				
IMC-CS4		X		X	X	X		X	X	
Informed consent	X									
Inclusion/exclusion criteria	X									
Demographics	X									
Medical History, Medications[4]	X									
Physical Exam[5]	X	X				X				X
Vital signs and pulse ox[6]	X	X	X	X	X	X	X	X	X	X
Height[6]	X									
Weight[6]	X	X				X				X
Performance Status	X	X				X				X
Hematology profile[7,8]	X	X		X	X	X		X	X	X
Chemistry profile[8,9]	X	X		X	X	X		X	X	X
TSH[8,10]		X				X				X
CK	X	X				X				X
Serum or Urine Pregnancy test[11]	X	X[11]				X				
CA 19-9[8]	X									X
Urinalysis and microscopic exam[8,12]	X									
INR and pTT[8]	X									X
Adverse event evaluation[5]			X	X	X	X	X	X	X	X
Vaccine Site Assessment						X				X
PET-CT, CT, or MRI[13]	X									X
Tumor measurements[13]	X									X
Pathology Review	X									X
Peripheral blood for PBMC (up to 120cc)[14,18]		X								X

Study Schedule Prior to Surgery<br><br>Procedure	Screening visit (7 days from last dose of neoadjuvant therapy[1]	Cycle 1 Immunotherapy[2] (7-28 days from last dose of neoadjuvant therapy)				Cycle 2 Immunotherapy (3 weeks after Immunotherapy #1)				Post Immunotherapy Evaluation (7-14 days after Immunotherapy#2)[17]
		C1 D1	C1 D2	C1 D8	C1 D15	C2 D1	C2 D2	C2 D8	C2 D15	
Peripheral blood for plasma (up to 20cc)[14,18]		X								X
Peripheral blood for CTC (up to 20 cc)[14,18]		X								X
Serum (up to 5cc)[14,18]		X								X
EUS Core Biopsy[18]	X[15]									X[16]
Surgical Evaluation	X									X

In order to minimize the need for research-only in-person visits, telemedicine visits may be substituted for inperson clinical trial visits or portions of clinical trial visits where determined to be appropriate and where determined by the investigator not to increase the participants risks. Prior to initiating telemedicine for study visits the study team will explain to the participant, what a telemedicine visit entails and confirm that the study participant is in agreement and able to proceed with this method. Telemedicine acknowledgement will be obtained in accordance with the Guidance for Use of Telemedicine in Research. In the event telemedicine is not deemed feasible, the study visit will proceed as an in-person visit. Telemedicine visits will be conducted using HIPAA compliant method approved by the Health System and within licensing restrictions.

1. Potential candidates will be identified when they are receiving neoadjuvant therapy.
2. Each immunotherapy consists of a combination of CY, GVAX, pembrolizumab, and IMC-CS4
3. Longer delays to be approved by the IND sponsor
4. Includes history of lung disease, HIV, hepatitis B or C infection, and complete cancer history, including primary site of cancer, gross location of primary tumor, histology, histologic grade, date of initial diagnosis, date of metastatic diagnosis, prior cancer therapy regimens
5. Complete physical exam will be completed at baseline; focused physical examinations will be conducted thereafter. Physical exams and AE assessments can be made up to 3 days prior to infusion.
6. Blood pressure, pulse, respiratory rate, and temperature will be collected at baseline, prior to CY, pembrolizumab, and IMC-CS4 infusions, prior to and after GVAX pancreas vaccine administration, and during the post immunotherapy evaluation. Height will be taken at or prior to screening only. Weight will be obtained at baseline, prior to each cycle, and during the post immunotherapy evaluation. Pulse oximetry will be obtained at baseline and prior to dosing on Days 1, 8, and 15 of each cycle.
7. CBC with differential including absolute eosinophil count, absolute neutrophils, absolute lymphocytes, and platelets

8. Labs may be collected within a window of up to 4 days prior to dosing. Unexpected Grade 3 or greater laboratory abnormalities should be repeated within 24-72 hours if clinically indicated and monitored as necessary to determine if event meets toxicity criteria.
9. Albumin, alkaline phosphatase, total bilirubin, bicarbonate, BUN, calcium, chloride, creatinine, glucose, potassium, total protein, SGOT [AST], SGPT [ALT], sodium, CK, LDH, CRP.
10. FT3 and FT4 to be checked reflexively if TSH is abnormal
11. For WOCBP. Pregnancy tests must be done within 7 days of the first treatment and then will be repeated with each subsequent cycle (collected within a window of up to 3 days prior to dosing).
12. Bilirubin, blood, glucose, ketones, leukocytes, nitrite, pH, color, protein, RBC and WBC count, and specific gravity
13. Radiologic evaluations (CT pancreas protocol, chest, abdomen, and pelvis with contrast) and tumor measurements will be performed at baseline (within 28 days prior to the first study drug dose) and 7 to 14 days after completion of cycle 2 of combination immunotherapy (iRECIST per **Appendix D**). Non-contrast CT Chest and MRI Abdomen/pelvis will be done for those with contrast allergies.
14. Detailed instructions for blood collection, processing, and storage are provided in the Laboratory Manual. **OPTIONAL**: At the discretion of the PI, 20 cc of whole blood may be collected for CTC as described in the Laboratory Manual.
15. Endoscopy will be scheduled for obtaining 4-6 core biopsies at baseline. Biopsy should be obtained within 14 days prior to first dose of study drug.
16. If subject is a surgical candidate, biopsy will be obtained during surgical resection. If metastases are noted, an optional biopsy of metastases for research purposes will be performed.
17. Window 7 days +/- 7 days
18. Research samples will be collected at the discretion of the PI based on availability of supplies and safety of patient and staff.

9.2 Study Schedule After Surgery/ Adjuvant Chemotherapy

After completing neoadjuvant therapy, subjects will be evaluated for surgical resectability at multidisciplinary tumor board (based upon clinical health status and resectability as per NCCN guidelines) and presence of distant metastatic disease

- If subjects are deemed to have developed resectable tumors, they will undergo traditional surgical resection.

- If a subject is noted to have developed metastases to the liver or other metastases that can be safely biopsied, optional biopsy of the metastases will be obtained

Subjects without metastatic disease will have the option of receiving adjuvant chemotherapy within 4-8 weeks postoperatively, at the discretion of their primary oncologist.

After adjuvant chemotherapy completion or after surgery if the subject does not receive adjuvant chemotherapy, subjects will then undergo repeat examination and further immunotherapy as per the below schedule:

Study Schedule After Surgery/ Adjuvant Chemotherapy

Procedure	Post-surgery/chemo-therapy evaluation (<=28 days before C3D1)	Combination immunotherapy[1] on 3-week cycles[2] (=>28 days after standard adjuvant chemotherapy)															
		C3 D1	C3 D2	C3 D8	C3 D15	C4 D1	C4 D2	C4 D8	C4 D15	C5 D1	C5 D2	C5 D8	C5 D15	C6 D1	C6 D2	C6 D8	C6 D15
Visit Window[3]	-	+7	-	-	-	+7	-	-	-	+7	-	-	-	+7	-	-	-
Cyclophosphamide		X				X				X				X			
GVAX			X				X				X				X		
Pembrolizumab		X				X				X				X			
IMC-CS4		X		X	X	X		X	X	X		X	X	X		X	X
Physical Exam[4]	X	X				X				X				X			
Vital signs and pulse ox[5]	X	X	X	X	X	X	X	X	X	X	X	X	X	X	X	X	X
Weight[5]	X	X				X				X				X			
Performance Status	X					X				X				X			
Hematology profile[6,7]	X	X		X	X	X		X	X	X		X	X	X		X	X
Chemistry profile[7,8]	X	X		X	X	X		X	X	X		X	X	X		X	X
TSH[7,9]	X	X				X				X				X			
Serum or Urine Pregnancy[10]	X	X				X				X				X			
CA 19-9[7]	X									X							
CK	X	X				X				X				X			
Adverse event evaluation[4]	X[13]	X	X	X	X	X	X	X	X	X	X	X	X	X	X	X	X
Vaccine Site Assessment		X				X				X				X			
Con meds		X				X				X				X			
CT or MRI[11]	X									X							
Tumor measurements[11]	X									X							
Peripheral blood for PBMC (up to 120cc)[12,14]		X								X							
Peripheral blood for plasma (up to 20cc)[12,14]		X								X							
Peripheral blood for CTC (up to 20cc)[12,14]		X								X							
Serum (Up to 5 mL)[12,14]		X								X							

In order to minimize the need for research-only in-person visits, telemedicine visits may be substituted for inperson clinical trial visits or portions of clinical trial visits where determined to be appropriate and where determined by the investigator not to increase the participants risks. Prior to initiating telemedicine for study visits the study team will explain to the participant, what a telemedicine visit entails and confirm that the study participant is in agreement and able to proceed with this method. Telemedicine acknowledgement will be obtained in accordance with the Guidance for Use of Telemedicine in Research. In the event telemedicine is not deemed feasible, the study visit will proceed as an in-person visit. Telemedicine visits will be conducted using HIPAA compliant method approved by the Health System and within licensing restrictions.

1. Each immunotherapy consists of a combination of CY, GVAX, pembrolizumab, and IMC-CS4.
2. Subjects will receive combination immunotherapy on a 21-day cycle. If subjects develop irAE or toxicities precluding further pembrolizumab or IMC-CS4 administration, they will still be eligible to continue with CY/GVAX monotherapy to complete the booster treatment or until development of distant metastases. Unexpected Grade 3 or greater laboratory abnormalities should be repeated within 24-72 hours if clinically indicated and monitored as necessary to determine if event meets toxicity criteria.
3. Longer delays to be approved by the IND Sponsor.
4. Focused physical examinations will be conducted. Physical exams and AE assessments can be made up to 3 days prior to infusion.
5. Blood pressure, pulse, respiratory rate, and temperature will be collected during the post-chemotherapy evaluation, prior to CY, pembrolizumab, and IMC-CS4 infusions, and prior to and after GVAX pancreas vaccine administration. Weight will be obtained during the post-chemotherapy evaluation and prior to each cycle. Pulse oximetry will be obtained prior to dosing on Days 1, 8, and 15 of each cycle.
6. CBC with differential including absolute eosinophil count, absolute neutrophils, absolute lymphocytes, and platelets.
7. Labs may be collected within a window of up to 4 days prior to dosing. Unexpected Grade 3 or greater laboratory abnormalities should be repeated within 24-72 hours if clinically indicated and monitored as necessary to determine if event meets toxicity criteria.
8. Albumin, alkaline phosphatase, total bilirubin, bicarbonate, BUN, calcium, chloride, creatinine, glucose, potassium, total protein, SGOT [AST], SGPT [ALT], sodium, CK, LDH, CRP.
9. FT3 and FT4 to be checked reflexively if TSH is abnormal.
10. For WOCBP. Pregnancy tests must be repeated with each subsequent therapy (collected within a window of up to 3 days prior to dosing).
11. Radiologic evaluations (CT pancreas protocol, chest, abdomen, and pelvis with contrast) and tumor measurements will be performed. Non-contrast CT Chest and MRI Abdomen/pelvis will be done for those with contrast allergies. Cycle 5 scans may be done up to 7 days prior to the infusion.
12. Detailed instructions for blood collection, processing, and storage are provided in the Laboratory Manual. **OPTIONAL**: At the discretion of the PI, 20 cc of whole blood may be collected for CTC as described in the Laboratory Manual.
13. See **Section 6.6** for the surgical toxicities that will be collected.

14. Research samples will be collected at the discretion of the PI based on availability of supplies and safety of patient and staff.

9.3 Study Schedule for Booster Study Immunotherapy

Following the 4 adjuvant study therapy cycles, as long as the patient remains disease free and more than 90 days have not elapsed since the last immunotherapy treatment, the patients will receive 12 booster cycles of pembrolizumab every 3 weeks, and 2 booster cycles of Cy/GVAX given every 18 weeks. The first booster Cy/GVAX will be given when receiving the 6th booster dose of pembrolizumab (cycle 12), and the second booster Cy/GVAX will be given when receiving the 12th booster dose of pembrolizumab (cycle 18).

Study Schedule for Booster Immunotherapy

Procedure	Booster Pembrolizumab on 3-week cycle and Cy/GVAX on 18-week cycle														Off Study[1]
	C7 D1	C8 D1	C9 D1	C10 D1	C11 D1	C12 D1	C12 D2	C13 D1	C14 D1	C15 D1	C16 D1	C17 D1	C18 D1	C18 D2	
Visit Window[12]	+7	+7	+7	+7	+7	+7	+7	+7	+7	+7	+7	+7	+7	+7	+7
Cyclophosphamide						X							X		
GVAX							X							X	
Pembrolizumab	X	X	X	X	X	X		X	X	X	X	X	X		
Physical Exam[11]	X	X	X	X	X	X		X	X	X	X	X	X		X
Vital Signs and pulse ox[2]	X	X	X	X	X	X	X	X	X	X	X	X	X	X	X
Weight	X	X	X	X	X	X		X	X	X	X	X	X		X
Performance Status	X	X	X	X	X	X		X	X	X	X	X	X		X
Hematology profile[3,4]	X	X	X	X	X	X		X	X	X	X	X	X		X
Chemistry profile[4,5]	X	X	X	X	X	X		X	X	X	X	X	X		X
TSH[4,6]	X	X	X	X	X	X		X	X	X	X	X	X		X
Serum or Urine Pregnancy[4,7]	X	X	X	X	X	X		X	X	X	X	X	X		
CA 19-9[4]	X					X							X		X
Adverse event evaluation	X	X	X	X	X	X		X	X	X	X	X	X		X
Vaccine Site Assessment						X							X		X
Con meds	X	X	X	X	X	X		X	X	X	X	X	X		X
CT or MRI[8]	X					X							X		X
Tumor measurements[8]	X					X							X		X
Peripheral blood for PBMC (up to 120cc)[9,10]	X					X							X		X
Peripheral blood for plasma (up to 20cc)[9,10]	X					X							X		X
Peripheral blood for CTC (up to 20cc)[9,10]	X					X							X		X
Serum (up to 5cc)[9.10]	X					X							X		X

In order to minimize the need for research-only in-person visits, telemedicine visits may be substituted for inperson clinical trial visits or portions of clinical trial visits where determined to be appropriate and where determined by the investigator not to increase the participants risks. Prior to initiating telemedicine for study visits the study team will explain to the participant, what a telemedicine visit entails and confirm that the study participant is in agreement and able to proceed with this method. Telemedicine acknowledgement will be obtained in accordance with the Guidance for Use of Telemedicine in Research. In the event telemedicine is not deemed feasible, the study visit will proceed as an in-person visit. Telemedicine visits will be conducted using HIPAA compliant method approved by the Health System and within licensing restrictions.

1. 30 days after the last dose of study drug or within 7 days prior to initiation of a new anti-cancer treatment, whichever comes first. If the off study visit occurs early, scans do not need to be repeated if one has been done within the past 6 weeks. Patients who discontinue from treatment should be contacted (by phone or email) every six months (+/- 1 month) for up to 24 months or study closure to monitor overall survival. Information of other cancer therapies after discontinuation from the study treatment will be collected. The off study scan does not need to be repeated if one has been performed within 6 weeks of the visit.
2. Blood pressure, pulse, respiratory rate, and temperature will be collected prior to CY and pembrolizumab infusions, prior to and after GVAX pancreas vaccine administration, and during the off study evaluation. Weight will be obtained prior to each cycle and during the off study evaluation. Pulse oximetry will be obtained prior to dosing on Day 1 of each cycle.
3. CBC with differential including absolute eosinophil count, absolute neutrophils, absolute lymphocytes, and platelets.
4. Labs may be collected within a window of up to 4 days prior to dosing. Unexpected Grade 3 or greater laboratory abnormalities should be repeated within 24-72 hours if clinically indicated and monitored as necessary to determine if event meets toxicity criteria.
5. Albumin, alkaline phosphatase, total bilirubin, bicarbonate, BUN, calcium, chloride, creatinine, glucose, potassium, total protein, SGOT [AST], SGPT [ALT], sodium.
6. FT3 and FT4 to be checked reflexively if TSH is abnormal.
7. For WOCBP. Pregnancy tests must be done within 7 days of the first treatment and then will be repeated with each subsequent therapy (collected within a window of up to 3 days prior to dosing).
8. Radiologic evaluations (CT pancreas protocol, chest, abdomen, and pelvis with contrast) and tumor measurements will be performed. Non-contrast CT Chest and MRI Abdomen/pelvis will be done for those with contrast allergies. Cycle 7, 12, and 18 scans may be done up to 7 days prior to the infusion.
9. Detailed instructions for blood collection, processing, and storage are provided in the Laboratory Manual. **OPTIONA**L: At the discretion of the PI, 20 cc of whole blood may be collected for CTC as described in the Laboratory Manual.
10. Research samples will be collected at the discretion of the PI based on availability of supplies and safety of patient and staff.
11. Physical exams and AE assessments can be made up to 3 days prior to infusion.
12. Window for C7-C18 is -3 days/+7 days.

10. MEASUREMENT OF EFFECT

10.1 Antitumor Effect – Solid Tumors

10.1.1 Definitions

Evaluable for toxicity. All subjects are evaluable for toxicity after receiving first dose of combined immunotherapy.

Evaluable for objective response. All patients who have received at least one dose of neoadjuvant immunotherapy (i.e. prior to surgery) and have had their disease re-evaluated with imaging prior to surgery will be considered evaluable for response. Response criteria will be classified by immune-related RECIST (irRC) criteria (**Appendix D)**

10.1.2 Methods for Evaluation of Measurable Disease

All measurements should be taken and recorded in metric notation using a ruler or calipers. All baseline evaluations should be performed as closely as possible to the beginning of treatment and never more than 28 days before the beginning of the treatment. Subjects will be evaluated for anti-tumor effect by follow-up imaging (pancreas protocol CT, CT Chest/Abd/Pelvis, PET-CT imaging, and/or non-contrast CT chest and MRI Abd/pelvis) as outlined above. All subsequent scans (post-treatment) will be compared to the same pretreatment CT, PET/CT, or MRI that was used prior to initiating of study treatment.

The same method of assessment and the same technique should be used to characterize each identified and reported lesion at baseline and during follow-up. Imaging-based evaluation is preferred to evaluation by clinical examination unless the lesion(s) being followed cannot be imaged but are assessable by clinical exam.

Conventional CT and MRI: This guideline has defined measurability of lesions on CT scan based on the assumption that CT slice thickness is 5 mm or less. If CT scans have slice thickness greater than 5 mm, the minimum size for a measurable lesion should be twice the slice thickness. MRI is also acceptable in certain situations (e.g. for body scans).

Use of MRI remains a complex issue. MRI has excellent contrast, spatial, and temporal resolution; however, there are many image acquisition variables involved in MRI, which greatly impact image quality, lesion conspicuity, and measurement. Furthermore, the availability of MRI is variable globally. As with CT, if an MRI is performed, the technical specifications of the scanning sequences used should be optimized for the evaluation of the type and site of disease. Furthermore, as with CT, the modality used at follow-up should be the

same as was used at baseline and the lesions should be measured/assessed on the same pulse sequence. It is beyond the scope of the immune-related RECIST guidelines to prescribe specific MRI pulse sequence parameters for all scanners, body parts, and diseases. Ideally, the same type of scanner should be used and the image acquisition protocol should be followed as closely as possible to prior scans. Body scans should be performed with breath-hold scanning techniques, if possible.

10.1.3 Disease free survival (DFS)

DFS is defined as the duration of time from start of treatment to identification of local or metastatic disease recurrence on imaging. Individuals will be censored at the date of the last scan if no event occurs.

10.1.4 Overall Survival (OS)

OS is defined as the duration of time from start of study treatment to time of death. Individuals will be censored at the date of the last scan if no event occurs.

10.1.5 Overall Response Rate (ORR)

Overall response rate as defined by the sum of complete response and partial response rate.

10.1.6 Surgical Resectability Rate

Surgical resectability after neoadjuvant therapy as determined by our pancreatic multidisciplinary group based upon NCCN guidelines for resectability.

10.1.7 Pathologic Response Rate

Pathologic response as determined by and the presence of residual disease in surgical specimen.

11. DATA REPORTING / REGULATORY REQUIREMENTS

Adverse event guidelines and instructions for AE reporting can be found in **Section 6 (Adverse Events: List and Reporting Requirements)**.

11.1 Data Management

All information will be collected on study-specific case report forms (CRFs) by study staff. These data will be reviewed for completeness and accuracy by the Principal Investigator.

11.2 Safety Meetings

Scheduled meetings will occur on a regular basis with the frequency dependent on the rate of subject accrual and the progress of the study. Meetings will include the protocol principal investigator, study coordinator(s), research nurses(s), sub-investigators (as appropriate), collaborators (as appropriate), and biostatisticians (as appropriate) involved with the conduct of the protocol. During these meetings matters related to the following will be discussed: safety of protocol participants, validity and integrity of the data, enrollment rate relative to expectation, characteristics of participants, retention of participants, adherence to protocol (potential or real protocol violations), data completeness, and progress of data for objectives.

Monthly teleconferences will be scheduled to include the Investigator and Merck representatives. During these meetings, the Investigator shall provide Merck with study progress updates. The Investigator will provide a summary of key points from the weekly meetings with a focus on safety of the protocol participants, enrollment status, and progress of data for objectives. In addition, Merck will provide safety and applicable program updates to the IND Sponsor.

11.3 Monitoring

The SKCCC Compliance Monitoring Program will provide external monitoring for JHU-affiliated sites in accordance with SKCCC DSMP (Version 6.0, 02/21/2019). The SMC Subcommittee will determine the level of patient safety risk and level/frequency of monitoring. Data monitoring of this protocol will occur on a regular basis with the frequency dependent on the rate of subject accrual and the progress of the study. The protocol will be monitored internally by the Principal Investigator. The protocol will be monitored externally by the SKCCC CRO in accordance with SKCCC guidelines. Additional data and safety monitoring oversight will also be performed by the SKCCC Safety Monitoring Committee (SMC - as defined in the DSMP) and a Medical Expert Committee (MEC) as detailed below.

The Medical Expert Committee (MEC) for this clinical study contains three medical oncologists from other disciplines who are not affiliated with this clinical trial protocol. The MEC will review safety data on at least a semi-annual basis. The MEC will provide a written summary of each assessment to the IND Sponsor after each meeting. In turn, the study team will forward these summaries to the JHU and other participating site IRBs and JHU SKCCC SMC. The operating plan of the MEC will be as follows:

- Meetings will be held at least semi-annually, and potentially more frequently if needed.
- Meetings will be conducted in-person or via video/teleconference, with a participant sign-in sheet collected at each meeting.
- Approximately one week prior to each MEC meeting, the study team will submit the following items to MEC personnel for review and discussion at the meeting

(The PI may join the MEC meeting in order to answer any questions the MEC might have):

- o A summary of the clinical trial's progress to date;
- o The latest IRB-approved consent document;
- o A summary of all adverse events, serious adverse events, deaths, and withdrawals to date;

Note that the MEC reserves the right to halt trial accrual or all study activity if, after review, serious safety concerns warrant this action. If the MEC halts study accrual or all study activity, then the study team must notify the JHU SKCCC SMC, JHU IRB, JHU IBC, RAC, and the FDA immediately.

Dr. Jaffee will be holding the IND for this study. She will comply with all regulated reporting requirements to the FDA.

12. STATISTICAL CONSIDERATIONS

12.1 Sample Size/Accrual Rate

This pilot study aims to evaluate safety and efficacy of the triple combination of CY/GVAX, pembrolizumab, and CSF-1R inhibitor (IMC-CS4) in 8 evaluable patients with resectable or borderline resectable pancreatic cancer (BRPC) following induction chemotherapy (with or without radiation) given before and after surgical resection. Toxicities are monitored and graded per CTCAE v4.0. Safety analyses will be based on all enrolled subjects who received at least one dose of any study treatment, including those who were found intraoperatively to be unresectable after the study treatment initiation. Participants in the current study will be considered evaluable if they have completed at least one dose of combination immunotherapy, have underwent an R0, R1 or R2 surgical resection of PDA and have adequate tissue specimen for immunologic endpoints (pre-treatment core biopsy and surgical specimen). Even if there is a dose-reduction or an omission of a study drug (e.g. pembrolizumab, Cy/GVAX or IMC-CS4), as long as the subject as received one dose of the combination of immunotherapy at doses specified in **Table 1** in **Section 4.1** and underwent an R0, R1 or R2 surgical resection, then the patient is evaluable for the primary immunology endpoint. Analyses of efficacy endpoints will be based on evaluable patients.

We expect that approximately 30% of patients who may be found intraoperatively to be unresectable after the study treatment initiation. We expect to obtain 8 evaluable patients with R0, R1 or R2 resection of their tumors. At the individual patient level, a significant treatment-related immunologic effect (i.e immune response) in tumor infiltrating CD8 density is defined as a >80% increase or decrease (post vs pre-surgery) AND such increase/decrease is at least 1.8 times the baseline median absolute deviation (MAD), where the baseline MAD is defined as the median of the absolute deviation from the median calculated across patients and is thus a more robust measure of the variability than sample standard deviation. The treatment will be judged as yielding a

promising biologic effect if at least 3 of 8 patients demonstrate an immune response. In this case, the rate of immune response is 38% and the corresponding binomial exact 90% confidence interval is (0.11, 0.71). In other words, we will be able to claim the immune response rate in terms of significant change in tumor infiltrating CD8 density is significantly higher than 10% at a 10% Type I error rate.

Each year, approximately 250 patients with newly diagnosed locally advanced pancreatic cancer are seen at JHH. Approximately, 50% of these patients will potentially be enrolled in clinical trials. We estimate that we could complete the accrual goal within 9-12 months.

12.2 Early Stopping Boundaries for Toxicity

This pilot study aims to evaluate safety and immune efficacy of the triple combination of CY/GVAX, pembrolizumab, and CSF-1R inhibitor (IMC-CS4) in patients with resectable or borderline resectable pancreatic cancer (BRPC) following induction chemotherapy (with or without radiation) given before and after surgical resection. Toxicities are monitored and graded per CTCAE v4.0. Safety analyses will be based on all enrolled subjects who received at least one dose of any study treatment, including those who were found intraoperatively to be unresectable after the study treatment initiation.

The first three patients in Phase One of this study will be monitored closely for toxicities (particularly grade 3 or 4 LFT abnormalities) and potential immune-related adverse events. If more than one patient developed grade 3 or 4, the study be halted for a safety consultation. Safety in the rest 9 patients in Phase Two will be monitored by a Bayesian stopping rule based on the rate of Grade 3/4 toxicities. The triple combination of GVAX, pembrolizumab, and IMC-CS4 will be considered feasible and safety if the risk of Grade 3/4 toxicities is lower than 30%. We will apply a Bayesian toxicity monitoring rule that suspends the enrollment if the posterior probability of risk being larger than that threshold is 75% or higher. According to the investigator's brochure, 9.5% of subjects experience grade 3 and higher toxicities with single-agent pembrolizumab 10 mg/kg administered every 2 weeks, so we expect increased toxicity from this study's combination immunotherapy, but we do not expect grade 3 and higher toxicities to exceed 20%. We assume *a priori* that this triple combination has a rate of Grade 3/4 toxicities around 20% and that there is about a 24% chance that the rate is 30% or higher. This corresponds to a Beta (1,4) prior distribution. The stopping rule will hold enrollment if the posterior probability of toxicity risk exceeding 30% is 70% or higher. **Table 9** gives the corresponding stopping rules. For example, if 4 patients out of the first 5-7 patients experience unacceptable toxicities (as defined in **Section 4.5**), accrual to the trial will be temporarily halted. The principal investigator, the study team, and the IND sponsor will review the toxicity data and recommend either modification or termination of the trial.

Table 9: The number of toxicities needed in Phase Two to trigger stopping guidelines throughout the course of the study

Number of Subjects	Number of toxicities needed to trigger re-evaluation
2	2
3-5	3
6-8	4
9	5

12.3 Analysis of Primary Safety Endpoints

Safety analyses will be based on all enrolled subjects who received at least one dose of any study treatment, including those who were found intraoperatively to be unresectable after the study treatment initiation. Subjects who discontinue prior to their first dose of study drug will be replaced and will not be included in the primary safety analysis. A complete list of all AE data will be provided along with an assessment of NCI CTCAE grade and relationship to study drug. The proportion of patients with Grade 3/4 toxicities will be reported, together with the corresponding binomial exact 95% confidence intervals. The incidence of AEs will be tabulated by subgroups of interest (e.g. grade 3 or higher, organ class, relationship to study drug). For analyses at the individual level, the highest grade and relationship to study drug will be assumed if multiple events have occurred. Toxicity will be tabulated by type and grade and will be summarized with descriptive statistics. Other safety data will be assessed in terms of physical examination, clinical chemistry, hematology, vital signs, and ECGs. A baseline measurement and at least 1 laboratory or other safety-related measurement obtained after at least 1 dose of study treatment may be required for inclusion in the analysis of these safety parameter (e.g., lab shifts from baseline).

Surgical complications will be defined based on Clavien-Dindo classification.[45] The monitoring rule will focus on Grade IIIa surgical complication or above, which is beyond what may be expected for chemotherapy or resection without immunotherapy, and that may be attributable to these drugs (any component of Cy/GVAX/pembrolizumab/ and CSF-1R inhibitor). If the risk of Grade IIIa or higher surgical complications appears to be greater than 40%, the study will temporarily be halted pending feasibility evaluations. The study may resume after discussion between the principal investigator and the IND sponsor if the surgical co-investigators do not deem there is an association of an increased rate of high grade postoperative complication with any of the study treatments. Specifically, we apply a Bayesian monitoring rule that suspends the enrollment if the posterior probability of risk being greater than 40% is 0.5 or higher. The previous study showed 26% Grade IIIa or above post-operative complications in patients receiving neoadjuvant stereotactic body radiation therapy or chemoradiation therapy. [46] Thus, a Beta (2.5, 5.5) prior, representing the prior guess of a post-operative complications (Grade IIIa or above) rate of 31%, will be used. Surgical complications will be monitored continuously

according to the protocol amendment. **Table 10** shows the number of post-operative complications (Grade IIIa or above) that would need to be observed in order to trigger the stopping guidelines throughout the course of the trial.

Table 10. The number of post-operative complications (Grade IIIa or above) needed to trigger stopping guidelines throughout the course of the study.

Number of Patients	Number of post-operative complications (Grade IIIa or above) needed to trigger re-evaluation
2-3	2
4-5	3
6-8	4
9-10	5
11-12	6

The probability of trigger the stopping was assessed for a range of true underlying post-operative complications (Grade IIIa or above) rates using simulations with 5,000 replicates (**Table 11**). The probability of stopping is 22.5% if the true proportion with post-operative complications (Grade IIIa or above) rate was 25%. In contrast, the probability of early stopping is 56.8% if the true proportion with post-operative complications (Grade IIIa or above) was 40%, the threshold for acceptable levels of post-operative complications (Grade IIIa or above).

Table 11. The operating characteristics of the stopping rule based on 5000 simulations.

True rate of post-operative complications (Grade IIIa or above)	% time study stops	Expected total sample size
10%	3	11.7
15%	8.2	11.3
20%	15.2	10.8
25%	22.5	10.2
30%	34.1	9.4
35%	46.4	8.4
40%	56.8	7.7
45%	68.7	6.8
50%	78.2	5.9
60%	91.9	4.5

12.4 Analysis of Primary Immunology Endpoints

The primary immunology endpoint is tumor infiltrating CD8 density. Other intratumoral immune parameters include but not limited to tumor-associated macrophages (TAM), effector T cell density, and vaccine-induced lymphoid aggregates. The evaluable population for the analysis of primary immunology endpoints includes all subjects who have completed at least one dose of combination immunotherapy and have underwent an R0, R1 or R2 surgical resection. Even if there is a dose-reduction or an omission of a study drug (e.g. pembrolizumab, Cy/GVAX or IMC-CS4), as long as the subject as received one dose of the combination of immunotherapy at doses specified in **Table 1** in **Section 4.1** and underwent an R0, R1 or R2 surgical resection, then the patient is evaluable for the primary immunology endpoint. Subjects who discontinue prior to their first dose of study drug will be replaced and will not be included in the primary efficacy analysis.

Descriptive statistics for primary immune parameters in pretreatment tumor biopsy specimens and that in matched posttreatment resected tumors will be computed. Percent change in the intratumoral immune parameters will be reported using descriptive statistics and displayed graphically. Comparison of primary immune parameters between pretreatment tumor biopsy specimens and matched posttreatment resected tumors will be conducted using paired t-tests (or Wilcoxon signed rank tests if appropriate) and McNemar's tests for dichotomous or categorical variables, respectively. At the individual patient level, a significant treatment-related immunologic effect is defined as a >80% increase or decrease (post vs pre-surgery) in a specific immune parameter AND such increase/decrease is at least 1.8 times the baseline median absolute deviation (MAD). The proportion of immune response for each intratumoral immune parameter will be reported along with its corresponding binomial exact 95% confidence interval.

12.5 Analysis of Secondary and Exploratory Endpoints

Secondary endpoints include subsequent surgical resectability, pathologic response rates, DFS and overall survival. All subjects who receive at least one dose of study drug will be included in the analysis of secondary endpoints. DFS (for resectable patients) and OS will be described using the Kaplan-Meier method with 95% confidence intervals. Surgical resectability after neoadjuvant therapy as determined by our pancreatic multidisciplinary group based upon NCCN guidelines for resectability.[47] Pathologic response will be determined by pathologic examination. Proportions of surgical resectability and pathologic response will be reported along with the corresponding binomial exact 95% confidence intervals.

Genomic sequencing library construction, whole genome/exome sequencing, whole transcriptome sequencing, microbial sequencing, neoepitope prediction, mutation burden, and bioinformatic analysis will be performed either at an on-campus laboratory or at an off-campus sequencing service. All the samples will be de-identified before

sending to any laboratory for sequencing. The FASTQ files, BAM files and VCF files will be generated and analyzed.

Results from the sequencing studies will not be released to the patients. These studies are for research purposes only and are not using a clinically validated platform.

Genomic sequencing data will be either destroyed or stored on a JHU managed, HIPAA-compliant, password protected hard drive or Johns Hopkins University School of Medicine PMAP.

12.6 Biomarker Analysis

Potential relationships between biomarker data and efficacy or safety endpoints will be investigated as part of an analysis plan aimed at identifying baseline biomarkers that may be used to prospectively identify subjects likely (or not likely) to respond to combination pembrolizumab, CY, GVAX, and IMC-CS4 and to identify subjects who may be predisposed to having adverse reactions to treatment. These exploratory predictive biomarker analyses will be completed with biomarkers measured in blood and in tumor samples and will focus on factors outlined in the exploratory objectives. Similar analyses will be completed with peripheral blood samples. We will also explore standard protein biomarkers such as CA19-9 and other exploratory circulating biomarkers. The evaluable population includes all subjects who receive at least one dose of study drug and have biomarker measures.

Continuous variables will be summarized with means or medians and standard deviations. Dichotomous and categorical variables will be summarized using proportions with exact 95% confidence intervals and counts, respectively. Summaries for both pre and post administration of each immunotherapy will be computed. Plots will be used to show the changes in immune response over time.

Comparison in pre- and post-treatment biomarker data will be compared using paired t-tests (or Wilcoxon signed rank tests if appropriate) and McNemar's tests for dichotomous or categorical variables. Associations between immune parameters will be explored graphically (e.g. scatterplots, boxplots) and numerically (e.g. correlations, Fisher's exact tests). Mixed effects models will be used to assess the patterns in biomarkers over time.

Efficacy measures will include OS, objective response, pathologic response, immune criteria of response, and DFS. Demographic and case-history factors will be examined to determine whether stratification or adjustments should be made within the subsequent statistical analyses, and if necessary, the appropriate stratification or adjustment will be made. Biomarkers will be summarized graphically as they relate to efficacy and safety endpoints, as applicable. Summary statistics will be tabulated. The relationships between binary measures (e.g. response) and time to event outcomes (e.g.

OS) and candidate biomarkers will be investigated using logistic regression and Cox proportional hazards regression, respectively. Associations will be summarized in terms of point and interval estimates of hazard ratios, odds ratios, or other statistics, as appropriate for the analyses completed. Models to predict clinical activity based on combinations of biomarkers may also be investigated.

The significance level is set at 0.05 for all tests in the exploratory biomarker analyses unless otherwise specified.

REFERENCES

1. Siegel R, Ma J, Zou Z, Jemal A. Cancer statistics, 2014. CA Cancer J Clin. 2014;64: 9-29.
2. Ahlgren JD. Epidemiology and risk factors in pancreatic cancer. Semin Oncol. 1996;23: 241-250.
3. Wangjam T, Zhang Z, Zhou XC, et al. Resected pancreatic ductal adenocarcinomas with recurrence limited in lung have a significantly better prognosis than those with other recurrence patterns. Oncotarget. 2015;6: 36903-36910.
4. Vauthey JN, Dixon E. AHPBA/SSO/SSAT Consensus Conference on Resectable and Borderline Resectable Pancreatic Cancer: rationale and overview of the conference. Ann Surg Oncol. 2009;16: 1725-1726.
5. Dholakia AS, Hacker-Prietz A, Wild AT, et al. Resection of borderline resectable pancreatic cancer after neoadjuvant chemoradiation does not depend on improved radiographic appearance of tumor-vessel relationships. J Radiat Oncol. 2013;2: 413-425.
6. Evans DB, Varadhachary GR, Crane CH, et al. Preoperative gemcitabine-based chemoradiation for patients with resectable adenocarcinoma of the pancreatic head. J Clin Oncol. 2008;26: 3496-3502.
7. Varadhachary GR, Wolff RA, Crane CH, et al. Preoperative gemcitabine and cisplatin followed by gemcitabine-based chemoradiation for resectable adenocarcinoma of the pancreatic head. J Clin Oncol. 2008;26: 3487-3495.
8. Jaffee EM, Schutte M, Gossett J, et al. Development and characterization of a cytokine-secreting pancreatic adenocarcinoma vaccine from primary tumors for use in clinical trials. Cancer J Sci Am. 1998;4: 194-203.
9. Jaffee EM, Hruban RH, Biedrzycki B, et al. Novel allogeneic granulocyte-macrophage colony-stimulating factor-secreting tumor vaccine for pancreatic cancer: a phase I trial of safety and immune activation. J Clin Oncol. 2001;19: 145-156.
10. Berns AJ, Clift S, Cohen LK, et al. Phase I study of non-replicating autologous tumor cell injections using cells prepared with or without GM-CSF gene transduction in patients with metastatic renal cell carcinoma. Hum Gene Ther. 1995;6: 347-368.
11. Lim M, Simons JW. Emerging concepts in GM-CSF gene-transduced tumor vaccines for human prostate cancer. Curr Opin Mol Ther. 1999;1: 64-71.
12. Soiffer R, Hodi FS, Haluska F, et al. Vaccination with irradiated, autologous melanoma cells engineered to secrete granulocyte-macrophage colony-stimulating factor by adenoviral-mediated gene transfer augments antitumor immunity in patients with metastatic melanoma. J Clin Oncol. 2003;21: 3343-3350.
13. Dranoff G, Jaffee E, Lazenby A, et al. Vaccination with irradiated tumor cells engineered to secrete murine granulocyte-macrophage colony-stimulating factor stimulates potent, specific, and long-lasting anti-tumor immunity. Proc Natl Acad Sci U S A. 1993;90: 3539-3543.
14. Lutz E, Yeo CJ, Lillemoe KD, et al. A lethally irradiated allogeneic granulocyte-macrophage colony stimulating factor-secreting tumor vaccine for pancreatic adenocarcinoma. A Phase II trial of safety, efficacy, and immune activation. Ann Surg. 2011;253: 328-335.
15. Drake CG, Jaffee E, Pardoll DM. Mechanisms of immune evasion by tumors. Adv Immunol. 2006;90: 51-81.
16. Woo EY, Chu CS, Goletz TJ, et al. Regulatory CD4(+)CD25(+) T cells in tumors from patients with early-stage non-small cell lung cancer and late-stage ovarian cancer. Cancer Res. 2001;61:

4766-4772.
17. Liyanage UK, Moore TT, Joo HG, et al. Prevalence of regulatory T cells is increased in peripheral blood and tumor microenvironment of patients with pancreas or breast adenocarcinoma. J Immunol. 2002;169: 2756-2761.
18. Curiel TJ, Coukos G, Zou L, et al. Specific recruitment of regulatory T cells in ovarian carcinoma fosters immune privilege and predicts reduced survival. Nat Med. 2004;10: 942-949.
19. Ercolini AM, Ladle BH, Manning EA, et al. Recruitment of latent pools of high-avidity CD8(+) T cells to the antitumor immune response. J Exp Med. 2005;201: 1591-1602.
20. Laheru D, Lutz E, Burke J, et al. Allogeneic granulocyte macrophage colony-stimulating factor-secreting tumor immunotherapy alone or in sequence with cyclophosphamide for metastatic pancreatic cancer: a pilot study of safety, feasibility, and immune activation. Clin Cancer Res. 2008;14: 1455-1463.
21. Lutz ER, Wu AA, Bigelow E, et al. Immunotherapy converts nonimmunogenic pancreatic tumors into immunogenic foci of immune regulation. Cancer Immunol Res. 2014;2: 616-631.
22. Soares KC, Rucki AA, Wu AA, et al. PD-1/PD-L1 blockade together with vaccine therapy facilitates effector T-cell infiltration into pancreatic tumors. J Immunother. 2015;38: 1-11.
23. Disis ML. Immune regulation of cancer. J Clin Oncol. 2010;28: 4531-4538.
24. Dudley ME, Wunderlich JR, Yang JC, et al. Adoptive cell transfer therapy following non-myeloablative but lymphodepleting chemotherapy for the treatment of patients with refractory metastatic melanoma. J Clin Oncol. 2005;23: 2346-2357.
25. Hunder NN, Wallen H, Cao J, et al. Treatment of metastatic melanoma with autologous CD4+ T cells against NY-ESO-1. N Engl J Med. 2008;358: 2698-2703.
26. Francisco LM, Sage PT, Sharpe AH. The PD-1 pathway in tolerance and autoimmunity. Immunol Rev. 2010;236: 219-242.
27. Brown JA, Dorfman DM, Ma FR, et al. Blockade of programmed death-1 ligands on dendritic cells enhances T cell activation and cytokine production. J Immunol. 2003;170: 1257-1266.
28. Dong H, Strome SE, Salomao DR, et al. Tumor-associated B7-H1 promotes T-cell apoptosis: a potential mechanism of immune evasion. Nat Med. 2002;8: 793-800.
29. Sharpe AH, Freeman GJ. The B7-CD28 superfamily. Nat Rev Immunol. 2002;2: 116-126.
30. Yoshimura K, Laird LS, Chia CY, et al. Live attenuated Listeria monocytogenes effectively treats hepatic colorectal cancer metastases and is strongly enhanced by depletion of regulatory T cells. Cancer Res. 2007;67: 10058-10066.
31. Nomi T, Sho M, Akahori T, et al. Clinical significance and therapeutic potential of the programmed death-1 ligand/programmed death-1 pathway in human pancreatic cancer. Clin Cancer Res. 2007;13: 2151-2157.
32. Gao Q, Wang XY, Qiu SJ, et al. Overexpression of PD-L1 significantly associates with tumor aggressiveness and postoperative recurrence in human hepatocellular carcinoma. Clin Cancer Res. 2009;15: 971-979.
33. Hamanishi J, Mandai M, Iwasaki M, et al. Programmed cell death 1 ligand 1 and tumor-infiltrating CD8+ T lymphocytes are prognostic factors of human ovarian cancer. Proc Natl Acad Sci U S A. 2007;104: 3360-3365.
34. Fourcade J, Kudela P, Sun Z, et al. PD-1 is a regulator of NY-ESO-1-specific CD8+ T cell expansion in melanoma patients. J Immunol. 2009;182: 5240-5249.
35. Lipson EJ, Sharfman WH, Drake CG, et al. Durable cancer regression off-treatment and effective reinduction therapy with an anti-PD-1 antibody. Clin Cancer Res. 2013;19: 462-468.

36. Hume DA, MacDonald KP. Therapeutic applications of macrophage colony-stimulating factor-1 (CSF-1) and antagonists of CSF-1 receptor (CSF-1R) signaling. Blood. 2012;119: 1810-1820.
37. Wynn TA, Chawla A, Pollard JW. Macrophage biology in development, homeostasis and disease. Nature. 2013;496: 445-455.
38. DeNardo DG, Brennan DJ, Rexhepaj E, et al. Leukocyte complexity predicts breast cancer survival and functionally regulates response to chemotherapy. Cancer Discov. 2011;1: 54-67.
39. Zhu Y, Knolhoff BL, Meyer MA, et al. CSF1/CSF1R blockade reprograms tumor-infiltrating macrophages and improves response to T-cell checkpoint immunotherapy in pancreatic cancer models. Cancer Res. 2014;74: 5057-5069.
40. Anthony SP, Puzanov I, Lin PS, Nolop KB, West B, Hoff DDV. Pharmacodynamic activity demonstrated in phase I for PLX3397, a selective inhibitor of FMS and Kit. Journal of Clinical Oncology. 2011;29: 3093-3093.
41. Smyrk TC, Watson P, Kaul K, Lynch HT. Tumor-infiltrating lymphocytes are a marker for microsatellite instability in colorectal carcinoma. Cancer. 2001;91: 2417-2422.
42. Le DT, Lutz E, Uram JN, et al. Evaluation of ipilimumab in combination with allogeneic pancreatic tumor cells transfected with a GM-CSF gene in previously treated pancreatic cancer. J Immunother. 2013;36: 382-389.
43. Bigelow E, Bever KM, Xu H, et al. Immunohistochemical staining of B7-H1 (PD-L1) on paraffin-embedded slides of pancreatic adenocarcinoma tissue. J Vis Exp. 2013.
44. Cameron JL, Riall TS, Coleman J, Belcher KA. One thousand consecutive pancreaticoduodenectomies. Ann Surg. 2006;244: 10-15.
45. Clavien PA, Barkun J, de Oliveira ML, et al. The Clavien-Dindo classification of surgical complications: five-year experience. Ann Surg. 2009;250: 187-196.
46. Blair AB, Rosati LM, Rezaee N, et al. Postoperative complications after resection of borderline resectable and locally advanced pancreatic cancer: The impact of neoadjuvant chemotherapy with conventional radiation or stereotactic body radiation therapy. Surgery. 2018;163: 1090-1096.
47. Katz MH, Marsh R, Herman JM, et al. Borderline resectable pancreatic cancer: need for standardization and methods for optimal clinical trial design. Ann Surg Oncol. 2013;20: 2787-2795.
48. Wolchok JD, Hoos A, O'Day S, et al. Guidelines for the evaluation of immune therapy activity in solid tumors: immune-related response criteria. Clin Cancer Res. 2009;15: 7412-7420.

APPENDIX A: Performance Status Criteria

<table>
<tr><th colspan="2">ECOG Performance Status Scale</th><th colspan="2">Karnofsky Performance Scale</th></tr>
<tr><th>Grade</th><th>Descriptions</th><th>Percent</th><th>Description</th></tr>
<tr><td rowspan="2">0</td><td rowspan="2">Normal activity. Fully active, able to carry on all pre-disease performance without restriction.</td><td>100</td><td>Normal, no complaints, no evidence of disease.</td></tr>
<tr><td>90</td><td>Able to carry on normal activity; minor signs or symptoms of disease.</td></tr>
<tr><td rowspan="2">1</td><td rowspan="2">Symptoms, but ambulatory. Restricted in physically strenuous activity, but ambulatory and able to carry out work of a light or sedentary nature (e.g., light housework, office work).</td><td>80</td><td>Normal activity with effort; some signs or symptoms of disease.</td></tr>
<tr><td>70</td><td>Cares for self, unable to carry on normal activity or to do active work.</td></tr>
<tr><td rowspan="2">2</td><td rowspan="2">In bed <50% of the time. Ambulatory and capable of all self-care, but unable to carry out any work activities. Up and about more than 50% of waking hours.</td><td>60</td><td>Requires occasional assistance, but is able to care for most of his/her needs.</td></tr>
<tr><td>50</td><td>Requires considerable assistance and frequent medical care.</td></tr>
<tr><td rowspan="2">3</td><td rowspan="2">In bed >50% of the time. Capable of only limited self-care, confined to bed or chair more than 50% of waking hours.</td><td>40</td><td>Disabled, requires special care and assistance.</td></tr>
<tr><td>30</td><td>Severely disabled, hospitalization indicated. Death not imminent.</td></tr>
<tr><td rowspan="2">4</td><td rowspan="2">100% bedridden. Completely disabled. Cannot carry on any self-care. Totally confined to bed or chair.</td><td>20</td><td>Very sick, hospitalization indicated. Death not imminent.</td></tr>
<tr><td>10</td><td>Moribund, fatal processes progressing rapidly.</td></tr>
<tr><td>5</td><td>Dead.</td><td>0</td><td>Dead.</td></tr>
</table>

APPENDIX B: Adverse Event of Clinical Interest (ECI) Reporting Form

Adverse Event of Clinical Interest (ECI) Reporting Form

Please notify: IND Sponsor within 24 hours (email: ejaffee@jhmi.edu)
Merck within 24 hours (Fax: +1-215-993-1220)
Eli Lilly within 24 hours (Fax: +1-866-644-1697)

<table>
<tr><td>Protocol Title:</td><td colspan="3">A Pilot Study of a GVAX Pancreas Vaccine (with Cyclophosphamide) in Combination with a PD-1 Blockade Antibody (Pembrolizumab) and a Macrophage Targeting Agent (CSF1R inhibitor) for the Treatment of Patients with Borderline Resectable Adenocarcinoma of the Pancreas</td></tr>
<tr><td>Protocol Number:
J1766
MK-3475-536</td><td>Signature of PI:</td><td>Principal Investigator:</td><td>Date:</td></tr>
<tr><td colspan="4">Report Type:
☐Initial ☐Follow-up ☐Final Follow-up ☐Addendum to:</td></tr>
<tr><td colspan="4">Section A: Subject Information</td></tr>
<tr><td>Subject ID:</td><td colspan="2">Subject Initial:</td><td>Subject Gender:
☐Male
☐Female</td></tr>
<tr><td colspan="4">Section B: Event Information</td></tr>
</table>

<table>
<tr><td rowspan="7">Event diagnosis or symptoms:</td><td colspan="4">Date of First Dose:</td><td rowspan="7">Action taken with the study drug:
☐None
☐Interrupted
☐Discontinued
☐Delayed</td></tr>
<tr><td>Pembrolizumab</td><td>CY</td><td>GVAX</td><td>IMC-CS4</td></tr>
<tr><td colspan="4">Date of Last Dose Prior to Event:</td></tr>
<tr><td>Pembrolizumab</td><td>CY</td><td>GVAX</td><td>IMC-CS4</td></tr>
<tr><td colspan="4">Number of Total Doses:</td></tr>
<tr><td>Pembrolizumab</td><td>CY</td><td>GVAX</td><td>IMC-CS4</td></tr>
<tr><td></td><td></td><td></td><td></td></tr>
<tr><td>Event Onset Date:</td><td colspan="3">Event End Date:</td><td colspan="2">Date Event Discovered:</td></tr>
</table>

Relationship to:	Pembro lizumab	CY	GVAX	IMC-CS4	Underlying Disease
Unrelated	☐	☐	☐	☐	☐
Related	☐	☐	☐	☐	☐

Section C: Brief Description of the Event: (please include relevant procedures and laboratory values)

Section D: Relevant Medical History

Section E: Concomitant Drug (Not related to ECI)

Name of the Drug	Start Date	Stop Date	Route	Dose	Frequency

Section F: Comments

Additional Documents: ☐ Please specify

APPENDIX C: SAE Reporting Form

Serious Adverse Event Reporting Form

Please notify: IND Sponsor within 24 hours (email: ejaffee@jhmi.edu)
Merck within 24 hours (Fax: +1-215-993-1220)
Eli Lilly within 24 hours (Fax: 1-866-644-1697)

<table>
<tr><td>Protocol Title:</td><td colspan="3">A Pilot Study of a GVAX Pancreas Vaccine (with Cyclophosphamide) in Combination with a PD-1 Blockade Antibody (Pembrolizumab) and a Macrophage Targeting Agent (CSF1R inhibitor) for the Treatment of Patients with Borderline Resectable Adenocarcinoma of the Pancreas</td></tr>
<tr><td>Protocol Number:
J1766
MK-3475-536</td><td>Signature of PI:</td><td>Principal Investigator:</td><td>Date:</td></tr>
<tr><td>Report Type:
☐Initial
☐Follow-up
☐Final Follow-up
☐Death
☐Addendum to:</td><td>Serious Criteria (check all that apply):
☐Death
☐Life-threatening
☐ Hospitalization or Elongation of Existing Hospitalization
☐ Persistent or Significant Disability
☐ Congenital Anomaly
☐ Other Important Medical Event
☐ Cancer
☐ Overdose</td><td>Hospital Admission Date:

Hospital Discharge Date:</td><td>Date Event Discovered:</td></tr>
</table>

<table>
<tr><td colspan="3">Section A: Subject Information</td></tr>
<tr><td>Subject ID:</td><td>Subject Initial:</td><td>Subject Gender:
☐Male
☐Female</td></tr>
</table>

<table>
<tr><td colspan="6">Section B: Event Information</td></tr>
<tr><td rowspan="6">Event diagnosis or symptoms:</td><td colspan="4">Date of First Dose:</td><td rowspan="6">Action taken with the study drug:

☐None
☐Interrupted
☐Discontinued
☐Delayed</td></tr>
<tr><td>Pembrolizumab</td><td>CY</td><td>GVAX</td><td>IMC-CS4</td></tr>
<tr><td colspan="4">Date of Last Dose Prior to Event:</td></tr>
<tr><td>Pembrolizumab</td><td>CY</td><td>GVAX</td><td>IMC-CS4</td></tr>
<tr><td colspan="4">Number of Total Doses:</td></tr>
<tr><td>Pembrolizumab</td><td>CY</td><td>GVAX</td><td>IMC-CS4</td></tr>
</table>

Event Onset Date:					Event End Date:
**Relationship to:**	**Pembro lizumab**	**CY**	**GVAX**	**IMC-CS4**	**Underlying Disease**
**Unrelated**	☐	☐	☐	☐	☐
**Related**	☐	☐	☐	☐	☐

Section C: Brief Description of the Event:

Section D: Relevant Medical History

Section E: Concomitant Drug (Not related to SAE)

Name of the Drug	Start Date	Stop Date	Route	Dose	Frequency

Section F: Comments

Additional Documents: ☐ Please specify

APPENDIX D: Immune Related Response Criteria

For all patients who experience disease progression on study, the date noted for of disease progression is the time of the scan where it is originally detected, and not the following date of the confirmatory scan.

Definitions of measurable and non-measurable disease

Measurable disease: Neoplastic masses that can be precisely measured in 2 in-plane perpendicular diameters. Both its longest diameter and its longest perpendicular must be greater than or equal to 10 mm. Lymph nodes must have a short-axis line-length of ≥ 15 mm. Malignant lymph nodes must be measurable in 2 perpendicular diameters. Both its longest diameter and its longest perpendicular must be greater than or equal to 15 mm. The quantitative endpoint will be defined as the product of the longest diameter with its longest perpendicular.

Non-measurable disease: Non-measurable lesions are those that are not suitable for quantitative assessment over time. These include:

1) Neoplastic masses that are too small to measure, because their longest uninterrupted diameter or longest perpendicular are less than 10 mm.

2) Neoplastic masses whose boundaries cannot be distinguished. This includes masses which cannot be demarcated from surrounding tissue because of inadequate contrast, masses with overly complex morphology, or those with highly heterogeneous tissue composition.

3) Other types of lesions that are confidently felt to represent neoplastic tissue, but difficult to quantify in a reproducible manner. These include bone metastases, leptomeningeal metastases, malignant ascites, pleural/pericardial effusions, inflammatory breast disease, lymphangitis cutis/pulmonis, cystic lesions, ill-defined abdominal masses, etc.

For irRC, only target lesions selected at baseline and measurable new lesions are taken into account.

At the baseline tumor assessment, the sum of the products of the two largest perpendicular diameters (SPD) of all **index lesions** (five lesions per organ, up to 10 visceral lesions and five cutaneous index lesions) is calculated.

At each subsequent tumor assessment, the SPD of the index lesions and of new, measurable lesions (≥ 5 X 5 mm; up to 5 new lesions per organ: 5 new cutaneous lesions and 10 visceral lesions) are added together to provide the total time-point **tumor burden.**

Overall response using irRC:

- **Complete Response (irCR):** Complete disappearance of all tumor lesions (whether measureable or not, and no new lesions). CR must be confirmed by repeated, consecutive assessments made no less than 4 weeks from the date first documented.

- **Partial Response (irPR):** Decrease in SPD of 50% or greater by a consecutive assessment at least 4 weeks after first documentation.

- **Stable Disease (irSD):** Failure to meet criteria for irCR or irPR, in absence of irPD.

- **Progressive Disease (irPD):** At least 25% increase in SPD relative to nadir (minimum recorded tumor burden) Confirmation by a repeat, consecutive assessment no less than 4 weeks from the data first documented.

Please note other key differences between irRC and the original WHO criteria:

New measurable lesions will be incorporated into the SPD

New non measurable lesions do not define progression but preclude irCR

Non-index lesions contribute to defining irCR (complete disappearance required).

See the Investigators Imaging Operations Manual (IIOM) for more details.

REFERENCE

IrRC for the current protocol is adopted from the following reference[48]
