# Supplementary material for: Safety and immunologic impact of neoadjuvant/adjuvant GVAX, cyclophosphamide, pembrolizumab, and anti-CSF1R agent IMC-CS4 in pancreatic adenocarcinoma
Source: Front Immunol. 2026 Mar 9;17:1715761. doi: 10.3389/fimmu.2026.1715761 (PMC13006681; doi:10.3389/fimmu.2026.1715761)
Supplement: Supplementary file 2 [file DataSheet2.pdf]

Supplementary Table 1

The multiplexed IHC Staining Panel

Cycle	1st Antibody	1st Antibody Clone	1st Antibody Vendor	1st Antibody Concentration	1st Antibody Incubation time (mins)	2nd Antibody Clone	2nd Antibody Incubation Time (mins)	AEC Reaction Time (mins)
Cycle 1	CD68	PG-M1	Abcam	1:50	30	Anti-mice	30	25-45
Cycle 2	PD1	NAT105	Abcam	1:50	120	Anti-mice	30	120-180
Cycle 3	PDL1	E1I3N	Cell signalling	1:100	120	Anti-rabbit	30	120-180
Cycle 4	CD163	10D6	Invitrogen	1:100	30	Anti-mice	30	20-30
Cycle 5	T-bet	SC-21749(4B/10)	SANTA CRUZ	1:100	30	Anti-mice	30	120-180
Cycle 6	Granzyme B	EP230	Cell Mrque	1:100	60	Anti-rabbit	30	240-360
Cycle 7	CSF1R	SP211	Abcam	1:150	30	Anti-rabbit	30	60-120
Cycle 8	Foxp3	236A/E7	eBioscience	1:40	30	Anti-mice	30	120
Cycle 9	CD4	4B12	Invitrogen	1:25	120	Anti-mice	30	60-120
Cycle 10	CD8	C8/144b	eBioscience	1:100	30	Anti-mice	30	45
Cycle 11	CD66B	G10f5	Novus	1:600	30	Anti-mice	30	30
Cycle 12	CD3	Sp7	Thermo Science	1:150	30	Anti-rabbit	30	100
Cycle 13	CD20	0.N.85	Sanata Cruz	1:400	30	Anti-mice	30	60
Cycle 14	CD45	H130	BD	1:100	30	Anti-mice	30	45-60
Cycle 15	CD56	123c3	Invitrogen	1:25	30	Anti-rabbit	30	25

Supplementary Table 2

The Gating Strategy for Immune Cell Subtypes of mIHC

Cell Types	Adaptation according to available markers
CD8+ T cells	CD45+CSF1R(-)CD3+CD8+
CD8+ Granzyme B+ T cells	CD45+CSF1R(-)CD3+CD8+GranzymeB+
CD4+ T cells	CD45+CSF1R(-)CD3+CD4+
CD4+ PD-1+ T cells	CD45+CSF1R(-)CD3+CD4+PD-1+
Regulatory T cells	CD45+CSF1R(-)CD3+CD4+FOXP3+
B cells	CD45+CSF1R(-)CD3+CD20+
Tumor associated macrophage (TAM)	CD45+CD3(-)CD20(-)CD66b(-)CSF1R+
PD-L1+ CSF1R+ cells	CD45+CD3(-)CD20(-)CD66b(-)CSF1R+PD-L1+
M1 TAM	CD45+CD3(-)CD20(-)CD66b(-)CSF1R+CD68+CD163(-)
PD-L1+ M1 TAM	CD45+CD3(-)CD20(-)CD66b(-)CSF1R+CD68+CD163(-)PD-L1+
M2 TAM	CD45+CD3(-)CD20(-)CD66b(-)CSF1R+CD68+CD163+
PD-L1+ M2 TAM	CD45+CD3(-)CD20(-)CD66b(-)CSF1R+CD68+CD163+PD-L1+
CD66b+ Granulocytes(Gr)	CD45+CD3(-)CD20(-)CD66b+
PD-L1+ Gr	CD45+CD3(-)CD20(-)CD66b+PD-L1+

Supplementary Table 3

ID	DFS Time (Months)	DFS Status (1 = Event, 0 = Censored)	OS Time (Months)	OS Status (1 = Event, 0 = Censored)
P1	12.6	1	20.4	1
P2	5.6	1	18.7	1
P3	6.4	1	7.2	1
P4	12.8	1	22.4	1
P5	37.8	0	44.6	0
P6	23.4	1	44.4	0
P7	7.3	1	10.3	1
P8	8.0	1	17.5	1
P9	14.4	1	25.1	1
