# Supplementary material for: Safety and immunologic impact of neoadjuvant/adjuvant GVAX, cyclophosphamide, pembrolizumab, and anti-CSF1R agent IMC-CS4 in pancreatic adenocarcinoma
Source: Front Immunol. 2026 Mar 9;17:1715761. doi: 10.3389/fimmu.2026.1715761 (PMC13006681; doi:10.3389/fimmu.2026.1715761)
Supplement: Supplementary file 3 [file DataSheet3.pdf]

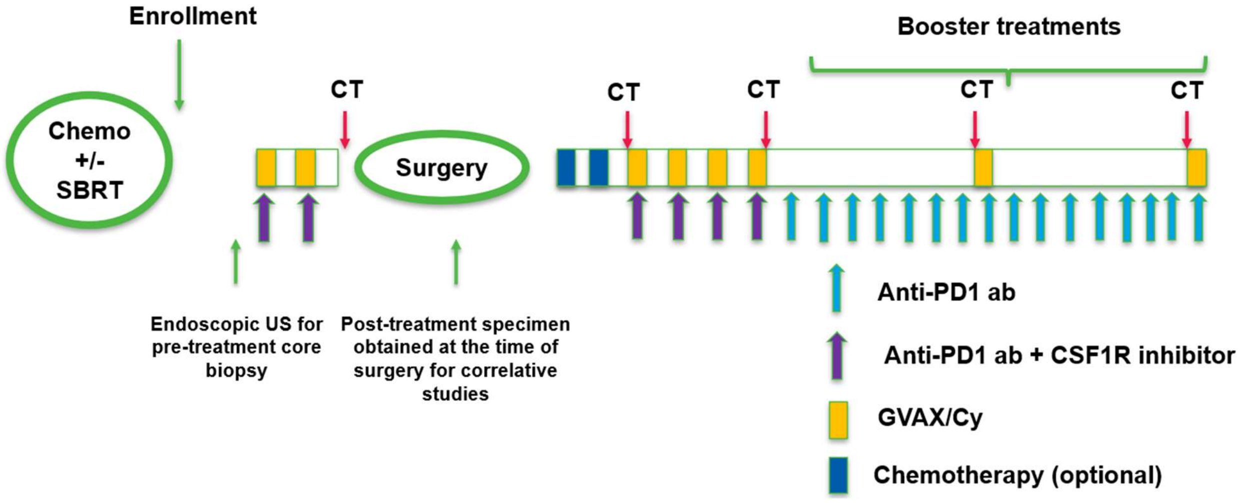

Figure 1: Study schema

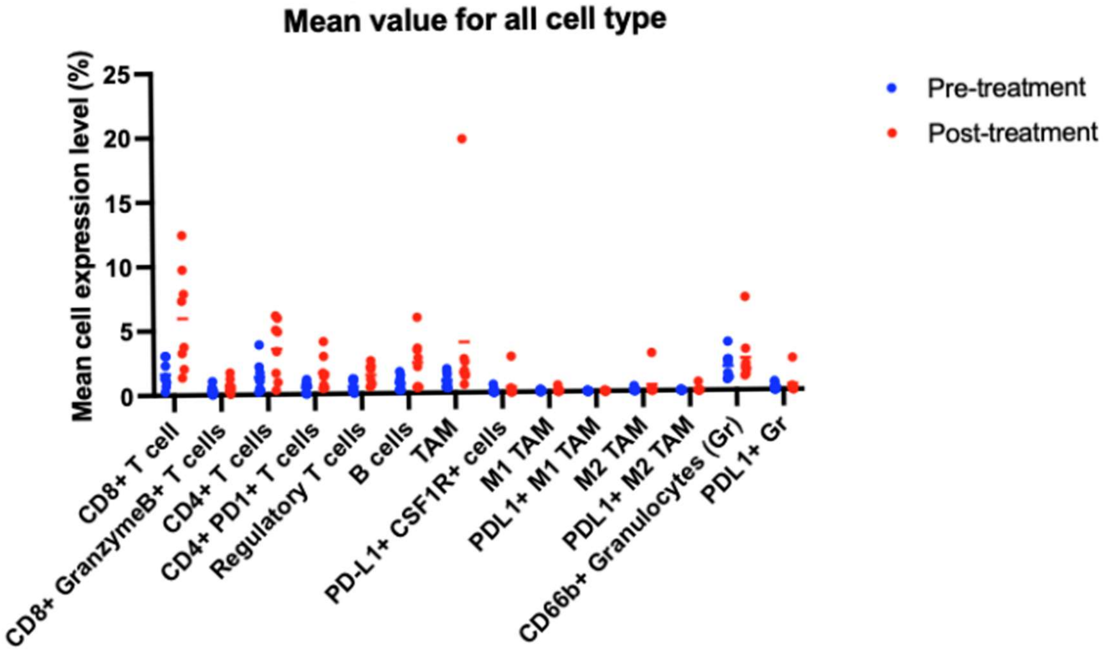

Figure 2: Mean density of immune cell subtypes

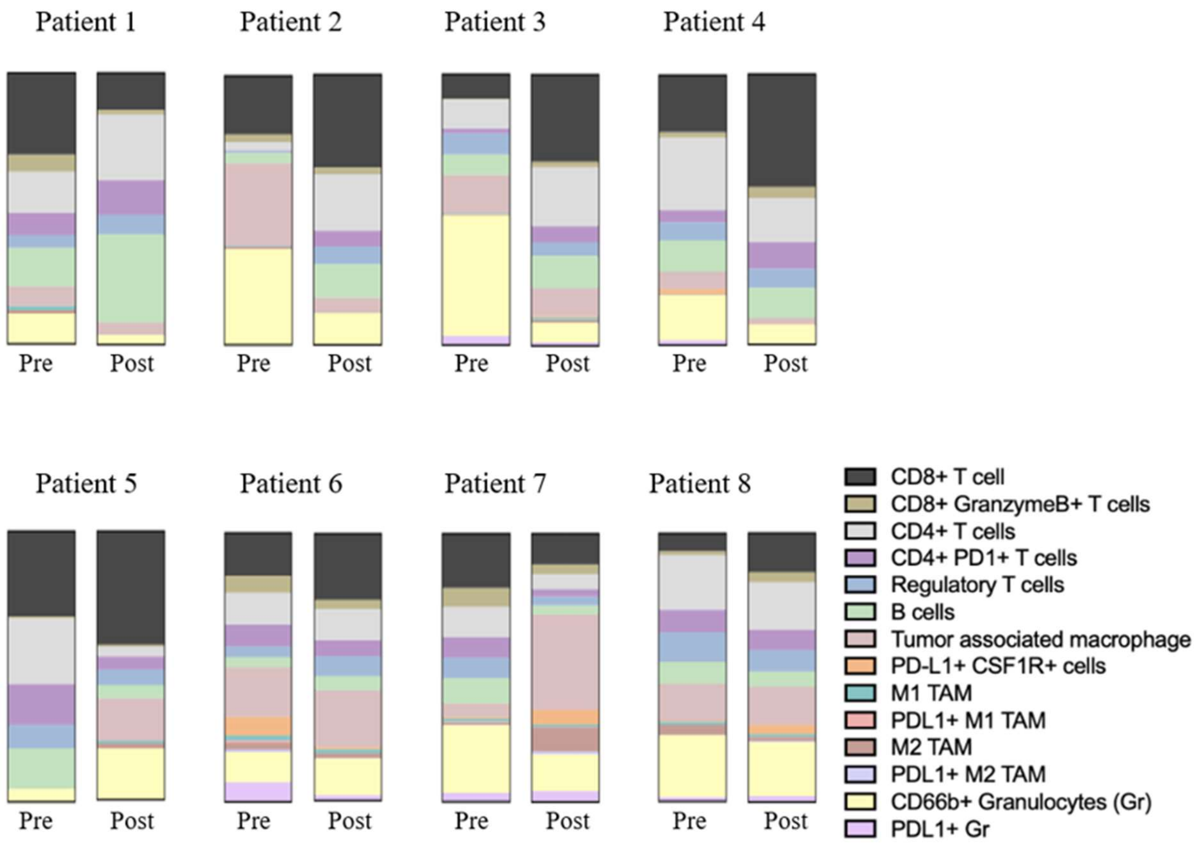

Figure 3: Composition of immune cell subtypes before and after treatment

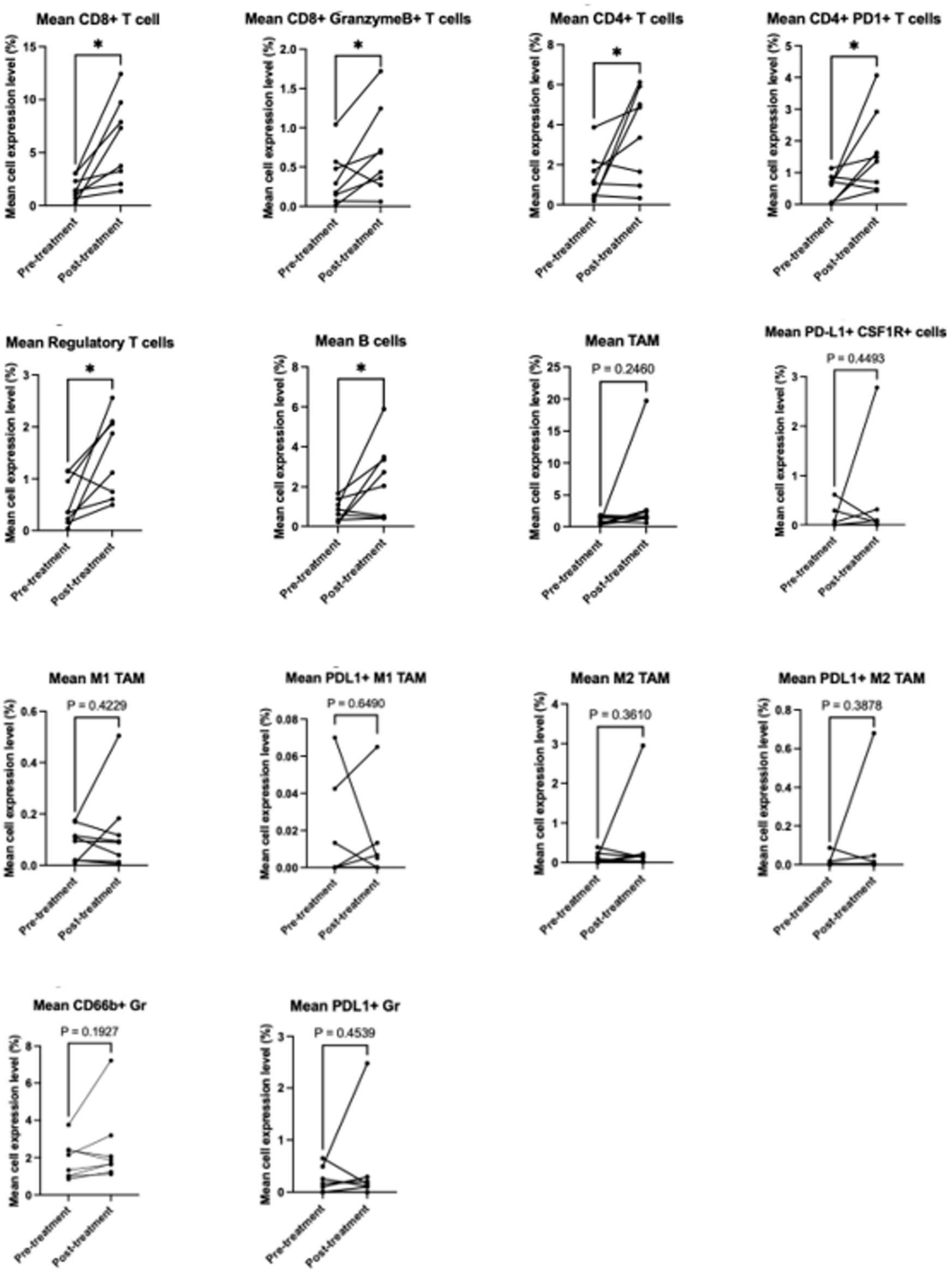

Note. * $p < 0.05$, ** $p < 0.01$, *** $p < 0.001$

Figure 4: Paired t-test comparing the mean density for each immune cell subtype before and after treatment

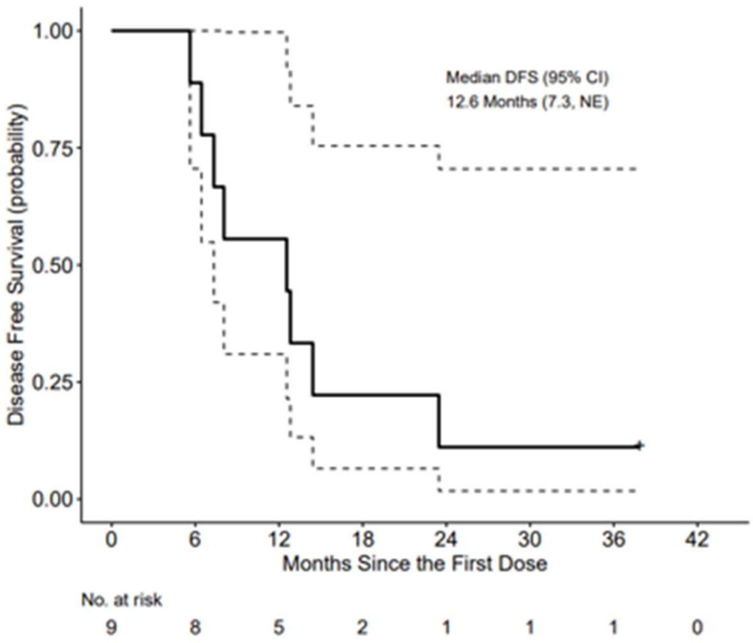

Figure 5: Disease free survival (DFS) is defined as time from First Dose to Progression/Death Date, censored at last scan date if alive or death date > 3 months after last scan.

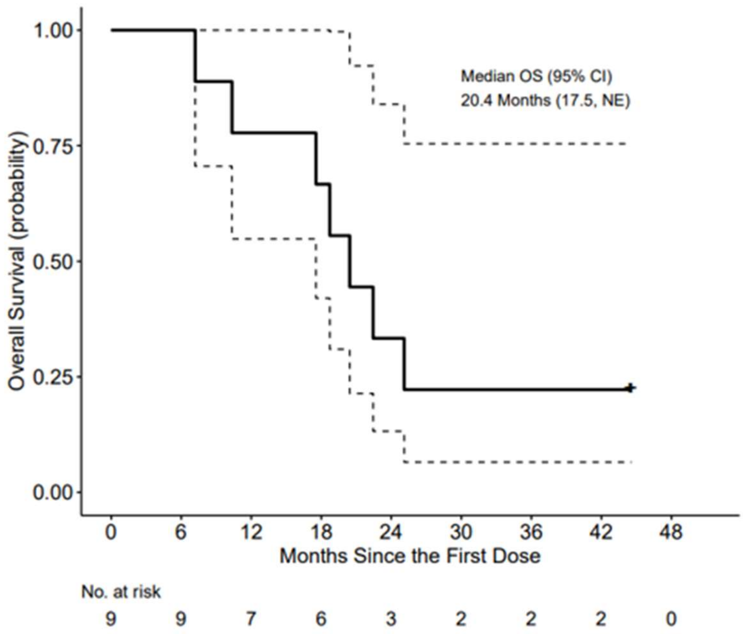

Figure 6: Overall Survival (OS) is defined as time from First Dose to Death, censored at date of last known alive.

Table 1: Demographics/patient characteristics

Median age (range) – yr	64 (47-75)
Male sex – no. (%)	4 (44%)
Race – no. (%)	
Asian	1 (11%)
White	7 (78%)
Hispanic/Latino	1 (11%)
Disease stage at diagnosis – no. (%)	
I	2 (22%)
II	0 (0%)
III	7 (78%)
Chemotherapy received – no. (%)	
FOLFIRINOX	7 (78%)
Gemcitabine and nab-paclitaxel	2 (22%)
Prior radiation – no. (%)	
SBRT	7 (78%)
None	2 (22%)
Clinical staging - no. (%)	
Resectable	2 (22%)
Borderline resectable	7 (78%)

Table 2: Treatment-related AE Table:

System organ Class Preferred Term	Grade 1/2	Grade 3/4	Total (N= 9) N (%)
**Blood and lymphatic system disorders**			
Anemia	1	0	1 (11%)
**Endocrine disorders**			
Hypothyroidism	1	0	1 (11%)
**Eye disorders**			
Periorbital edema	3	0	3 (33%)
**Gastrointestinal disorders**			
Diarrhea	1	1	2 (22%)
Nausea	1	0	1 (11%)
**General disorders**			
Chills	1	0	1 (11%)
Fatigue	1	0	1 (11%)
Fever	2	0	2 (22%)
Flu-like symptoms	1	0	1 (11%)
**Investigations**			
Alanine aminotransferase increased	2	0	2 (22%)
Aspartate aminotransferase increased	4	0	4 (44%)
CPK increased	1	0	1 (11%)
**Musculoskeletal and connective tissue disorders**			
Arthralgia	1	0	1 (11%)
**Skin and subcutaneous tissue disorders**			
Bruising, vaccine site	1	0	1 (11%)
Erythema, vaccine site	9	0	9 (100%)
Induration, vaccine site	8	0	8 (89%)
Pruritus, vaccine site	8	0	8 (89%)
Rash	3	1	4 (44%)
Warmth, vaccine site	3	0	3 (33%)

Table 3: Immune-related AE Table:

System organ Class Preferred Term	Grade 1/2	Grade 3/4	Total (N= 9) N (%)
**Endocrine disorders**			
Hypothyroidism	1	0	1 (11%)
**Gastrointestinal disorders**			
Diarrhea	1	1	1 (22%)
**Investigations**			
Alanine aminotransferase increased	1	0	1 (11%)
Aspartate aminotransferase increased	3	0	3 (33%)
**Skin and subcutaneous tissue disorders**			
Rash	3	1	4 (44%)

Table 4: Pathologic response using the tumor regression grading system for pancreatic ductal adenocarcinoma after neoadjuvant chemotherapy of the College of American Pathologists

Pathologic response grade – no. (%)	
0 – No viable tumor cells (complete response)	1 (11%)
1 – Single cells or rare groups of cancer cells (near complete response)	3 (33%)
2 – Residual cancer with evidence tumor regression, but more than single cells or rare groups of cancer cells (partial response)	3 (33%)
3 – Extensive residual cancer with no evident tumor regression (poor or no response)	2 (22%)
